# *N*- and *O*-arylation of pyridin-2-ones with diaryliodonium salts: base-dependent orthogonal selectivity under metal-free conditions†

**DOI:** 10.1039/d0sc02516j

**Published:** 2020-07-21

**Authors:** Masami Kuriyama, Natsumi Hanazawa, Yusuke Abe, Kotone Katagiri, Shimpei Ono, Kosuke Yamamoto, Osamu Onomura

**Affiliations:** Graduate School of Biomedical Sciences, Nagasaki University 1-14 Bunkyo-machi Nagasaki 852-8521 Japan mkuriyam@nagasaki-u.ac.jp onomura@nagasaki-u.ac.jp

## Abstract

Metal-free *N*- and *O*-arylation reactions of pyridin-2-ones as ambident nucleophiles have been achieved with diaryliodonium salts on the basis of base-dependent chemoselectivity. In the presence of *N*,*N*-diethylaniline in fluorobenzene, pyridin-2-ones were very selectively converted to *N*-arylated products in high yields. On the other hand, the *O*-arylation reactions smoothly proceeded with the use of quinoline in chlorobenzene, leading to high yields and selectivities. In these methods, a variety of pyridin-2-ones in addition to pyridin-4-one and a set of diaryliodonium salts were accepted as suitable reaction partners.

## Abstract 2

The metal-free *N*- and *O*-arylation reactions of pyridin-2-ones with diaryliodonium salts were achieved on the basis of base-dependent chemoselectivity.

## Graphical abstract

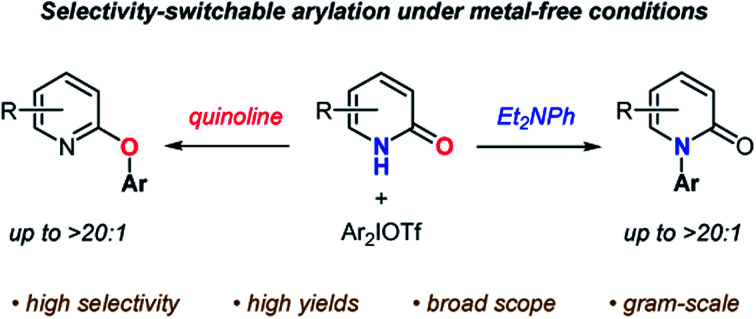

## Introduction

**Scheme 1:**
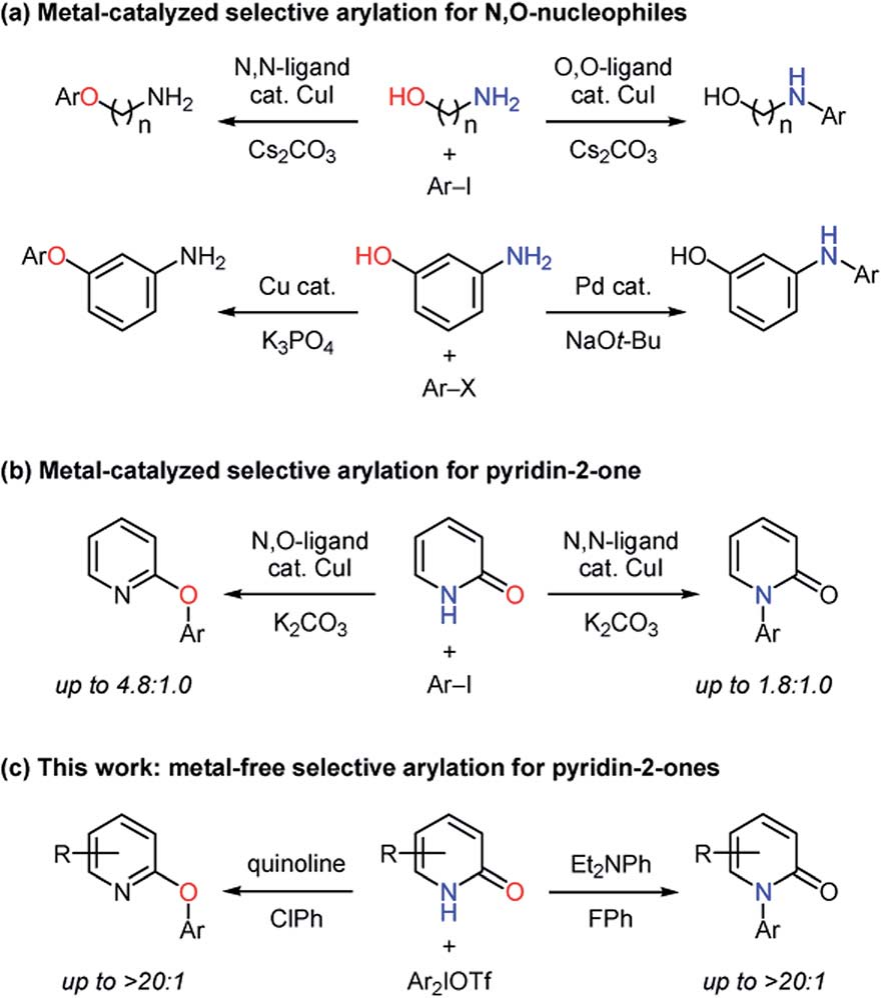
Selectivity-switchable arylation for *N*,*O*-nucleophiles.

Carbon–heteroatom bond forming reactions are one of the fundamental transformations in organic synthesis.^1^ Especially, *N*- and *O*-arylation reactions have been pursued with great vigor because of their wide utilization in pharmaceutical research and process development.^2,3^ Pyridin-2-ones are an ambident nucleophile containing an amide moiety and their arylated products such as *N*-aryl pyridin-2-ones and *O*-aryl 2-hydroxypyridines are important substructures in bioactive compounds.^4,5^ Therefore, *N*- and *O*-arylation reactions of pyridin-2-ones have been studied under catalytic and non-catalytic conditions.^6–8^ In the transition metal-catalyzed methods,^6^ the *O*-arylation was realized by using 6-substituted pyridin-2-ones to suppress the formation of *N*-arylated products. As a metal-free process, Mukaiyama achieved the *N*-arylation with organobismuth reagents.^7*a*^ While Gaunt reported the preparation of 2-phenoxypyridine with the use of diphenyliodonium fluoride,^7*c*^ Mo developed the metal-free *O*-arylation of 6-substituted pyridin-2-ones.^7*b*^ The utilization of appropriate catalysts and reagents is a promising approach to control chemoselectivity for ambident nucleophiles,^9,10^ and the orthogonal selectivity in arylation reactions of *N*,*O*-nucleophiles has been realized with transition-metal catalysts to give simple and useful methods.^11^ For example, the chemoselectivity in the formation of arylated aminophenols largely depended on the kind of catalyst metals,^11*a*^ while bidentate ligands for a copper catalyst played key roles in the selective arylation of aminoalcohols (Scheme 1a).^11*b*^ Buchwald found that copper catalysts had the ability to switch the selectivity for the *N*- and *O*-arylation of pyridin-2-one with aryl iodides (Scheme 1b).^12^ However, this type of selectivity-switchable arylation *via* cross-coupling for *N*,*O*-nucleophiles including aminoalcohols, aminophenols, and tautomerizable amides has not been developed under metal-free conditions in spite of its attractive potential in terms of green chemistry.^13^ Among hypervalent halogen compounds,^14^ diaryliodonium salts have recently received much attention as mild, less-toxic, and easily accessible arylating agents.^15^ By leveraging their beneficial features, transition metal-free carbon–heteroatom bond forming reactions have been actively pursued.^16,17^ Herein, we report metal-free *N*- and *O*-arylation of pyridin-2-ones with diaryliodonium salts based on base-dependent chemoselectivity (Scheme 1c).

## Results and discussion

At the outset, the effects of organic and inorganic bases were investigated in the phenylation of pyridin-2-one (**1a**) with diphenyliodonium triflate (**2a**) (Table 1). In the absence of bases, the *N*-phenylated product **3aa** and *O*-phenylated product **4aa** were obtained in low yields with no selectivity (entry 1). The examination of organic bases proved that the reaction conditions with pyridine gave the *O*-phenylated product **4aa** with a high selectivity despite a low yield, while the highly selective formation of the *N*-phenylated product **3aa** was observed with a high yield in the presence of DIPEA (entries 2–3). The use of DABCO and DBU afforded the mixtures of **3aa** and **4aa** with low selectivities (entries 4–5). In the screening of inorganic bases, the *N*-phenylated product **3aa** was obtained as a major product in moderate to good yields with low to moderate selectivities (entries 6–12). Sodium bicarbonate conduced to a better selectivity than the other inorganic bases only to give **3aa** and **4aa** in the ratio of 78 : 22 (entry 9).

**Table 1:** Initial screening of bases^a^

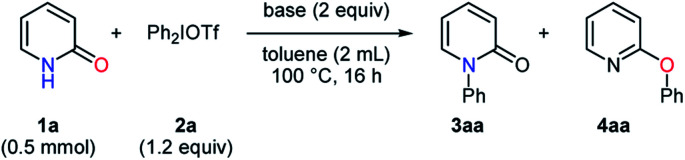				
Entry	Base	**3aa** (%)	**4aa** (%)	**3aa** : **4aa**
1	None	10	11	48 : 52
2	Pyridine	2	28	7 : 93
3	DIPEA	80	9	90 : 10
4	DABCO	4	2	67 : 33
5	DBU	57	24	70 : 30
6	Na_2_CO_3_	46	18	72 : 28
7	K_2_CO_3_	59	31	66 : 34
8	Cs_2_CO_3_	56	27	67 : 33
9	NaHCO_3_	70	20	78 : 22
10	KF	42	35	55 : 45
11	K_3_PO_4_	65	35	65 : 35
12	KO*t*-Bu	43	21	67 : 33

a Reaction conditions: **1a** (0.5 mmol), Ph_2_IOTf (1.2 equiv.), base (2 equiv.), toluene (2 mL), 100 °C, 16 h.

The findings of the initial screening encouraged us, and the optimization of conditions for the selective *N*-arylation was conducted at a higher concentration (Table 2). In the examination of tertiary amines, the *N*-phenylated product **3aa** was obtained in high yields and selectivities when using trialkyl amines such as DIPEA and *n*-Bu_3_N (entries 1–2). *N*,*N*-Diethylaniline led to a slightly better yield of **3aa**, while a decrease in yield was observed in the presence of *N*,*N*-dimethylaniline or *N*-methyldiphenylamine (entries 3–5). Then, a series of solvents were investigated with *N*,*N*-diethylaniline. More polar solvents exhibited a tendency to give **3aa** in lower yields (entries 3 and 6–8). The use of fluorobenzene resulted in 90% yield of **3aa** with an excellent selectivity, although **3aa** was formed in chlorobenzene with a slightly reduced yield (entries 9–10). DIPEA and *n*-Bu_3_N were also examined in fluorobenzene only to afford lower yields of **3aa** as compared to *N*,*N*-diethylaniline (entries 11–12).

**Table 2:** Optimization for *N*-arylation^a^

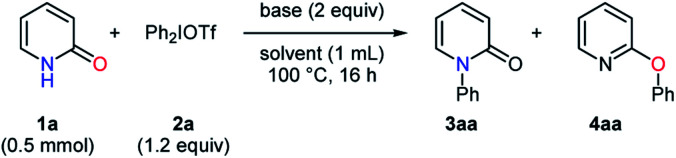				
Entry	Base	Solvent	**3aa** (%)	**4aa** (%)
1	DIPEA	Toluene	86	6
2	*n*-Bu_3_N	Toluene	85	4
3	Et_2_NPh	Toluene	88	6
4	Me_2_NPh	Toluene	77	3
5	MeNPh_2_	Toluene	30	7
6	Et_2_NPh	Dioxane	83	4
7	Et_2_NPh	DMA	73	8
8	Et_2_NPh	DMSO	43	4
9	Et_2_NPh	FPh	90(90)^b^	Trace(trace)^b^
10	Et_2_NPh	ClPh	85	6
11	DIPEA	FPh	81	4
12	*n*-Bu_3_N	FPh	75	6

a Reaction conditions: **1a** (0.5 mmol), Ph_2_IOTf (1.2 equiv.), base (2 equiv.), solvent (1 mL), 100 °C, 16 h.

b 85 °C.

Subsequently, the reaction conditions for the selective *O*-arylation were optimized at a higher temperature (Table 3). The influences of pyridine and related compounds^18^ were tested in toluene. The use of pyridine and 2,6-lutidine gave the *O*-phenylated product **4aa** in moderate yields with good selectivites, and 2,6-lutidine led to a slightly better result (entries 1–2). Pyrazine caused a significant decrease in yield, while isoquinoline provided almost the same result as pyridine (entries 3–4). In the presence of quinoline, **4aa** was obtained with a high selectivity despite a slightly lower yield as compared to 2,6-lutidine (entry 5). Then, the examination of solvents was conducted with quinoline. More polar solvents such as dioxane, DMA, and DMSO conduced to decreased yields of **4aa** (entries 6–8). Although fluorobenzene was not suitable for the *O*-phenylation of pyridin-2-one, chlorobenzene gave **4aa** in high yields with excellent selectivities especially at a higher temperature (entries 9–10).^19^

**Table 3:** Optimization for *O*-arylation^a^

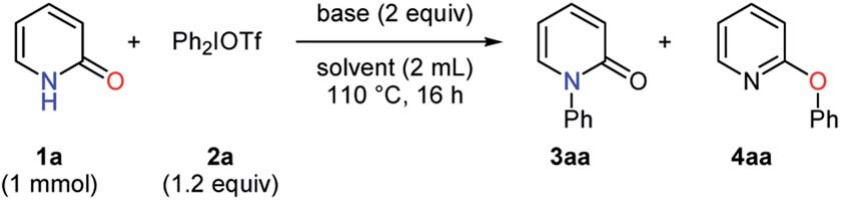				
Entry	Base	Solvent	**3aa** (%)	**4aa** (%)
1	Pyridine	Toluene	5	41
2	2,6-Lutidine	Toluene	6	51
3	Pyrazine	Toluene	2	18
4	Isoquinoline	Toluene	5	40
5	Quinoline	Toluene	2	47
6	Quinoline	Dioxane	1	18
7	Quinoline	DMA	9	12
8	Quinoline	DMSO	4	4
9	Quinoline	FPh	2	20
10	Quinoline	ClPh	Trace(trace)^b^	79(96)^b^

a Reaction conditions: **1a** (1 mmol), Ph_2_IOTf (1.2 equiv.), base (2 equiv.), solvent (2 mL), 110 °C, 16 h.

b 130 °C.

The influence of varying pyridin-2-ones and diaryliodonium salts on the selective *N*-arylation was studied (Table 4). The transformation of 5-methylpyridin-2-one (**1b**) proceeded smoothly to give the desired product in a high yield (**3ba**).^20^ Pyridin-2-ones with an electron-withdrawing group at the 5-position were converted with no problem, and an ester moiety proved to be tolerated under the conditions (**3ca–ea**). In the examination of 3-substituted substrates, electron-donating and -withdrawing groups gave no significant decrease in yield (**3fa–ha**).^21^ Whereas a high yield was observed in the *N*-phenylation of 4-chloropyridin-2-one, 6-chloropyridin-2-one afforded only the *N*-phenylated product in 33% yield (**3ia–ja**). On the other hand, pyridin-4-one was found to be a good reaction partner for this transformation (**3ka**). In addition, the investigation of diaryliodonium salts was carried out. A diaryliodonium triflate and tetrafluoroborate bearing an electron-donating group led to high yields of the *N*-arylated products (**3ab–ac**). Electron-withdrawing substituents such as fluoro, chloro, and ester groups caused no serious difficulty (**3ad–af**). In the presence of steric hindrance close to a reactive site, the desired product was obtained in 75% yield with a relatively lower selectivity (**3ag**). In most cases, quite high selectivities (>20 : 1) were observed except for the selective formation of **3ag**.

**Table 4:** Selective *N*-arylation of pyridin-2-ones with diaryliodonium salts

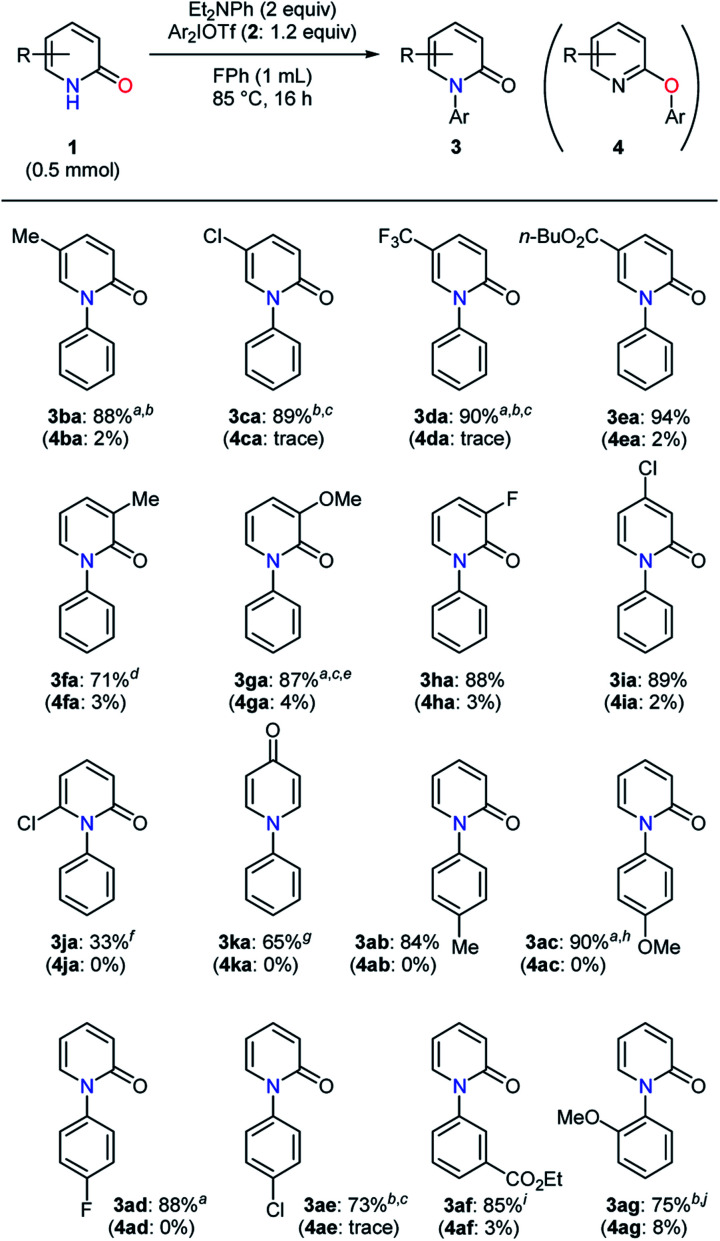

a 1 mmol scale.

b Et_2_NPh (2.5 equiv.).

c 100 °C.

d DIPEA (2.5 equiv.), Ph_2_IBF_4_ (1.2 equiv.).

e DIPEA (3 equiv.), 2-fluorotoluene.

f ClPh, 130 °C.

g Ph_2_IOTf (1 equiv.).

h (4-MeOPh)_2_IBF_4_ (1.2 equiv.).

i (3-EtO_2_CPh)_2_IBF_4_ (1.2 equiv.).

j (2-MeOPh)_2_IBF_4_ (1.2 equiv.).

The examination of pyridin-2-ones and diaryliodonium salts was conducted in the selective *O*-arylation (Table 5). Most of the 5-substituted substrates bearing an electron-donating or -withdrawing group gave high yields (**4ba–ca** and **4ea**), although a slight decrease in yield was observed in the presence of a trifluoromethyl group (**4da**). Even under the electronic and steric influences of substituents at the 3-position, the *O*-arylated products **4fa–ha** were also obtained uneventfully. The transformation of 6-methoxypyridin-2-one as well as 4-chloropyridin-2-one was carried out with favorable results (**4ia** and **4la**). Moreover, pyridin-4-one proved to be a suitable substrate for the selective *O*-arylation (**4ka**). Subsequently, a set of diaryliodonium triflates were investigated. Aryl moieties with a substituent such as alkyl, trifluoromethyl, and halogen groups were efficiently transferred to an oxygen atom of pyridin-2-one (**4ah–ai** and **4ad–ae**). Neither an ester group nor *ortho*-methyl group led to harmful effects, affording **4aj** and **4ak** in high yields with almost no side product. In all cases, the *O*-arylated products were formed with excellent selectivities (> 20 : 1).

**Table 5:** Selective *O*-arylation of pyridin-2-ones with diaryliodonium salts

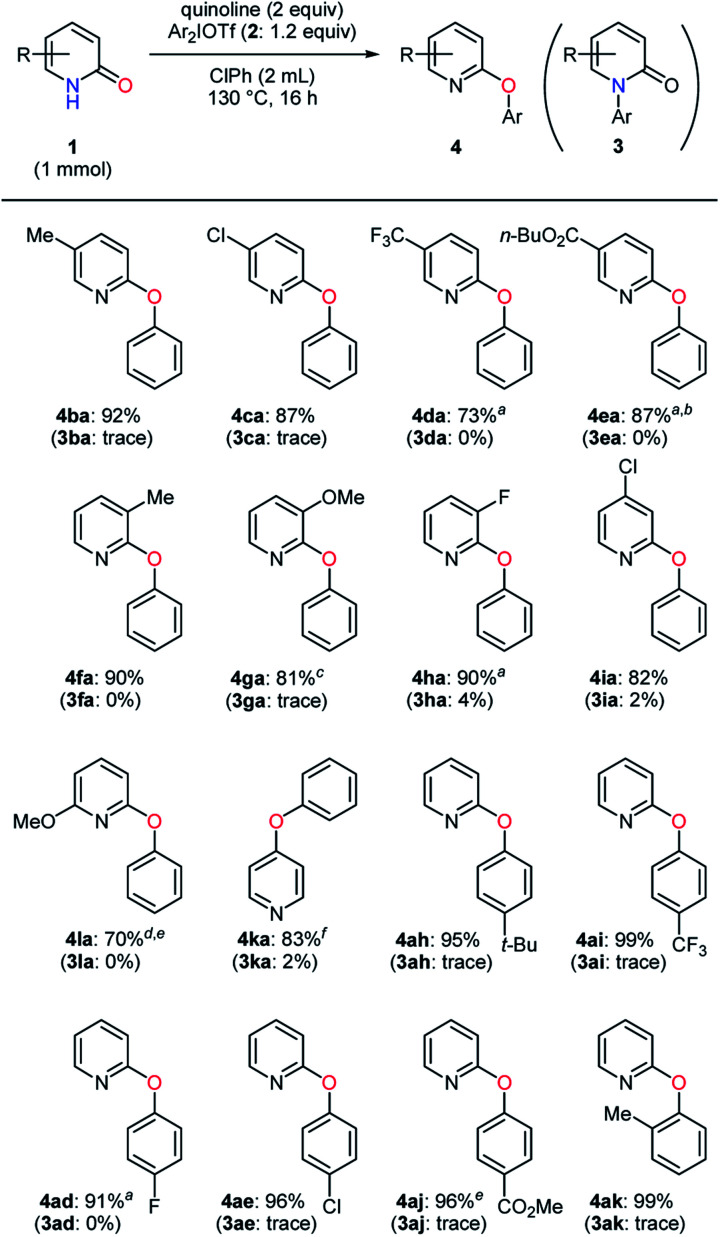

a 1,2-Cl_2_Ph, 140 °C.

b Ph_2_IOTf (1.5 equiv.).

c 8 h.

d Ph_2_IOTf (2.5 equiv.).

e 0.25 mmol scale.

f 1 h.

**Scheme 2:**
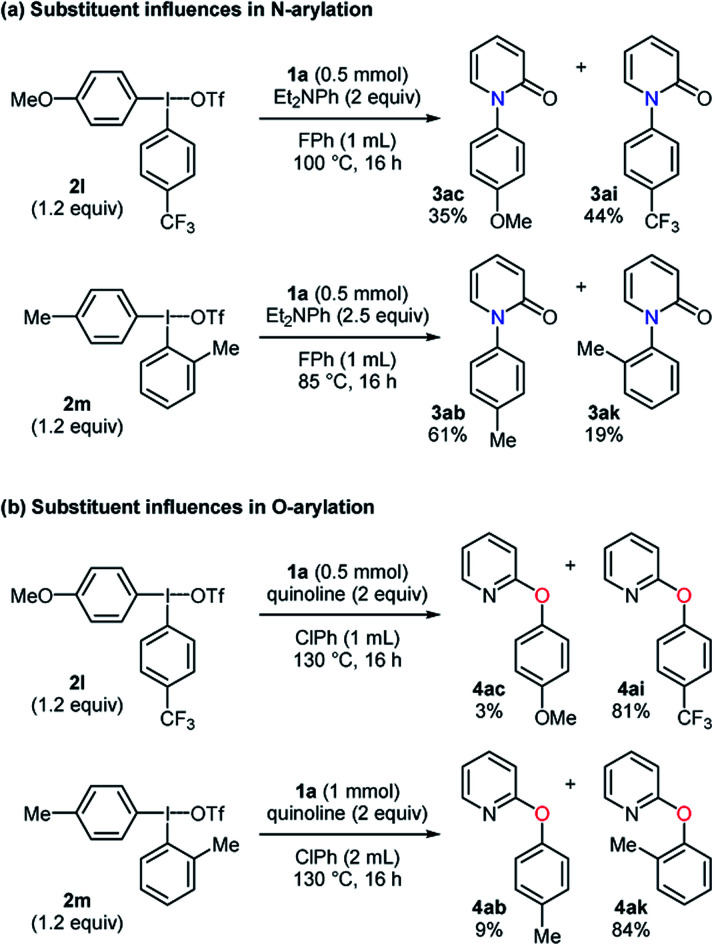
Substituent influences of diaryliodonium triflates on aryl group transfer preference in *N*-arylation and *O*-arylation.

**Scheme 3:**
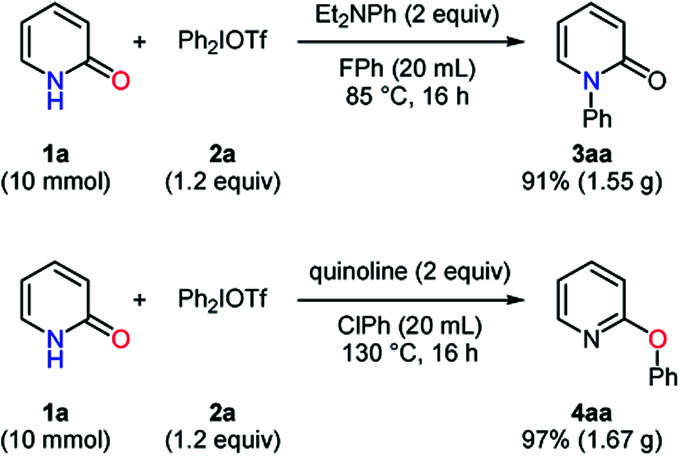
Examination of the scalability for *N*- and *O*-arylation.

To explore the influences of substituents on aryl group transfer preference, unsymmetrical diaryliodonium salts were tested in the selective *N*-arylation (Scheme 2a). When a diaryliodonium triflate with an electron-donating and -withdrawing group was employed, the *N*-arylated products **3ac** and **3ai** were obtained in 35% and 44% yields, respectively. In the examination of steric effects in an arylating agent, the diaryliodonium triflate **2m** favorably transferred 4-methylphenyl group to pyridin-2-one, providing a 3.2 : 1 ratio of **3ab** : **3ak**. While the *anti-ortho* effect was observed, the electronic effect was not pronounced unlike the reported *N*-arylation of amides.^22^ A similar investigation was conducted in the selective *O*-arylation (Scheme 2b). The use of the diaryliodonium triflate **2l** caused the selective formation of the CF_3_-containing product in a high yield with a 1 : 27 ratio of **4ac** : **4ai**. A preferential transfer of 2-methylphenyl group from the aryl source **2m** was observed to give **4ab** and **4ak** in 9% and 84% yields, respectively. These results had a similar tendency to those in the reported arylation of phenols.^23^ Besides, the scalability of these transformations was studied in a 10 mmol scale, and the desired products were obtained with high yields in both the selective *N*- and *O*-phenylation of pyridin-2-one (Scheme 3).

**Scheme 4:**
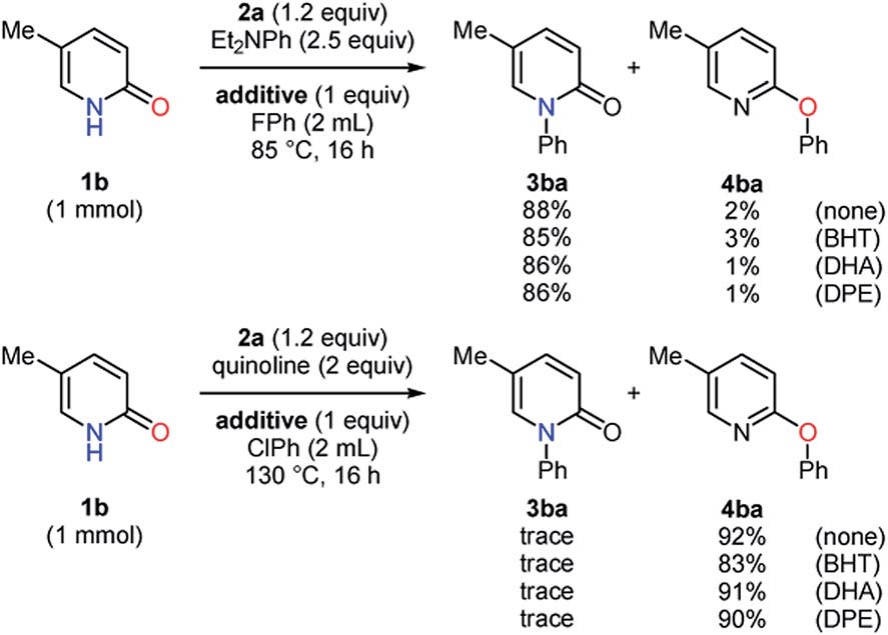
*N*- and *O*-arylation in the presence of radical scavengers.

**Scheme 5:**
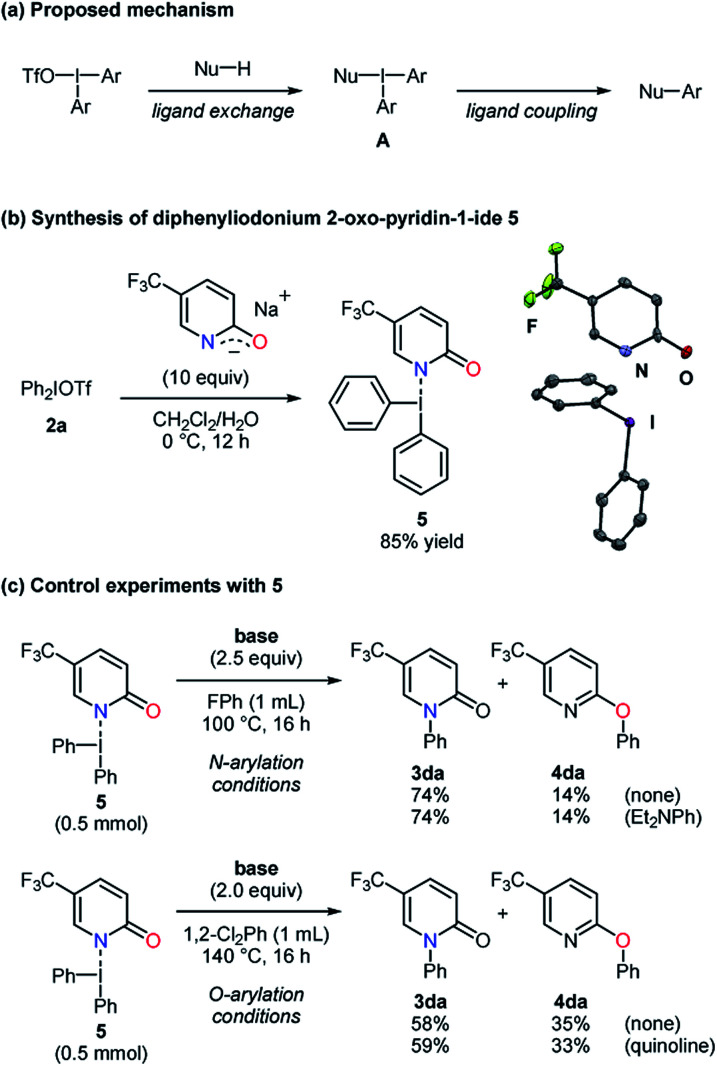
Synthesis of diaryliodonium salt **5** and control experiments.

To obtain further information on these methods, 2,6-di-*tert*-butyl-4-methylphenol (BHT), 9,10-dihydroanthracene (DHA), and 1,1-diphenylethylene (DPE) were employed as radical scavengers (Scheme 4). The *N*- and *O*-phenylation of the substrate **1b** smoothly proceeded with high selectivities even in the presence of these radical trapping reagents, which suggested that single electron transfer processes might not be included.^22,23^ Because the reaction mechanism for the carbon–heteroatom bond formation with diaryliodonium salts has been proposed on the basis of the T-shaped intermediate **A** in the literature (Scheme 5a),^16,17^ the ligand exchange at the iodine center of **2a** using the sodium salt of the pyridin-2-one **1d** was carried out to afford the iodonium salt **5** (Scheme 5b).^24^ According to X-ray analysis, the compound **5** basically possesses the T-shaped structure with the N–I bond length of 2.740 Å and the N–I–C bond angle of 168.93°. The carbon–oxygen distance of 1.286 Å is closer to the C=O bond length.^21^ The small deviation of the N–I–C bond angle from linearity and slightly longer C=O bond length might result from a partial electron delocalization in the amidate moiety. The iodonium salt **5** was subjected to the *N*-arylation conditions to give the *N*-phenylated product **3da** in a high yield with a good selectivity, and *N*,*N*-diethylaniline showed no significant influence (Scheme 5c). Under the *O*-arylation conditions, **3da** was obtained from **5** as a major product with a lower selectivity despite the presence or absence of quinoline. The interaction between the amidate moiety and iodine center could play a key role in the selective C–N bond formation, but the decreased selectivity could suggest more than one pathway.^25^ Meanwhile, the iodonium salt **5** might not be directly relevant to the selective *O*-arylation.

## Conclusions

In summary, a complementary set of the selective *N*- and *O*-arylation for pyridin-2-ones with diaryliodonium salts has been developed under metal-free conditions. While *N*-arylated products were obtained in high yields with high selectivities by using *N*,*N*-diethylaniline in fluorobenzene, the reaction conditions with quinoline in chlorobenzene led to the highly selective formation of *O*-arylated products in high yields. These methods were applicable to various substituted pyridin-2-ones as well as pyridin-4-one, and a series of diaryliodonium salts proved to be good reaction partners. Further studies on the detailed reaction mechanism including selectivity and application towards synthesis of bioactive compounds are ongoing in our laboratory.

## Conflicts of interest

There are no conflicts to declare.

## Supplementary Material

SC-011-D0SC02516J-s001

SC-011-D0SC02516J-s002
