# Supplementary material for: N- and O-arylation of pyridin-2-ones with diaryliodonium salts: base-dependent orthogonal selectivity under metal-free conditions
Source: Chem Sci. 2020 Jul 21;11(31):8295–300. doi: 10.1039/d0sc02516j (PMC8163315; doi:10.1039/d0sc02516j)
Supplement: SC-011-D0SC02516J-s001 [file SC-011-D0SC02516J-s001.pdf]

Electronic Supplementary Material (ESI) for Chemical Science.
This journal is © The Royal Society of Chemistry 2020

Electronic Supplementary Information

N- and O-Arylation of Pyridin-2-ones with Diaryliodonium Salts: Base-Dependent Orthogonal Selectivity under Metal-Free Conditions

Masami Kuriyama, Natsumi Hanazawa, Yusuke Abe, Kotone Katagiri, Shimpei Ono, Kosuke Yamamoto and Osamu Onomura**

Graduate School of Biomedical Sciences, Nagasaki University,
1-14 Bunkyo-machi, Nagasaki 852-8521, Japan

mkuriyam@nagasaki-u.ac.jp; onomura@nagasaki-u.ac.jp

Table of contents

1. General information S2
2. Experimental procedures and characterization data S2-S19
3. X-ray data of compounds **3ga**, **4ga** and **5** S20-S22
4. Comparison between the effects of FPh and ClPh S23
5. References S23
6. ^{1}H and ^{13}C NMR spectra S24-S64

General. All melting points are not corrected. IR spectra were expressed in cm^{-1} (Shimadzu IR Affinitiy-1). ^{1}H NMR spectra were taken at 500 or 400 MHz, and ^{13}C NMR spectra were taken at 125 or 100 MHz (Varian NMR System 500PS SN / JEOL JNM-AL400). Chemical shift values of ^{1}H NMR are expressed in ppm relative to internal or external TMS. Abbreviations are as follows: s, singlet; d, doublet; t, triplet; q, quartet; m, multiplet; br, broad. A double-focusing magnetic sector mass spectrometer was used for low- and high-resolution EI-MS and FAB-MS (JEOL JMS-700N). A reflectron time-of-flight mass spectrometer was applied to low-resolution DART-MS (JEOL JMS-T100TD). The coupling products were isolated by silica gel column chromatography, while the iodonium salts were purified by alumina column chromatography prior to use if necessary. All reactions were performed under an argon atmosphere unless otherwise specified. Commercially available chemicals were purchased from Sigma-Aldrich, Tokyo Chemical Industry Co., Ltd., and Fujifilm Wako Pure Chemical Corporation. These chemicals were used as received. The known compounds such as pyridones **1g**[1], **1l**[2], iodonium salts **2a-b**[3], **2c**[4], **2d-e**[3], **2f**[5], **2g**[6], **2h**[3], **2i**[7], **2j**[8], **2k**[7], and **2m**[3] were prepared as previously reported. As the authentic samples for identification and yield calculation with ^{1}H NMR analysis, **3ak**[9] and **4ab**[10] were prepared as previously reported.

5-(*n*-Butoxycarbonyl)pyridin-2(1*H*)-one (1e)

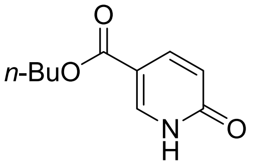

A mixture of 6-hydroxynicotinic acid (696 mg, 5 mmol) and a catalytic amount of concentrated sulfunic acid (10 mol%) in *n*-butanol (2.0 mL) was heated for 3 h at 140 °C. After complete conversion, the mixture was cooled to room temperature and water and saturated Na_2CO_3 were added until the mixture reached pH 11-12. The resulting mixture was extracted with AcOEt. Concentration and purification through silica gel column chromatography (hexane/benzene = 1/2) gave 880 mg of the product (4.5 mmol, 90%) as white solids of mp 128-130 °C. ^{1}H NMR (400 MHz, $CDCl_3$): δ 0.97 (3H, t, J = 7.3 Hz), 1.40-1.49 (2H, m), 1.67-1.74 (2H, m), 4.28 (2H, t, J = 6.3 Hz), 6.59 (1H, d, J = 9.3 Hz), 8.02 (1H, dd, J = 2.4, 9.3 Hz), 8.21 (1H, d, J = 2.4 Hz), 12.9 (1H, brs). ^{13}C NMR (100 MHz, $CDCl_3$): δ 13.5 (CH_3), 19.0 (CH_2), 30.5 (CH_2), 64.8 (CH_2), 111.4 (C), 119.4 (CH), 139.7 (CH), 141.1 (CH), 164.1 (C), 165.8 (C). IR (ATR): 780, 1220, 1650, 1720, 2960 cm^{-1}. HRMS (EI) *m/z* (M^+) Calcd for $C_{10}H_{13}NO_3$: 195.0895. Found: 195.0892.

(4-(Trifluoromethyl)phenyl)(4-methoxyphenyl)iodonium triflate[3] (2l)

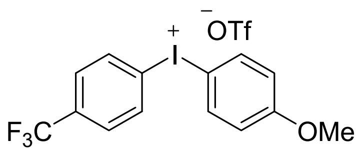

MCPBA (82% active oxidant, 1.16 g, 5.5 mmol) and 1-iodo-4-(trifluoromethyl)benzene (1.36 g, 5 mmol) were dissolved in CH_2Cl_2 (25 mL), and TfOH (1.50 g, 10.0 mmol) was added at room temperature. After stirring at room temperature for 10 min, anisole (541 mg, 5.0 mmol) in CH_2Cl_2 (5 mL) was slowly added at 0 °C. The reaction mixture was stirred at 0 °C for 10 min and concentrated in vacuo at 0 °C. Et_2O (20 mL) was added, and then the mixture was stirred at room temperature for 10 min to precipitate off-white solids. After the flask was stored at 0 °C for 30 min, the solids were filtered off, washed with cold Et_2O, and dried under vacuum to give 2.07 g of the product (3.92 mmol, 78 %) as pale yellow solids of mp 151-152 °C. ^{1}H NMR (500 MHz, DMSO-d_6): δ 3.81 (3H, s), 7.10 (2H, d, J = 9.1 Hz), 7.91 (2H, d, J = 8.3 Hz), 8.22 (2H, d, J = 9.1 Hz), 8.39 (2H, d, J = 8.3 Hz). ^{13}C NMR (100 MHz, DMSO-d_6): δ 55.8 (CH_3), 105.6 (C), 117.8 (CH), 120.9 (q, J = 321.9 Hz, C), 121.3 (C), 123.6 (q, J = 272.3 Hz, C), 128.4 (q, J = 3.3 Hz, CH), 131.9 (q, J =32.3 Hz, C), 135.8 (CH), 137.7 (CH), 162.4 (C). ^{19}F NMR (470 MHz, DMSO-d_6): δ −61.7 (3F), −77.8 (3F). IR (ATR): 1020, 1070, 1140, 1170, 1240 cm^{-1}. HRMS (FAB) m/z ([M−TfO]$^+$) Calcd for $C_{14}H_{11}F_3IO$: 378.9807. Found: 378.9807.

General procedure for N-arylation of pyridin-2-ones with diaryliodonium salts. A screw cap test tube was charged with pyridin-2-one (**1a**) (47.5 mg, 0.5 mmol) and diphenyliodonium triflate (**2a**) (258 mg, 0.6 mmol). Then, the tube was evacuated and back-filled with argon. After fluorobenzene (1 mL) and diethylaniline (149 mg, 1.0 mmol) were added, a septum cap was replaced with a screw cap. The reaction mixture was stirred at 85 °C for 16 h. The mixture was cooled to room temperature and saturated Na_2CO_3 solution was added. The resulting mixture was extracted with AcOEt. The combined organic layers were dried over Na_2SO_4. Concentration and purification through silica gel column chromatography gave the desired product **3aa**.

1-Phenylpyridin-2(1*H*)-one[11] (3aa)

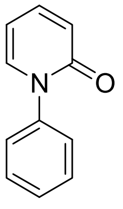

Silica gel column chromatography (hexane/EtOAc = 1/2) gave 77 mg of the product (0.45 mmol,

90%) as white solids of mp 126-128 °C. ^{1}H NMR (400 MHz, $CDCl_3$): δ 6.24 (1H, dt, *J* = 1.3, 6.8 Hz), 6.67 (1H, d, *J* = 9.2 Hz), 7.34 (1H, dd, *J* = 1.8, 6.8 Hz), 7.38-7.45 (4H, m), 7.48-7.52 (2H, m). ^{13}C NMR (100 MHz, $CDCl_3$): δ 105.6 (CH), 121.4 (CH), 126.2 (CH), 128.1 (CH), 128.9 (CH), 137.7 (CH), 139.6 (CH), 140.6 (C), 162.0 (C). IR (ATR): 690, 760, 1530, 1580, 1650 cm^{-1}. EIMS *m/z*: 171 (M^+).

5-Methyl-1-phenylpyridin-2(1*H*)-one[12] (3ba)

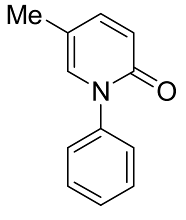

This reaction was conducted on 1 mmol scale. Silica gel column chromatography (hexane/EtOAc = 1/2) gave 163 mg of the product (0.88 mmol, 88%) as white solids of mp 107-109 °C. ^{1}H NMR (500 MHz, $CDCl_3$): δ 2.11 (3H, s), 6.61 (1H, d, *J* = 9.3 Hz), 7.11 (1H, s), 7.25-7.28 (1H, m), 7.37-7.42 (3H, m), 7.47-7.50 (2H, m). ^{13}C NMR (100 MHz, $CDCl_3$): δ 16.9 (CH_3), 114.8 (C), 121.4 (CH), 126.6 (CH), 128.3 (CH), 129.3 (CH), 135.3 (CH), 141.1 (C), 142.6 (CH), 161.8 (C). IR (ATR): 830, 1140, 1490, 1610, 1670 cm^{-1}. EIMS *m/z*: 185 (M^+).

5-Chloro-1-phenylpyridin-2(1*H*)-one[13] (3ca)

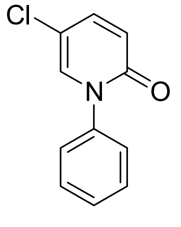

Silica gel column chromatography (hexane/EtOAc = 3/1) gave 91 mg of the product (0.44 mmol, 89%) as pale beige solids of mp 83-84 °C. ^{1}H NMR (400 MHz, $CDCl_3$): δ 6.64 (1H, d, *J* = 9.8 Hz), 7.34-7.53 (7H, m). ^{13}C NMR (100 MHz, $CDCl_3$): δ 112.5 (C), 122.7 (CH), 126.4 (CH), 128.9 (CH), 129.5 (CH), 135.5 (CH), 140.2 (C), 140.9 (CH), 160.9 (C). IR (ATR): 690, 820, 1090, 1490, 1670 cm^{-1}. EIMS *m/z*: 205 (M^+).

1-Phenyl-5-(trifluoromethyl)pyridin-2(1*H*)-one (3da)

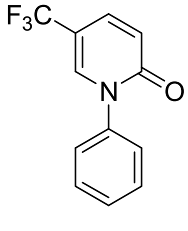

This reaction was conducted on 1 mmol scale. Silica gel column chromatography (hexane/EtOAc = 5/1) gave 215 mg of the product (0.90 mmol, 90%) as yellow solids of mp 57-58 °C. ^{1}H NMR (500 MHz, $CDCl_3$): δ 6.73 (1H, d, J = 9.8 Hz), 7.38 (2H, d, J = 7.3 Hz), 7.46-7.49 (1H, m), 7.52-7.55 (3H, m), 7.75 (1H, s). ^{13}C NMR (125 MHz, $CDCl_3$): δ 109.5 (q, J = 35.3 Hz, C), 122.2 (CH), 123.1 (q, J = 269.0 Hz, C), 126.2 (CH), 129.0 (CH), 129.4 (CH), 135.2 (CH), 137.5 (q, J = 4.8 Hz, CH), 139.8 (C), 161.3 (C). IR (ATR): 690, 750, 1090, 1590, 1680 cm^{-1}. HRMS (EI): *m/z* (M^+) Calcd for $C_{12}H_8F_3NO$: 239.0558. Found:239.0559.

5-Butoxycarbonyl-1-phenylpyridin-2(1*H*)-one (3ea)

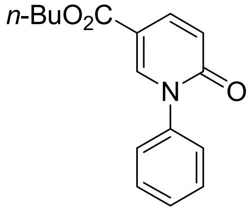

Silica gel column chromatography (hexane/EtOAc = 3/1) gave 128 mg of the product (0.47 mmol, 94%) as yellow solids of mp 63-64 °C. ^{1}H NMR (400 MHz, $CDCl_3$): δ 0.96 (3H, t, J = 7.3 Hz), 1.38-1.48 (2H, m), 1.67-1.74 (2H, m), 4.28 (2H, t, J = 6.8 Hz), 6.64 (1H, d, J = 9.8 Hz), 7.39 (2H, d, J = 7.8 Hz), 7.46-7.55 (3H, m), 7.93 (1H, dd, J = 2.0, 9.8 Hz), 8.22 (1H, d, J = 2.4 Hz). ^{13}C NMR (100 MHz, $CDCl_3$): δ 13.6 (CH_3), 19.1 (CH_2), 30.6 (CH_2), 65.0 (CH_2), 110.2 (C), 120.6 (CH), 126.5 (CH), 129.1 (CH), 129.6 (CH), 139.0 (CH), 140.2 (C), 143.2 (CH), 162.3 (C), 164.3 (C). IR (ATR): 700, 800, 1110, 1650, 1710 cm^{-1}. HRMS (EI): *m/z* (M^+) Calcd for $C_{16}H_{17}NO_3$: 271.1208. Found: 271.1208.

3-Methyl-1-phenylpyridin-2(1*H*)-one[11] (3fa)

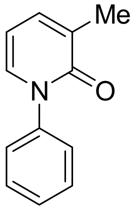

Silica gel column chromatography (hexane/EtOAc = 2/1) gave 66 mg of the product (0.36 mmol, 71%) as pale yellow solids of mp 118-120 °C. ^{1}H NMR (400 MHz, $CDCl_3$): δ 2.19 (3H, s), 6.17 (1H, t, J = 6.8 Hz), 7.23 (1H, d, J = 6.8 Hz), 7.27 (1H, d, J = 6.8 Hz), 7.37-7.43 (3H, m), 7.46-7.50 (2H, m). ^{13}C NMR (100 MHz, $CDCl_3$): δ 17.2 (CH_3), 105.5 (CH), 126.6 (CH), 128.2 (CH), 129.2 (CH), 130.8 (C), 135.4 (CH), 136.9 (CH), 141.3 (C), 162.8 (C). IR (ATR): 700, 1070, 1490, 1590, 1650 cm^{-1}. EIMS *m/z*: 185 (M^+).

3-Methoxy-1-phenylpyridin-2(1*H*)-one (3ga)

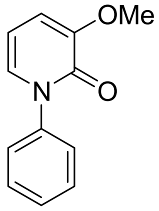

This reaction was conducted on 1 mmol scale. Silica gel column chromatography (EtOAc) gave 175 mg of the product (0.87 mmol, 87%) as white solids of mp 134-136 °C. ^{1}H NMR (400 MHz, $CDCl_3$): δ 3.86 (3H, s), 6.18 (1H, t, *J* = 7.3 Hz), 6.67 (1H, dd, *J* = 1.5, 7.3 Hz), 6.98 (1H, dd, *J* = 1.5, 6.8 Hz), 7.39-7.43 (3H, m), 7.47-7.51 (2H, m). ^{13}C NMR (100 MHz, $CDCl_3$): δ 55.9 (CH_3), 104.8 (CH), 121.2 (CH), 126.5 (CH), 128.4 (CH), 129.0 (CH), 129.3 (CH), 140.9 (C), 150.5 (C), 158.0 (C). IR (ATR): 710, 1110, 1240, 1580, 1650 cm^{-1}. HRMS (EI): *m/z* (M^+) Calcd for $C_{12}H_{11}NO_2$: 201.0790. Found: 201.0790.

3-Fluoro-1-phenylpyridin-2(1*H*)-one (3ha)

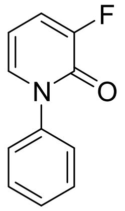

Silica gel column chromatography (hexane/EtOAc = 2/1) gave 83 mg of the product (0.44 mmol, 88%) as pink solids of mp 136-137 °C. ^{1}H NMR (400 MHz, $CDCl_3$): δ 6.16-6.20 (1H, m), 7.16-7.19 (2H, m), 7.40 (2H, d, *J* = 7.8 Hz), 7.45 (1H, t, *J* = 7.8 Hz), 7.52 (2H, t, *J* = 7.8 Hz). ^{13}C NMR (100 MHz, $CDCl_3$): δ 103.7 (d, *J* = 5.8 Hz, CH), 120.2 (d, *J* = 17.4 Hz, CH), 126.4 (CH), 128.9 (CH), 129.5 (CH), 133.3 (d, *J* = 5.0 Hz, CH), 140.0 (C), 152.8 (d, *J* = 250.8 Hz, C), 156.3 (d, *J* = 25.7 Hz, C). IR (ATR): 750, 1110, 1490, 1610, 1660 cm^{-1}. HRMS (EI): *m/z* (M^+) Calcd for $C_{11}H_8FNO$: 189.0590. Found:189.0589.

4-Chloro-1-phenylpyridin-2(1*H*)-one (3ia)

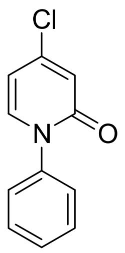

Silica gel column chromatography (hexane/EtOAc = 1/1) gave 91 mg of the product (0.44 mmol,

89%) as white solids of mp 131-132 °C. ^{1}H NMR (500 MHz, $CDCl_3$): δ 6.28 (1H, dd, *J* = 2.2, 7.3 Hz), 6.71 (1H, d, *J* = 2.2 Hz), 7.29 (1H, d, *J* = 7.3 Hz), 7.35 (2H, d, *J* = 7.3 Hz), 7.44 (1H, t, *J* = 7.3 Hz), 7.50 (2H, t, *J* = 7.3 Hz). ^{13}C NMR (125 MHz, $CDCl_3$): δ 107.7 (CH), 119.9 (CH), 126.2 (CH), 128.6 (CH), 129.3 (CH), 137.9 (CH), 139.8 (C), 146.9 (C), 161.0 (C). IR (ATR): 690, 840, 1060, 1520, 1660 cm^{-1}. HRMS (EI) *m/z* Calcd for $C_{11}H_8{}^{35}ClNO$: 205.0294. Found: 205.0294.

6-Chloro-1-phenylpyridin-2(1*H*)-one (3ja)

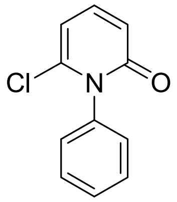

Silica gel column chromatography (hexane/benzene = 5/1) gave 34 mg of the product (0.17 mmol, 33%) yellow solids of mp 78-80 °C. ^{1}H NMR (400 MHz, $CDCl_3$): δ 6.74 (1H, d, *J* = 7.8 Hz), 7.04 (1H, d, *J* = 7.8 Hz), 7.15 (2H, d, *J* = 7.8 Hz), 7.23 (1H, t, *J* = 7.8 Hz), 7.41 (2H, t, *J* = 7.8 Hz), 7.62 (1H, t, *J* = 7.8 Hz). ^{13}C NMR (100 MHz, $CDCl_3$): δ 109.1 (CH), 118.5 (CH), 121.0 (CH), 125.1 (CH), 129.8 (CH), 141.5 (CH), 149.2 (C), 153.7 (C), 163.2 (C). IR (ATR): 690, 790, 1140, 1200, 1580 cm^{-1}. HRMS (EI): *m/z* (M^+) Calcd for $C_{11}H_8{}^{35}ClNO$: 205.0294. Found: 205.0295.

1-Phenylpyridin-4(1*H*)-one (3ka)

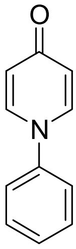

Silica gel column chromatography (hexane/EtOAc = 1/5) gave 56 mg of the product (0.33 mmol, 65%) as white solids of mp 125-127 °C. ^{1}H NMR (400 MHz, $CDCl_3$): δ 6.51 (2H, d, *J* = 7.8 Hz), 7.36 (2H, d, *J* = 7.8 Hz), 7.46 (1H, t, *J* = 7.8 Hz), 7.54 (2H, t, *J* = 7.8 Hz), 7.61 (2H, d, *J* = 7.8 Hz). ^{13}C NMR (100 MHz, $CDCl_3$): δ 118.7 (CH), 122.8 (CH), 128.7 (CH), 130.3 (CH), 139.6 (CH), 143.1 (C), 178.9 (C). IR (ATR): 690, 860, 1030, 1490, 1630 cm^{-1}. HRMS (EI): *m/z* (M^+) Calcd for $C_{11}H_9NO$: 171.0684. Found: 171.0684.

1-(4-Tolyl)pyridin-2(1*H*)-one[14] (3ab)

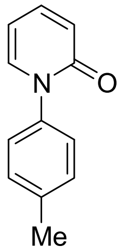

Silica gel column chromatography (hexane/EtOAc = 1/2) gave 78 mg of the product (0.42 mmol, 84%) as white solids of mp 135-137 °C. ^{1}H NMR (500 MHz, $CDCl_3$): δ 2.40 (3H, s), 6.22 (1H, t, *J* = 6.9 Hz), 6.65 (1H, d, *J* = 9.3 Hz), 7.25-7.33 (5H, m), 7.38 (1H, t, *J* = 7.1 Hz). ^{13}C NMR (100 MHz, $CDCl_3$): δ 21.0 (CH_3), 105.7 (CH), 121.8 (CH), 126.3 (CH), 129.9 (CH), 138.2 (CH), 138.5 (C), 139.8 (CH), 162.6 (C). IR (ATR): 760, 1150, 1510, 1580, 1650 cm^{-1}. EIMS *m/z*: 185 (M^+).

1-(4-Methoxyphenyl)pyridin-2(1*H*)-one[11] (3ac)

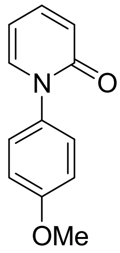

This reaction was conducted on 1 mmol scale. Silica gel column chromatography (hexane/EtOAc = 1/5) gave 181 mg of the product (0.90 mmol, 90%) as yellow solids of mp 101-103 °C. ^{1}H NMR (500 MHz, $CDCl_3$): δ 3.85 (3H, s), 6.22 (1H, t, *J* = 6.6 Hz), 6.65 (1H, d, *J* = 9.3 Hz), 6.99 (2H, d, *J* = 8.8 Hz), 7.29-7.33 (3H, m), 7.36-7.40 (1H, m). ^{13}C NMR (100 MHz, $CDCl_3$): δ 55.5 (CH_3), 105.7 (CH), 114.5 (CH), 121.8 (CH), 127.6 (CH), 133.8 (C), 138.3 (CH), 139.8 (CH), 159.4 (C), 162.7 (C). IR (ATR): 760, 1030, 1240, 1510, 1660 cm^{-1}. EIMS *m/z*: 201 (M^+).

1-(4-Fluorophenyl)pyridin-2(1*H*)-one (3ad)

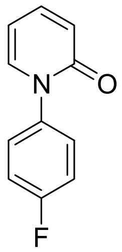

This reaction was conducted on 1 mmol scale. Silica gel column chromatography (hexane/EtOAc

= 1/2) gave 166 mg of the product (0.88 mmol, 88%) as pale yellow solids of mp 142-143 °C. ^{1}H NMR (400 MHz, $CDCl_3$): δ 6.25 (1H, t, *J* = 6.8 Hz), 6.67 (1H, d, *J* = 8.8 Hz), 7.18 (2H, t, *J* = 8.8 Hz), 7.31 (1H, dd, *J* = 2.0, 6.8 Hz), 7.36-7.43 (3H, m). ^{13}C NMR (100 MHz, $CDCl_3$): δ 106.0 (CH), 116.3 (d, *J* = 23.2 Hz, CH), 121.9 (CH), 128.4 (d, *J* = 9.1 Hz, CH), 136.9 (d, *J* = 2.5 Hz, C), 137.9 (CH), 140.0 (CH), 162.2 (d, *J* = 248.3 Hz, C), 162.4 (C). IR (ATR): 760, 1000, 1150, 1220, 1650 cm^{-1}. HRMS (EI): *m/z* (M^+) Calcd for $C_{11}H_8FNO$: 189.0590. Found:189.0590.

1-(4-Chlorophenyl)pyridin-2(1*H*)-one[11] (3ae)

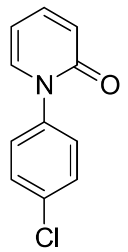

Silica gel column chromatography (hexane/EtOAc = 1/2) gave 75 mg of the product (0.36 mmol, 73%) as white solids of mp 133-134 °C. ^{1}H NMR (400 MHz, $CDCl_3$): δ 6.26 (1H, dt, *J* = 1.2, 6.8 Hz), 6.65-6.68 (1H, m), 7.30 (1H, dd, *J* = 2.0, 6.8 Hz), 7.33-7.36 (2H, m), 7.39-7.43 (1H, m), 7.45-7.49 (2H, m). ^{13}C NMR (100 MHz, $CDCl_3$): δ 106.2 (CH), 122.0 (CH), 127.9 (CH), 129.5 (CH), 134.4 (C), 137.6 (CH), 139.3 (C), 140.1 (CH), 162.3 (C). IR (ATR): 760, 840, 1080, 1140, 1650 cm^{-1}. EIMS *m/z*: 205 (M^+).

1-(3-Ethoxycarbonylphenyl)pyridin-2(1*H*)-one (3af)

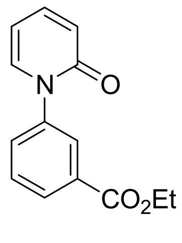

Silica gel column chromatography (hexane/EtOAc = 1/1) gave 103 mg of the product (0.42 mmol, 85%) as off-white solids of mp 91-92 °C. ^{1}H NMR (400 MHz, $CDCl_3$): δ 1.40 (3H, t, *J* = 7.3 Hz), 4.40 (2H, q, *J* = 7.3 Hz), 6.28 (1H, t, *J* = 6.8 Hz), 6.68 (1H, d, *J* = 9.3 Hz), 7.34-7.36 (1H, m), 7.40-7.45 (1H, m), 7.58 (1H, t, *J* = 7.8 Hz), 7.63-7.65 (1H, m), 8.05 (1H, s), 8.12 (1H, d, *J* = 7.8 Hz). ^{13}C NMR (100 MHz, $CDCl_3$): δ 14.2 (CH_3), 61.3 (CH_2), 106.2 (CH), 122.0 (CH), 127.6 (CH), 129.4 (CH), 129.6 (CH), 131.1 (CH), 132.0 (C), 137.7 (CH), 140.1 (CH), 141.0 (C), 162.3 (C), 165.5 (C). IR (ATR): 760, 1240, 1590, 1650, 1720 cm^{-1}. HRMS (EI): *m/z* (M^+) Calcd for $C_{14}H_{13}NO_3$: 243.0895. Found: 243.0894.

Ethyl 3-(pyridin-2-yloxy)benzoate[15] (4af)

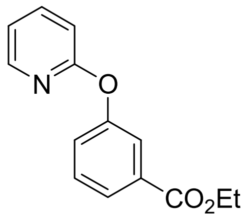

Silica gel column chromatography (hexane/Et_2O = 5/1, 2/1) gave 7 mg of the product (0.03 mmol, 3%) as a colorless oil. ^{1}H NMR (400 MHz, $CDCl_3$): δ 1.38 (3H, t, *J* = 7.3 Hz), 4.37 (2H, q, *J* = 7.3 Hz), 6.96 (1H, d, *J* = 8.3 Hz), 7.03 (1H, dd, *J* = 4.9, 6.3 Hz), 7.34-7.36 (1H, m), 7.48 (1H, t, *J* = 7.8 Hz), 7.70-7.74 (1H, m), 7.81-7.82 (1H, m), 7.90 (1H, d, *J* = 7.8 Hz), 8.19 (1H, dd, *J* = 1.5, 4.9 Hz). ^{13}C NMR (100 MHz, $CDCl_3$): δ 14.2 (CH_3), 61.1 (CH_2), 111.7 (CH), 118.8 (CH), 122.3 (CH), 125.8 (CH), 125.9 (CH), 129.6 (CH), 132.2 (C), 139.6 (CH), 147.7 (CH), 154.1 (C), 163.4 (C), 166.0 (C). IR (ATR): 750, 1100, 1260, 1590, 1710 cm^{-1}. DART-MS *m/z*: 244 $[M+H]^+$.

1-(2-Methoxyphenyl)pyridin-2(1*H*)-one[16] (3ag)

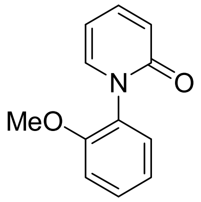

Silica gel column chromatography (hexane/EtOAc = 1/1) gave 75 mg of the product (0.37 mmol, 75%) as brown solids of mp 128-130 °C. ^{1}H NMR (400 MHz, $CDCl_3$): δ 3.82 (3H, s), 6.20 (1H, t, *J* = 6.8 Hz), 6.66 (1H, d, *J* = 9.3 Hz), 7.04-7.08 (2H, m), 7.24 (1H, dd, *J* = 2.0, 6.8 Hz), 7.26-7.28 (1H, m), 7.38-7.43 (2H, m). ^{13}C NMR (100 MHz, $CDCl_3$): δ 55.8 (CH_3), 105.3 (CH), 112.4 (CH), 120.9 (CH), 121.8 (CH), 128.5 (CH), 129.7 (C), 130.3 (CH), 139.0 (CH), 139.9 (CH), 154.3 (C), 162.4 (C). IR (ATR): 760, 1110, 1500, 1580, 1660 cm^{-1}. EIMS *m/z*: 201 (M^+).

2-(2-Methoxyphenoxy)pyridine[17] (4ag)

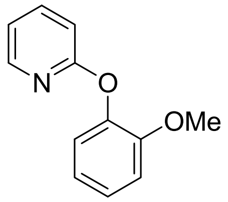

Silica gel column chromatography (hexane/Et_2O = 4/1) gave 8 mg of the product (0.04 mmol, 8%) as white solids of mp 93-94 °C. ^{1}H NMR (400 MHz, $CDCl_3$): δ 3.77 (3H, s), 6.91-7.03 (4H, m), 7.14-7.16 (1H, m), 7.19-7.23 (1H, m), 7.64-7.69 (1H, m), 8.15 (1H, d, *J* = 3.4 Hz). ^{13}C NMR

(100 MHz, $CDCl_3$): 55.8 (CH_3), 110.6 (CH), 112.8 (CH), 118.0 (CH), 121.1 (CH), 123.1 (CH), 126.0 (CH), 139.2 (CH), 142.6 (C), 147.6 (CH), 151.8 (C), 163.8 (C). IR (ATR): 750, 1020, 1110, 1240, 1270 cm^{-1}. DART-MS *m/z*: 202 $[M+H]^+$.

1-(4-(Trifluoromethyl)phenyl)pyridin-2(1*H*)-one (3ai)

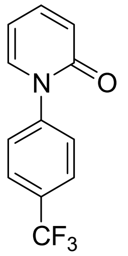

Silica gel column chromatography (hexane/EtOAc = 1/2) gave 53 mg of the product (0.22 mmol, 44%) as white solids of mp 167-169 °C. ^{1}H NMR (400 MHz, $CDCl_3$): δ 6.28-6.31 (1H, m), 6.69 (1H, d, J = 9.3 Hz), 7.32 (1H, dd, J = 1.5, 6.8 Hz), 7.41-7.46 (1H, m), 7.55 (2H, d, J = 8.3 Hz), 7.78 (2H, d, J = 8.3 Hz). ^{13}C NMR (125 MHz, $CDCl_3$): δ 106.4 (CH), 122.1 (CH), 123.6 (q, J = 271.8 Hz, C), 126.5 (q, J = 3.8 Hz, CH), 127.1 (CH), 130.6 (q, J = 33.4 Hz, C), 137.1 (CH), 140.2 (CH), 143.7 (C), 162.0 (C). IR (ATR): 760, 1070, 1140, 1580, 1650 cm^{-1}. HRMS (EI): *m/z* (M^+) Calcd for $C_{12}H_8F_3NO$:239.0558. Found: 239.0557.

General procedure for O-arylation of pyridin-2-ones with diaryliodonium triflates. A screw cap test tube was charged with pyridin-2-one (**1a**) (95 mg, 1.0 mmol) and diphenyliodonium triflate (**2a**) (516 mg, 1.2 mmol). Then, the tube was evacuated and back-filled with argon. After chlorobenzene (2 mL) and quinoline (258 mg, 2.0 mmol) was added, a septum cap was replaced with a screw cap. The reaction mixture was stirred at 130 °C for 16 h. The mixture was cooled to room temperature and saturated Na_2CO_3 solution was added. The resulting mixture was extracted with AcOEt. The combined organic layers were dried over Na_2SO_4. Concentration and purification through silica gel column chromatography gave the desired product **4aa**.

2-Phenoxypyridine[18] (4aa)

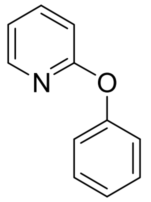

Silica gel column chromatography (hexane/EtOAc = 16/1) gave 164 mg of the product (0.96 mmol, 96%) as a colorless oil. ^{1}H NMR (400 MHz, $CDCl_3$): δ 6.91 (1H, d, J = 8.3 Hz), 6.98-7.01

(1H, m), 7.13-7.16 (2H, m), 7.19-7.22 (1H, m), 7.39-7.43 (2H, m), 7.66-7.71 (1H, m), 8.21 (1H, dd, J = 1.0, 4.9 Hz). ^{13}C NMR (100 MHz, $CDCl_3$): δ 111.4 (CH), 118.3 (CH), 121.0 (CH), 124.5 (CH), 129.5 (CH), 139.3 (CH), 147.6 (CH), 154.1 (C), 163.6 (C). IR (ATR): 690, 750, 1240, 1590, 3060 cm^{-1}. EIMS m/z: 171 (M^+).

5-Methyl-2-phenoxypyridine (4ba)

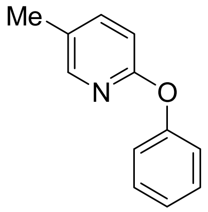

Silica gel column chromatography (hexane/EtOAc = 18/1) gave 170 mg of the product (0.92 mmol, 92%) as a yellow oil. ^{1}H NMR (500 MHz, $CDCl_3$): δ 2.28 (3H, s), 6.81 (1H, d, J = 8.3 Hz), 7.11 (2H, d, J = 7.6 Hz), 7.17 (1H, t, J = 7.6 Hz), 7.38 (2H, t, J = 7.6 Hz), 7.49 (1H, dd, J = 2.0, 8.3 Hz), 8.02 (1H, d, J = 2.0 Hz). ^{13}C NMR (100 MHz, $CDCl_3$): δ 17.4 (CH_3), 111.1 (CH), 120.8 (CH), 124.3 (CH), 127.8 (C), 129.7 (CH), 140.3 (CH), 147.5 (CH), 154.8 (C), 162.0 (C). IR (ATR): 690, 740, 1020, 1200, 1240, 1590, 3040 cm^{-1}. HRMS (EI) m/z Calcd for $C_{12}H_{11}NO$ (M^+): 185.0841. Found: 185.0837.

5-Chloro-2-phenoxypyridine (4ca)

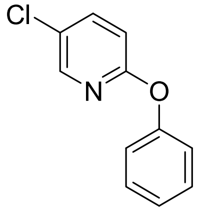

Silica gel column chromatography (hexane/benzene = 100/1) gave 179 mg of the product (0.87 mmol, 87%) as a colorless oil. ^{1}H NMR (400 MHz, $CDCl_3$): δ 6.88 (1H, d, J = 8.3 Hz), 7.12-7.14 (2H, m), 7.23 (1H, t, J = 7.8 Hz), 7.39-7.43 (2H, m), 7.64 (1H, dd, J = 2.9, 8.3 Hz), 8.13 (1H, d, J = 2.9 Hz). ^{13}C NMR (100 MHz, $CDCl_3$): δ 112.4 (CH), 121.1 (CH), 125.0 (CH), 125.8 (C), 129.7 (CH), 139.2 (CH), 146.1 (CH), 153.9 (C), 162.2 (C). IR (ATR): 690, 760, 1110, 1240, 1580, 3070 cm^{-1}. HRMS (EI) m/z (M^+) Calcd for $C_{11}H_8{}^{35}ClNO$: 205.0294; Found: 205.0296.

5-(Trifluoromethyl)-2-phenoxypyridine (4da)

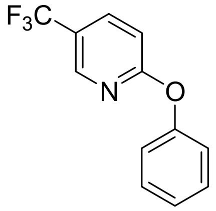

Silica gel column chromatography (hexane/EtOAc = 50/1) gave 175 mg of the product (0.73 mmol, 73%) as a colorless oil. ^{1}H NMR (400 MHz, $CDCl_3$): δ 7.01 (1H, d, J = 8.8 Hz), 7.15-7.17 (2H, m), 7.25-7.29 (1H, m), 7.44 (2H, t, J = 7.8 Hz), 7.90 (1H, dd, J = 2.4, 8.8 Hz), 8.45 (1H, s). ^{13}C NMR (100 MHz, $CDCl_3$): δ 111.3 (CH), 121.5 (CH), 121.5 (q, J = 33.9 Hz, C), 123.7 (q, J = 270.6 Hz, C), 125.5 (CH), 129.8 (CH), 136.6 (q, J = 3.3 Hz, CH), 145.5 (q, J = 4.1 Hz, CH), 153.2 (C), 165.9 (C). IR (ATR): 690, 740, 780, 1260, 1280, 1480, 1590, 3040 cm^{-1}. HRMS (EI) *m/z* (M^+) Calcd for $C_{12}H_8F_3NO$: 239.0558; Found: 239.0561.

Butyl 6-phenoxypyridine-3-carboxylate (4ea)

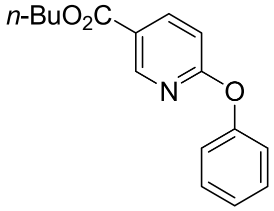

Silica gel column chromatography (hexane/benzene = 16/1) gave 237 mg the product (0.87 mmol, 87%) as a pale yellow oil. ^{1}H NMR (400 MHz, $CDCl_3$): δ 0.97 (3H, t, J = 7.3 Hz), 1.41-1.51 (2H, m), 1.70-1.77 (2H, m), 4.32 (2H, t, J = 6.3 Hz), 6.94 (1H, d, J = 8.3 Hz), 7.15-7.17 (2H, m), 7.24-7.28 (1H, m), 7.42-7.46 (2H, m), 8.28 (1H, dd, J = 2.4, 8.8 Hz), 8.84 (1H, d, J = 2.4 Hz). ^{13}C NMR (100 MHz, $CDCl_3$): δ 13.6 (CH_3), 19.1 (CH_2), 30.6 (CH_2), 64.9 (CH_2), 110.7 (CH), 121.5 (CH + C), 125.4 (CH), 129.8 (CH), 140.6 (CH), 150.4 (CH), 153.4 (C), 165.2 (C), 166.5 (C). IR (ATR): 690, 750, 1250, 1470, 1590, 1720, 2960 cm^{-1}. HRMS (EI) *m/z* (M^+) Calcd for $C_{16}H_{17}NO_3$: 271.1208. Found: 271.1199.

3-Methyl-2-phenoxypyridine (4fa)

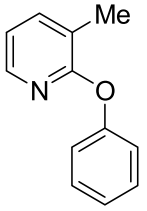

Silica gel column chromatography (hexane/EtOAc = 16/1) gave 167 mg of the product (0.90

mmol, 90%) as a pale yellow oil. ^{1}H NMR (400 MHz, $CDCl_3$): δ 2.35 (3H, s), 6.91 (1H, dd, *J* = 4.9, 7.3 Hz), 7.11 (2H, dd, *J* = 1.0, 8.8 Hz), 7.16-7.19 (1H, m), 7.37-7.41 (2H, m), 7.53 (1H, dd, *J* = 1.0, 7.3 Hz), 8.00 (1H, d, *J* = 4.9 Hz). ^{13}C NMR (100 MHz, $CDCl_3$): δ 15.7 (CH_3), 118.5 (CH), 120.9 (CH), 121.8 (C), 124.1 (CH), 129.3 (CH), 139.6 (CH), 144.6 (CH), 154.4 (C), 161.7 (C). IR (ATR): 690, 730, 750, 1210, 1580, 3060 cm^{-1}. HRMS (EI) *m/z* (M^+) Calcd for $C_{12}H_{11}NO$: 185.0841; Found: 185.0842.

3-Methoxy-2-phenoxypyridine (4ga)

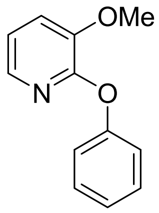

Silica gel column chromatography (hexane/EtOAc = 5/1) gave 162 mg of the product (0.81 mmol, 81%) as pale yellow solids of mp 105-107 °C. ^{1}H NMR (400 MHz, $CDCl_3$): δ 3.95 (3H, s), 6.98 (1H, dd, *J* = 4.9, 8.3 Hz), 7.14-7.23 (4H, m), 7.37-7.41 (2H, m), 7.73 (1H, dd, *J* = 1.5, 4.9 Hz). ^{13}C NMR (100 MHz, $CDCl_3$): δ 55.6 (CH_3), 118.9 (CH), 121.0 (CH), 124.3 (CH), 129.3 (CH), 137.5 (CH), 144.7 (C), 153.4 (C), 154.1 (C). IR (ATR): 690, 790, 1010, 1210, 1480, 1580, 3010 cm^{-1}. HRMS (EI) *m/z* (M^+) Calcd for $C_{12}H_{11}NO_2$: 201.0790; Found: 201.0790.

3-Fluoro-2-phenoxypyridine (4ha)

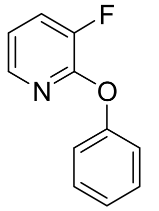

Silica gel column chromatography (hexane/EtOAc = 16/1) gave 171 mg of the product (0.90 mmol, 90%) as a yellow oil. ^{1}H NMR (400 MHz, $CDCl_3$): δ 6.98-7.02 (1H, m), 7.18 (2H, d, *J* = 7.8 Hz), 7.23 (1H, t, *J* = 7.8 Hz), 7.42 (2H, t, *J* = 7.8 Hz), 7.45-7.50 (1H, m), 7.93 (1H, dd, *J* = 1.5, 4.9 Hz). ^{13}C NMR (100 MHz, $CDCl_3$): δ 119.1 (d, *J* = 1.7 Hz, CH), 121.1 (CH), 124.5 (d, *J* = 15.7 Hz, CH), 125.0 (CH), 129.6 (CH), 141.9 (d, *J* = 5.8 Hz, CH), 148.0 (d, *J* = 259.9 Hz, C), 152.4 (d, *J* = 10.8 Hz, C), 153.5 (C). IR (ATR): 690, 790, 1110, 1200, 1590, 3070 cm^{-1}. HRMS (EI) *m/z* Calcd for $C_{11}H_8FNO$ (M^+): 189.0590. Found: 189.0588.

4-Chloro-2-phenoxypyridine (4ia)

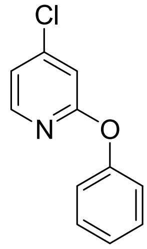

Silica gel column chromatography (hexane/EtOAc = 20/1) gave 168 mg of the product (0.82 mmol, 82%) as a colorless oil. ^{1}H NMR (500 MHz, $CDCl_3$): δ 6.92 (1H, d, *J* = 1.5 Hz), 7.00 (1H, dd, *J* = 1.7, 5.4 Hz), 7.13 (2H, d, *J* = 7.6 Hz), 7.23 (1H, t, *J* = 7.6 Hz), 7.42 (2H, t, *J* = 7.6 Hz), 8.09 (1H, d, *J* = 5.4 Hz). ^{13}C NMR (125 MHz, $CDCl_3$): δ 111.4 (CH), 118.9 (CH), 121.2 (CH), 125.0 (CH), 129.6 (CH), 146.1 (C), 148.2 (CH), 153.4 (C), 164.4 (C). IR (ATR): 690, 760, 1090, 1220, 1490 cm^{-1}. HRMS (EI) *m/z* Calcd for $C_{11}H_8{}^{35}ClNO$: 205.0294. Found: 205.0294.

2-Methoxy-6-phenoxypyridine[17] (4la)

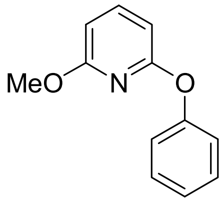

This reaction was conducted on 0.25 mmol scale. Silica gel column chromatography (hexane/ benzene = 10/1) gave 35 mg the product (0.17 mmol, 70%) as a yellow oil. ^{1}H NMR (500 MHz, $CDCl_3$): δ 3.81 (3H, s), 6.30 (1H, d, *J* = 7.8 Hz), 6.44 (1H, d, *J* = 7.8 Hz), 7.15 (2H, d, *J* = 8.1 Hz), 7.17-7.20 (1H, m), 7.38 (2H, t, *J* = 8.1 Hz), 7.53 (1H, t, *J* = 7.8 Hz). ^{13}C NMR (100 MHz, $CDCl_3$): δ 53.4 (CH_3), 101.4 (CH), 104.2 (CH), 121.1 (CH), 124.5 (CH), 129.6 (CH), 141.4 (CH), 154.4 (C), 162.5 (C), 163.5 (C). IR (ATR): 690, 790, 1040, 1200, 1230, 1570, 3060 cm^{-1}. EIMS *m/z*: 201 (M^+).

4-Phenoxypyridine (4ka)

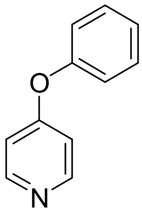

Silica gel column chromatography (hexane/EtOAc = 2/1) gave 142 mg of the product (0.83 mmol, 83%) as a colorless oil. ^{1}H NMR (400 MHz, $CDCl_3$): δ 6.83-6.85 (2H, m), 7.10-7.12 (2H, m),

7.24-7.28 (1H, m), 7.44 (2H, t, J = 7.8 Hz), 8.46-8.48 (2H, m). ^{13}C NMR (100 MHz, $CDCl_3$): δ 112.1 (CH), 120.9 (CH), 125.5 (CH), 130.2 (CH), 151.5 (CH), 154.0 (C), 164.8 (C). IR (ATR): 690, 800, 1210, 1260, 1480, 1570, 3030 cm^{-1}. HRMS (EI) *m/z* (M^+) Calcd for $C_{11}H_9NO$: 171.0684; Found: 171.0687.

2-(4-*tert*-Butylphenoxy)pyridine[19] (4ah)

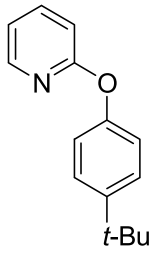

Silica gel column chromatography (hexane/EtOAc = 16/1) gave 216 mg of the product (0.95 mmol, 95%) as white solids of mp 110-112 °C. ^{1}H NMR (400 MHz, $CDCl_3$): δ 1.33 (9H, s), 6.89 (1H, d, J = 8.3 Hz), 6.96-7.00 (1H, m), 7.05-7.09 (2H, m), 7.39-7.43 (2H, m), 7.65-7.69 (1H, m), 8.21 (1H, dd, J = 2.0, 4.9 Hz). ^{13}C NMR (100 MHz, $CDCl_3$): δ 31.2 (CH_3), 34.1 (C), 111.2 (CH), 118.1 (CH), 120.4 (CH), 126.4 (CH), 139.2 (CH), 147.1 (C), 147.6 (CH), 151.6 (C), 163.8 (C). IR (ATR): 770, 840, 1210, 1590, 3060 cm^{-1}. EIMS *m/z*: 227 (M^+).

2-[4-(Trifluoromethyl)phenoxy]pyridine[19] (4ai)

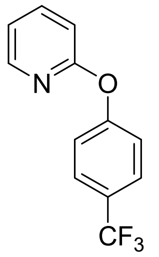

Silica gel column chromatography (hexane/EtOAc = 16/1) gave 239 mg of the product (0.99 mmol, 99%) as colorless oil. ^{1}H NMR (400 MHz, $CDCl_3$): δ 7.00 (1H, d, J = 8.3 Hz), 7.05-7.08 (1H, m), 7.24-7.26 (2H, m), 7.66 (2H, d, J = 8.3 Hz), 7.73-7.77 (1H, m), 8.22 (1H, d, J = 3.4 Hz). ^{13}C NMR (100 MHz, $CDCl_3$): δ 112.2 (CH), 119.3 (CH), 121.1 (CH), 124.1 (q, J = 272.3 Hz, C), 126.4 (q, J = 32.3 Hz, C), 126.9 (q, J = 3.3 Hz, CH), 139.8 (CH), 147.7 (CH), 157.0 (C), 162.8 (C). IR (ATR): 780, 850, 1120, 1240, 1470, 1570, 3070 cm^{-1}. EIMS *m/z*: 239 (M^+).

2-(4-Fluorophenoxy)pyridine[17] (4ad)

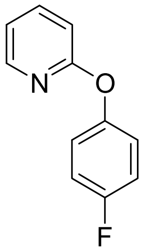

Silica gel column chromatography (hexane/EtOAc = 10/1) gave 172 mg of the product (0.91 mmol, 91%) as a yellow oil. ^{1}H NMR (400 MHz, $CDCl_3$): δ 6.91 (1H, d, *J* = 8.3 Hz), 7.00 (1H, dd, *J* = 4.9, 6.8 Hz), 7.06-7.13 (4H, m), 7.67-7.71 (1H, m), 8.18-8.19 (1H, m). ^{13}C NMR (100 MHz, $CDCl_3$): δ 111.4 (CH), 116.3 (d, *J* = 23.2 Hz, CH), 118.5 (CH), 122.8 (d, *J* = 8.3 Hz, CH), 139.5 (CH), 147.7 (CH), 149.9 (C), 159.7 (d, *J* = 242.5 Hz, C), 163.8 (C). IR (ATR): 780, 880, 1190, 1220, 1270, 1590, 3060 cm^{-1}. EIMS *m/z*: 189 (M^+).

2-(4-Chlorophenoxy)pyridine[19] (4ae)

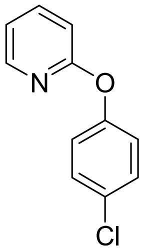

Silica gel column chromatography (hexane/EtOAc = 16/1) gave 197 mg of the product (0.96 mmol, 96%) as colorless oil. ^{1}H NMR (400 MHz, $CDCl_3$): δ 6.94 (1H, d, *J* = 8.3 Hz), 7.00-7.03 (1H, m), 7.09 (2H, d, *J* = 8.8 Hz), 7.36 (2H, d, *J* = 8.8 Hz), 7.69-7.73 (1H, m), 8.19 (1H, d, *J* = 3.4 Hz). ^{13}C NMR (100 MHz, $CDCl_3$): δ 111.6 (CH), 118.7 (CH), 122.6 (CH), 129.6 (CH), 129.8 (C), 139.6 (CH), 147.6 (CH), 152.6 (C), 163.3 (C). IR (ATR): 770, 840, 1080, 1240, 1580, 3060 cm^{-1}. EIMS *m/z*: 205 (M^+).

2-[4-(Methoxycarbonyl)phenoxy]pyridine[17] (4aj)

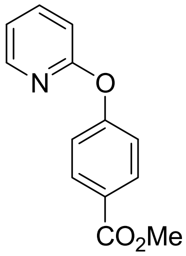

This reaction was conducted on 0.25 mmol scale. Silica gel column chromatography (hexane/

benezene = 1/4) gave 55 mg of the product (0.24 mmol, 96%) as a yellow oil. ^{1}H NMR (400 MHz, $CDCl_3$): δ 3.92 (3H, s), 6.98 (1H, d, *J* = 8.3 Hz), 7.07 (1H, dd, *J* = 5.4, 6.8 Hz), 7.18 (2H, d, *J* = 8.8 Hz), 7.72-7.76 (1H, m), 8.09 (2H, d, *J* = 8.8 Hz), 8.22-8.24 (1H, m). ^{13}C NMR (100 MHz, $CDCl_3$): δ 52.0 (CH_3), 112.4 (CH), 119.3 (CH), 120.4 (CH), 126.1 (C), 131.5 (CH), 139.8 (CH), 147.9 (CH), 158.4 (C), 162.9 (C), 166.6 (C). IR (ATR): 770, 850, 1190, 1220, 1260, 1590, 3070 cm^{-1}. EIMS *m/z*: 229 (M^+).

2-(2-Methylphenoxy)pyridine[19] (4ak)

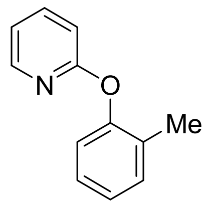

Silica gel column chromatography (hexane/EtOAc = 16/1) gave 184 mg of the product (0.99 mmol, 99%) as yellow oil. ^{1}H NMR (400 MHz, $CDCl_3$): δ 2.18 (3H, s), 6.85 (1H, d, *J* = 8.3 Hz), 6.94-6.97 (1H, m), 7.06 (1H, d, *J* = 8.3 Hz), 7.14 (1H, t, *J* = 7.3 Hz), 7.22-7.29 (2H, m), 7.64-7.69 (1H, m), 8.17-8.19 (1H, m). ^{13}C NMR (100 MHz, $CDCl_3$): δ 16.2 (CH_3), 110.6 (CH), 117.9 (CH), 121.7 (CH), 125.1 (CH), 127.1 (CH), 130.7 (C), 131.3 (CH), 139.3 (CH), 147.8 (CH), 152.2 (C), 163.7 (C). IR (ATR): 710, 770, 1240, 1470, 1570, 3060 cm^{-1}. EIMS *m/z*: 185 (M^+).

2-(4-Methoxyphenoxy)pyridine[17] (4ac)

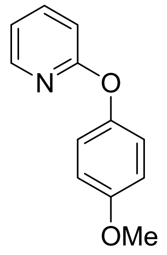

This reaction was conducted on 0.5 mmol scale. Silica gel column chromatography (hexane/ EtOAc = 5/1) gave 3 mg of the product (0.015 mmol, 3%) as a yellow oil. ^{1}H NMR (500 MHz, $CDCl_3$): δ 3.82 (3H, s), 6.87 (1H, d, *J* = 8.3 Hz), 6.92-6.97 (3H, m), 7.06-7.08 (2H, m), 7.65-7.67 (1H, m), 8.18 (1H, dd, *J* = 1.7, 4.9 Hz). ^{13}C NMR (125 MHz, $CDCl_3$): δ 55.5 (CH_3), 111.0 (CH), 114.7 (CH), 118.0 (CH), 122.3 (CH), 139.2 (CH), 147.3 (C), 147.6 (CH), 156.5 (C), 164.2 (C). IR (ATR): 780, 880, 1030, 1200, 1230, 1590, 3060 cm^{-1}. EIMS *m/z*: 201 (M^+).

Diphenyliodonium 2-oxo-5-(trifluoromethyl)-*2H*-pyridin-1-ide (5)

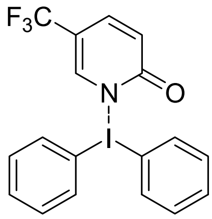

Na (690 mg, 30 mmol) was added to MeOH (40 mL) at 0 °C and dissolved. To the solution was added 5-(trifluoromethyl)-2-pyridone (4.89 g, 30 mmol), and the reaction mixture was stirred at room temperature for 12 h. MeOH was removed to give 5.52 g of sodium 2-oxo-5-(trifluoromethyl)-*2H*-pyridin-1-ide (29.8 mmol, 99%) as white solids of mp 114-115 °C. ^{1}H NMR (500 MHz, DMSO-d_6): δ 5.87 (1H, d, J = 8.8 Hz), 7.18 (1H, dd, J = 2.2, 8.8 Hz), 7.91 (1H, d, J = 2.2 Hz). ^{13}C NMR (125 MHz, DMSO-d_6): δ 106.0 (q, J = 31.5 Hz, C), 113.6 (CH), 126.5 (q, J = 268.0 Hz, C), 133.2 (q, J = 2.9 Hz, CH), 145.7 (q, J = 4.8 Hz, CH), 173.8 (C). ^{19}F NMR (470 MHz, DMSO-d_6): δ −57.5. IR (ATR): 840, 1110, 1480, 3080 cm^{-1}. HRMS (FAB) *m/z* Calcd for $C_6H_4F_3NONa$ $[M+H]^+$: 186.0143. Found: 186.0146. Anal. Calcd for $C_6H_3F_3NONa\cdot 1/2H_2O$: C, 37.13; H, 2.08; N, 7.22. Found: C, 37.37; H, 2.05; N, 7.18.

A reaction flask was charged with diphenyliodonium triflate (860 mg, 2.0 mmol), and CH_2Cl_2 (135 mL) was added. To the solution was added 1.1 M aqueous solution of sodium 2-oxo-5-(trifluoromethyl)-*2H*-pyridin-1-ide (3.70 g, 20 mmol), and the reaction mixture was stirred at 0 °C for 12 h. The resulting mixture was extracted with CH_2Cl_2. The organic layers were washed with H_2O and concentrated. Et_2O (50 mL) was added and the mixture was stirred at room temperature for 30 min to precipitate solids. The solids were filtrated, washed with Et_2O, and dried in vacuo to give 755 mg of the product (1.70 mmol, 85% yield) as white solids of mp 143-144 °C. ^{1}H NMR (500 MHz, DMSO-d_6): δ 5.85 (1H, d, J = 9.1 Hz), 7.17 (1H, dd, J = 2.9, 9.1 Hz), 7.46 (4H, t, J = 7.6 Hz), 7.58 (2H, t, J = 7.6 Hz), 7.79 (1H, d, J = 2.0 Hz), 8.18 (4H, d, J = 7.6 Hz). ^{13}C NMR (125 MHz, DMSO-d_6): δ 106.2 (q, J = 32.4 Hz, C), 113.4 (CH), 119.9 (C), 126.3 (q, J = 268.9 Hz, C), 131.0 (CH), 131.1 (CH), 133.0 (q, J = 1.9 Hz, CH), 134.8 (CH), 145.3 (q, J = 3.8 Hz, CH), 172.7 (C). ^{19}F NMR (470 MHz, DMSO-d_6): δ −57.7. IR (ATR): 840, 1100, 1320, 1500, 1600 cm^{-1}. HRMS (FAB$^+$) *m/z* Calcd for $C_{12}H_{10}I$ $[M-(5\text{-}CF_3\text{-}C_5H_3NO)]^+$: 280.9827. Found: 280.9826. HRMS (FAB$^-$) *m/z* Calcd for $C_6H_3F_3NO$ $[M-(Ph_2I)]^-$: 162.0167. Found: 162.0171. Anal. Calcd for $C_{18}H_{13}F_3INO$: C, 48.78; H, 2.96; N, 3.16. Found: C, 48.53; H, 2.98; N, 3.17.

Compound 3ga (CCDC 1999363)

Bond precision:	C-C = 0.0031 A		Wavelength=0.71075
Cell:	a=6.571(3)	b=12.434(5)	c=12.168(6)
	alpha=90	beta=93.376(7)	gamma=90
Temperature: 93 K			

	Calculated	Reported
Volume	992.5(8)	992.4(8)
Space group	P 21/c	P 1 21/c 1
Hall group	-P 2ybc	-P 2ybc
Moiety formula	C12 H11 N O2	C12 H11 N O2
Sum formula	C12 H11 N O2	C12 H11 N O2
Mr	201.22	201.22
Dx,g cm-3	1.347	1.347
Z	4	4
Mu (mm-1)	0.092	0.092
F000	424.0	424.0
F000'	424.20	
h,k,lmax	8,16,15	8,15,15
Nref	2284	2275
Tmin,Tmax	0.991,0.998	0.886,0.998
Tmin'	0.980	

Correction method= # Reported T Limits: Tmin=0.886 Tmax=0.998 AbsCorr = MULTI-SCAN

Data completeness= 0.996 Theta(max)= 27.492

R(reflections)= 0.0693(1776) wR2(reflections)= 0.1506(2275)

S = 1.189 Npar= 136

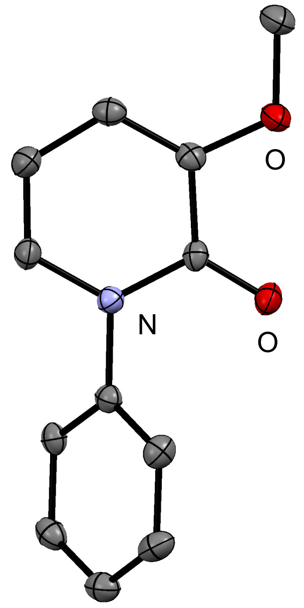

Compound 4ga (CCDC 1999364)

Bond precision:	C-C = 0.0032 A		Wavelength=0.71075
Cell:	a=9.935(5)	b=7.985(4)	c=13.410(7)
	alpha=90	beta=110.758(9)	gamma=90
Temperature:	93 K		

	Calculated	Reported
Volume	994.8(9)	994.8(9)
Space group	P 21/n	P 1 21/n 1
Hall group	-P 2yn	-P 2yn
Moiety formula	C12 H11 N O2	C12 H11 N O2
Sum formula	C12 H11 N O2	C12 H11 N O2
Mr	201.22	201.22
Dx,g cm-3	1.344	1.343
Z	4	4
Mu (mm-1)	0.092	0.092
F000	424.0	424.0
F000'	424.20	
h,k,lmax	12,10,17	12,10,17
Nref	2278	2276
Tmin,Tmax	0.989,0.998	0.854,0.998
Tmin'	0.986	

Correction method= # Reported T Limits: Tmin=0.854 Tmax=0.998 AbsCorr = MULTI-SCAN

Data completeness= 0.999 Theta(max)= 27.446

R(reflections)= 0.0617(1494) wR2(reflections)= 0.1373(2276)

S = 1.090 Npar= 136

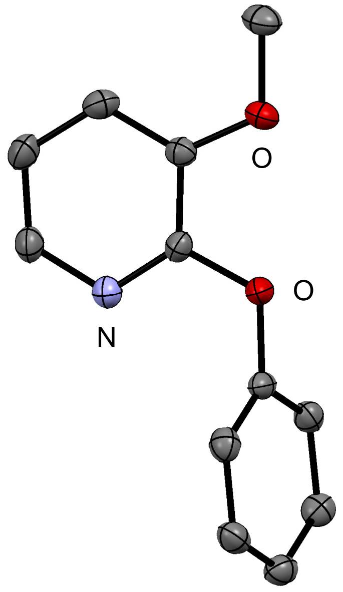

Compound 5 (CCDC 1999366)

Bond precision:	C-C = 0.0071 A		Wavelength=0.71075
Cell:	a=6.0218(15)	b=15.569(4)	c=17.415(4)
	alpha=90	beta=91.278(5)	gamma=90
Temperature:	93 K		

	Calculated	Reported
Volume	1632.3(7)	1632.3(7)
Space group	P 21/n	P 1 21/n 1
Hall group	-P 2yn	-P 2yn
Moiety formula	C12 H10 I, C6 H3 F3 N O	C18 H13 F3 I N O
Sum formula	C18 H13 F3 I N O	C18 H13 F3 I N O
Mr	443.19	443.21
Dx,g cm-3	1.804	1.803
Z	4	4
Mu (mm-1)	1.998	1.998
F000	864.0	864.0
F000'	862.56	
h,k,lmax	7,20,22	7,20,22
Nref	3733	3666
Tmin,Tmax	0.887,0.942	0.833,0.942
Tmin'	0.819	

Correction method= # Reported T Limits: Tmin=0.833 Tmax=0.942 AbsCorr = MULTI-SCAN

Data completeness= 0.982 Theta(max)= 27.475

R(reflections)= 0.0417(3118) wR2(reflections)= 0.1059(3666)

S = 1.274 Npar= 217

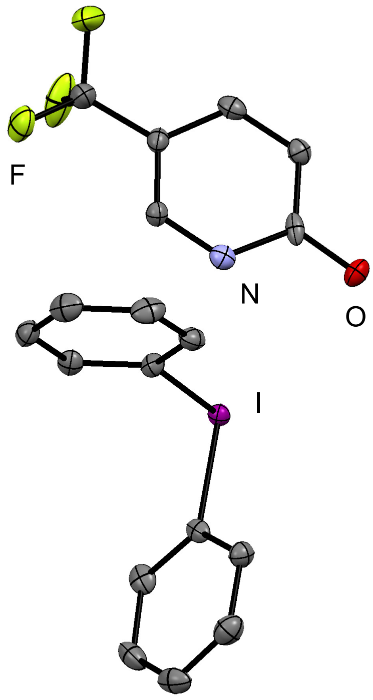

Comparison between the effects of FPh and ClPh

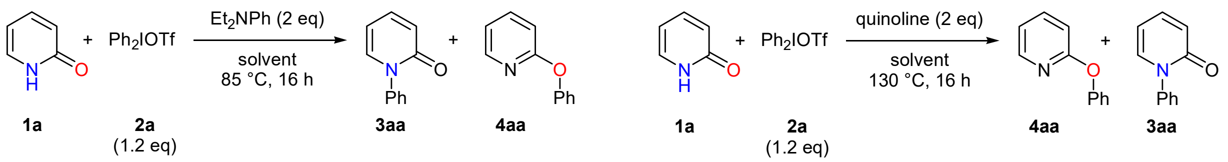

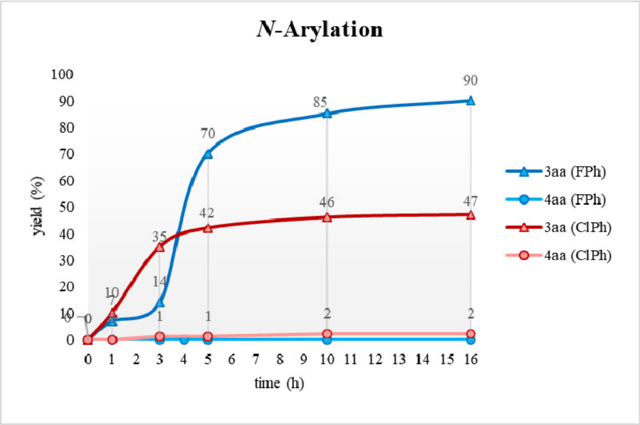

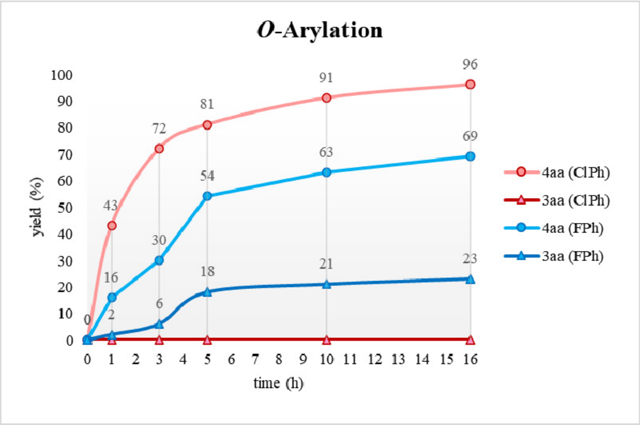

References

(1) S. Hanessian, O. M. Saavedra, V. Mascitti, W. Marterer, R. Oehrlein, C.-P. Mak, *Tetrahedron*, 2001, **57**, 3267-3280.

(2) C. Kaneko, K. Uchiyama, M. Sato, N. Katagiri, *Chem. Pharm. Bull.*, 1986, **34**, 3658-3671.

(3) M. Bielawski, M. Zhu, B. Olofsson, *Adv. Synth. Catal.*, 2007, **349**, 2610-2618.

(4) M. Bielawski, D. Aili, B. Olofsson, *J. Org. Chem.*, 2008, **73**, 4602-4607.

(5) R. Beaud, R. J. Phipps, M. J. Gaunt, *J. Am. Chem. Soc.*, 2016, **138**, 13183-13186.

(6) D. W. Lin, T. Masuda, M. B. Biskup, J. D. Nelson, P. S. Baran, *J. Org. Chem.*, 2011, **76**, 1013-1030.

(7) M. Kuriyama, N. Hamaguchi, O. Onomura, *Chem. Eur. J.*, 2012, **18**, 1591-1594.

(8) Y. Gu, D. Chang, X. Leng, Y. Gu, Q. Shen, *Organometallics*, 2015, **34**, 3065-3071.

(9) R. A. Altman, S. L. Buchwald, *Org. Lett.*, 2007, **9**, 643-646.

(10) X.-H. Li, A.-H. Ye, C. Liang, D.-L. Mo, *Synthesis*, 2018, **50**, 1699-1710.

(11) J. Li, Y. Yang, Z. Wang, B. Feng, J. You, *Org. Lett.*, 2017, **19**, 3083-3086.

(12) C. Crifar, P. Petiot, T. Ahmad, A. Gagnon, *Chem. Eur. J.*, 2014, **20**, 2755-2760.

(13) J. G. Sośnicki, P. Dzitkowski, Ł. Struk, *Eur. J. Org. Chem.*, 2015, **2015**, 5189-5198.

(14) K. A. Kumar, P. Kannabonia, C. K. Jaladanki, P. V. Bharatam, P. Das, *ChemstrySelect*, 2016, **1**, 601-607.

(15) D. C. McAteer, E. Javed, L. Huo, S. Huo, *Org. Lett.*, 2017, **19**, 1606-1609.

(16) K. Ikegai, Y. Nagata, T. Mukaiyama, *Bull. Chem. Soc. Jpn.* 2006, **79**, 761-767.

(17) R. Takise, R. Isshiki, K. Muto, K. Itami, J. Yamaguchi, *J. Am. Chem. Soc.*, 2017, **139**, 3340-3343.

(18) J. Niu, H. Zhou, Z. Li, J. Xu, S. Hu, *J. Org. Chem.*, 2008, **73**, 7814-7817.

(19) H. Kinuta, M. Tobisu, N. Chatani, *J. Am. Chem. Soc.*, 2015, **137**, 1593-1600.

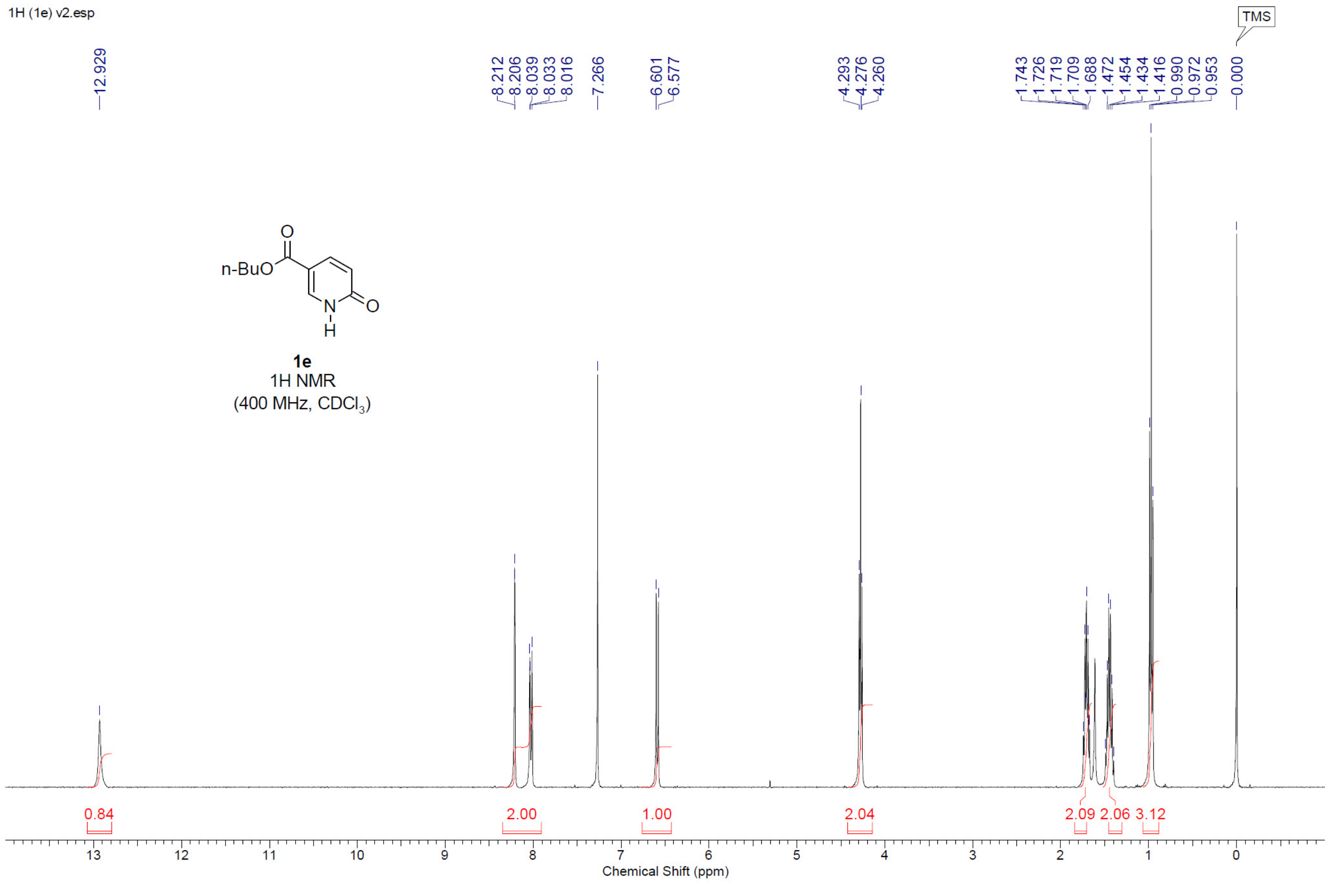
1H (1e) v2.esp
12.929
8.212
8.206
8.039
8.033
8.016
7.266
6.601
6.577
4.293
4.276
4.260
1.743
1.726
1.719
1.709
1.688
1.472
1.454
1.434
1.416
0.990
0.972
0.953
0.000
TMS
n-BuO
O
N
H
O
1e
1H NMR
(400 MHz, CDCl3)
0.84
2.00
1.00
2.04
2.09
2.06
3.12
Chemical Shift (ppm)

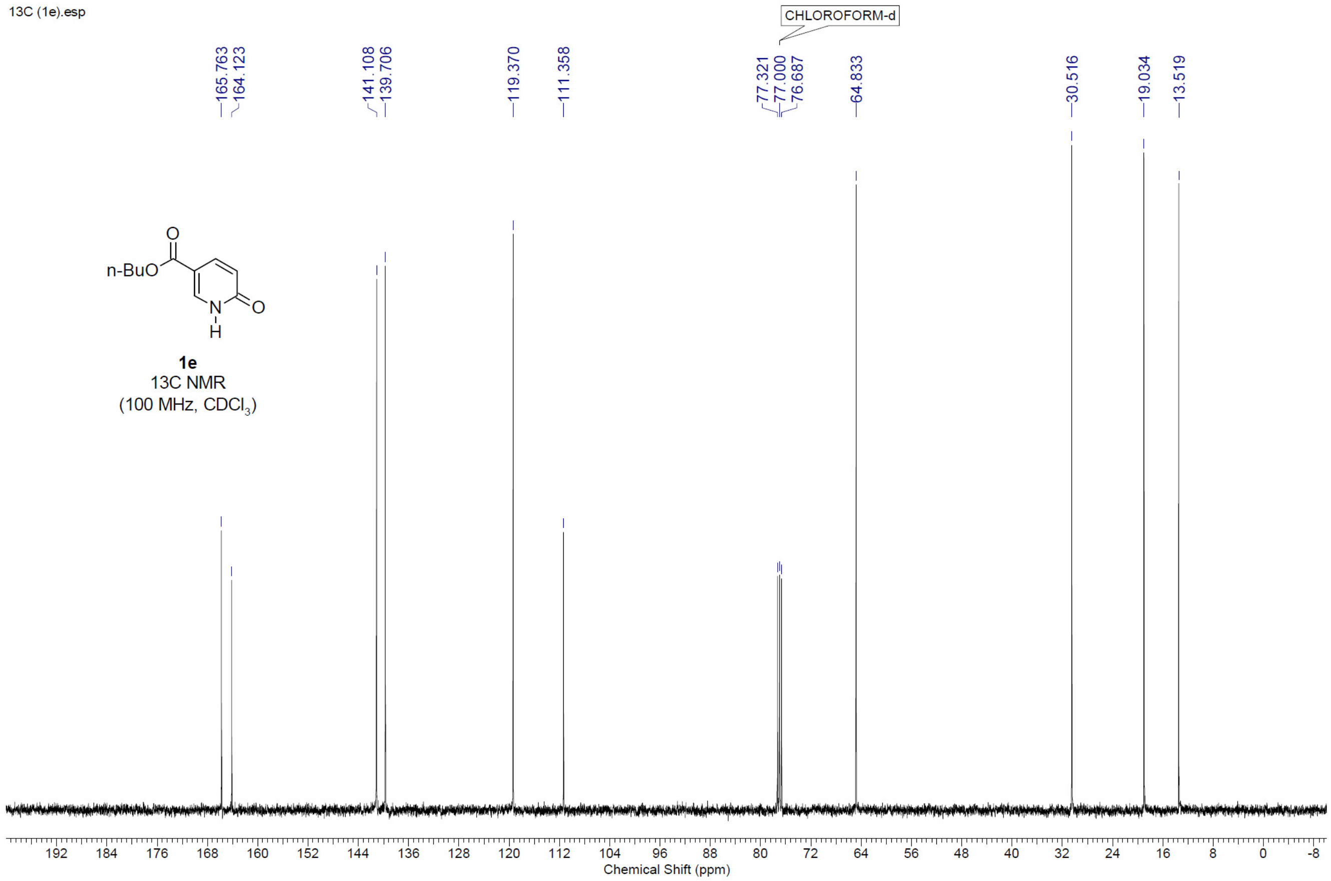
13C (1e).esp
165.763
164.123
141.108
139.706
119.370
111.358
77.321
77.000
76.687
CHLOROFORM-d
64.833
30.516
19.034
13.519
n-BuO
O
N
H
O
1e
13C NMR
(100 MHz, CDCl3)
Chemical Shift (ppm)

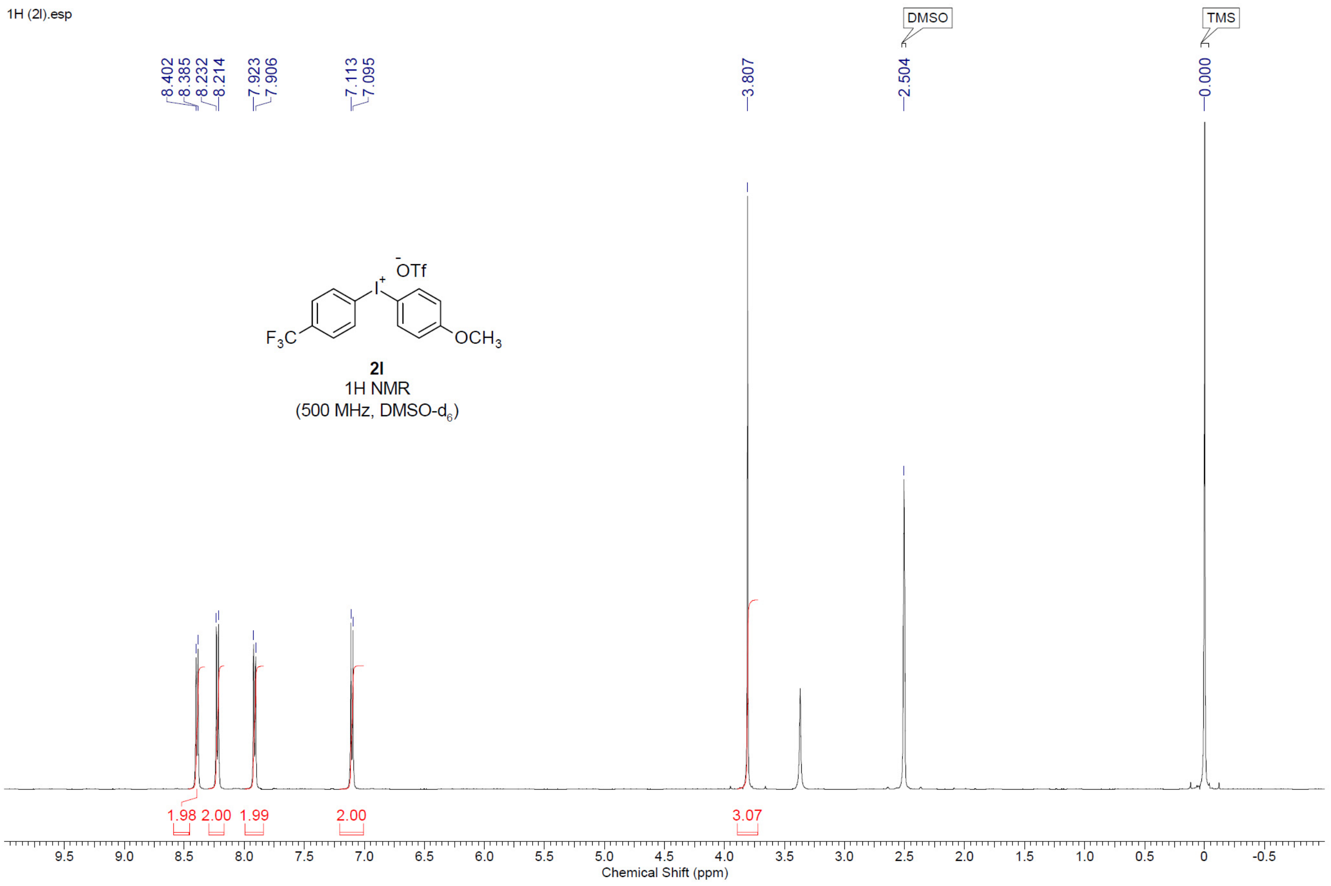
1H (2l).esp
8.402
8.385
8.232
8.214
7.923
7.906
7.113
7.095
3.807
2.504
0.000
DMSO
TMS
OTf
F3C
OCH3
2l
1H NMR
(500 MHz, DMSO-d6)
1.98
2.00
1.99
2.00
3.07
Chemical Shift (ppm)

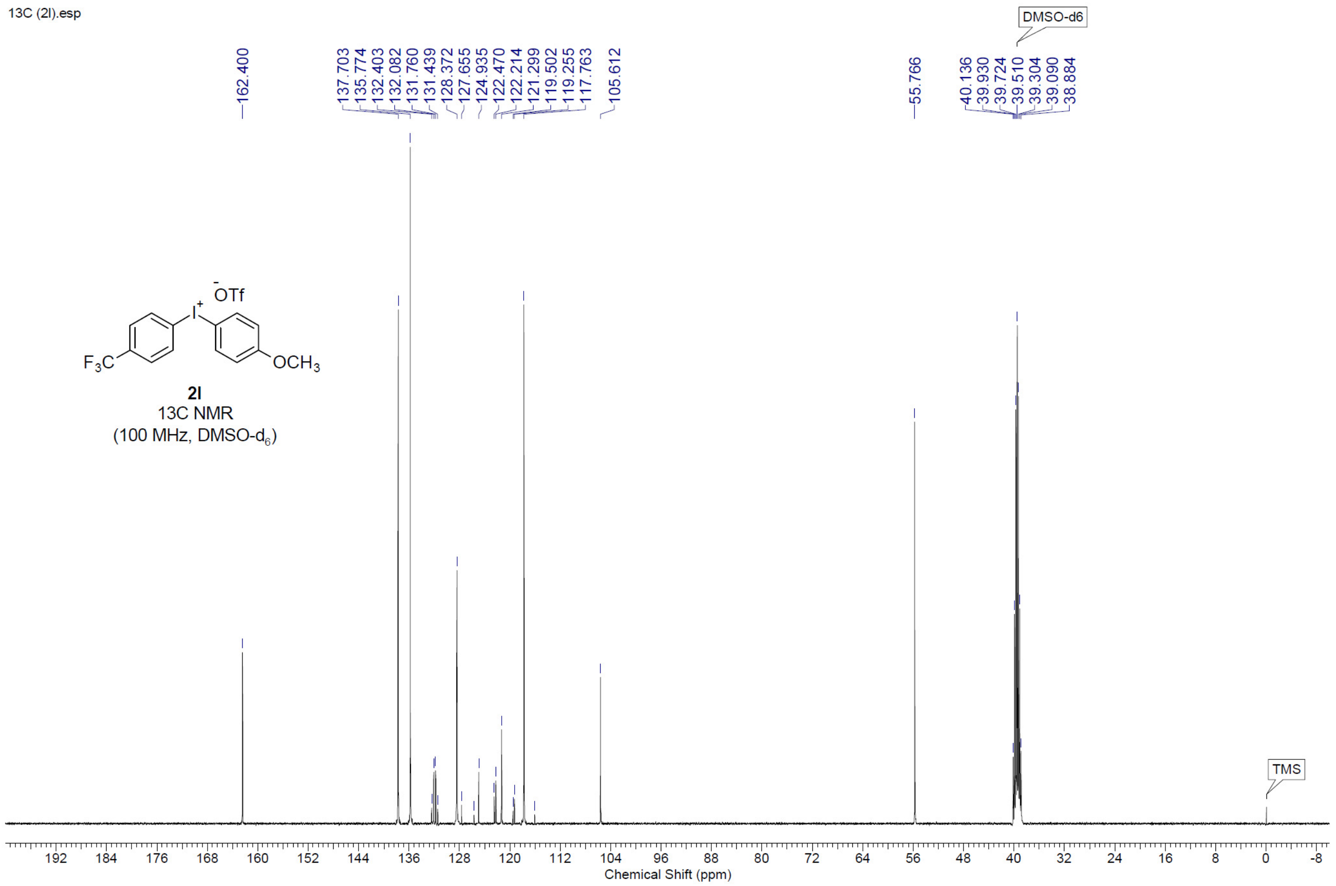
13C (2l).esp
162.400
137.703
135.774
132.403
132.082
131.760
131.439
128.372
127.655
124.935
122.470
122.214
121.299
119.502
119.255
117.763
105.612
55.766
40.136
39.930
39.724
39.510
39.304
39.090
38.884
DMSO-d6
TMS
OTf
F3C
OCH3
2l
13C NMR
(100 MHz, DMSO-d6)
Chemical Shift (ppm)

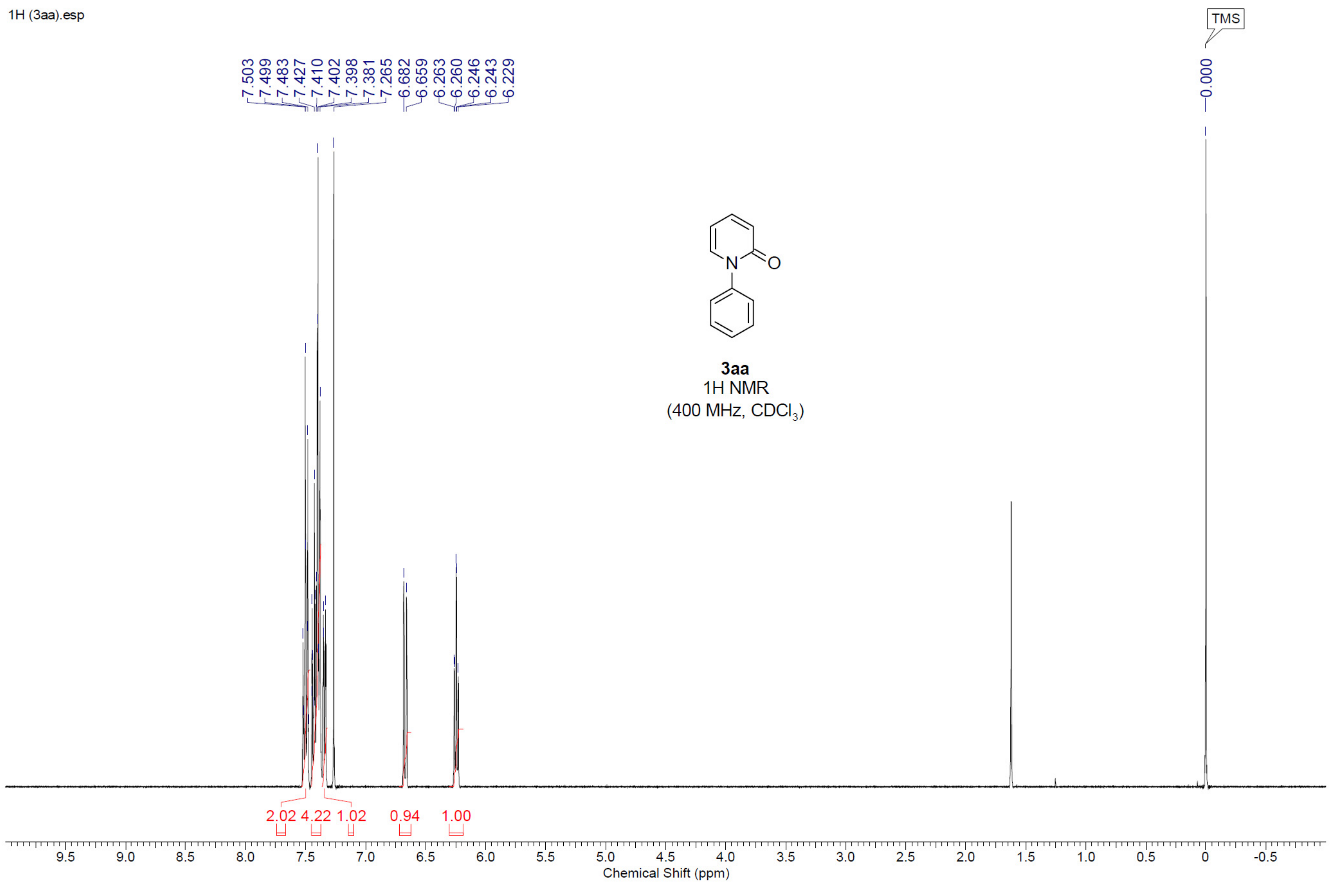
1H (3aa).esp
TMS
7.503
7.499
7.483
7.427
7.410
7.402
7.398
7.381
7.265
6.682
6.659
6.263
6.260
6.246
6.243
6.229
0.000
3aa
1H NMR
(400 MHz, CDCl3)
2.02 4.22 1.02 0.94 1.00
Chemical Shift (ppm)

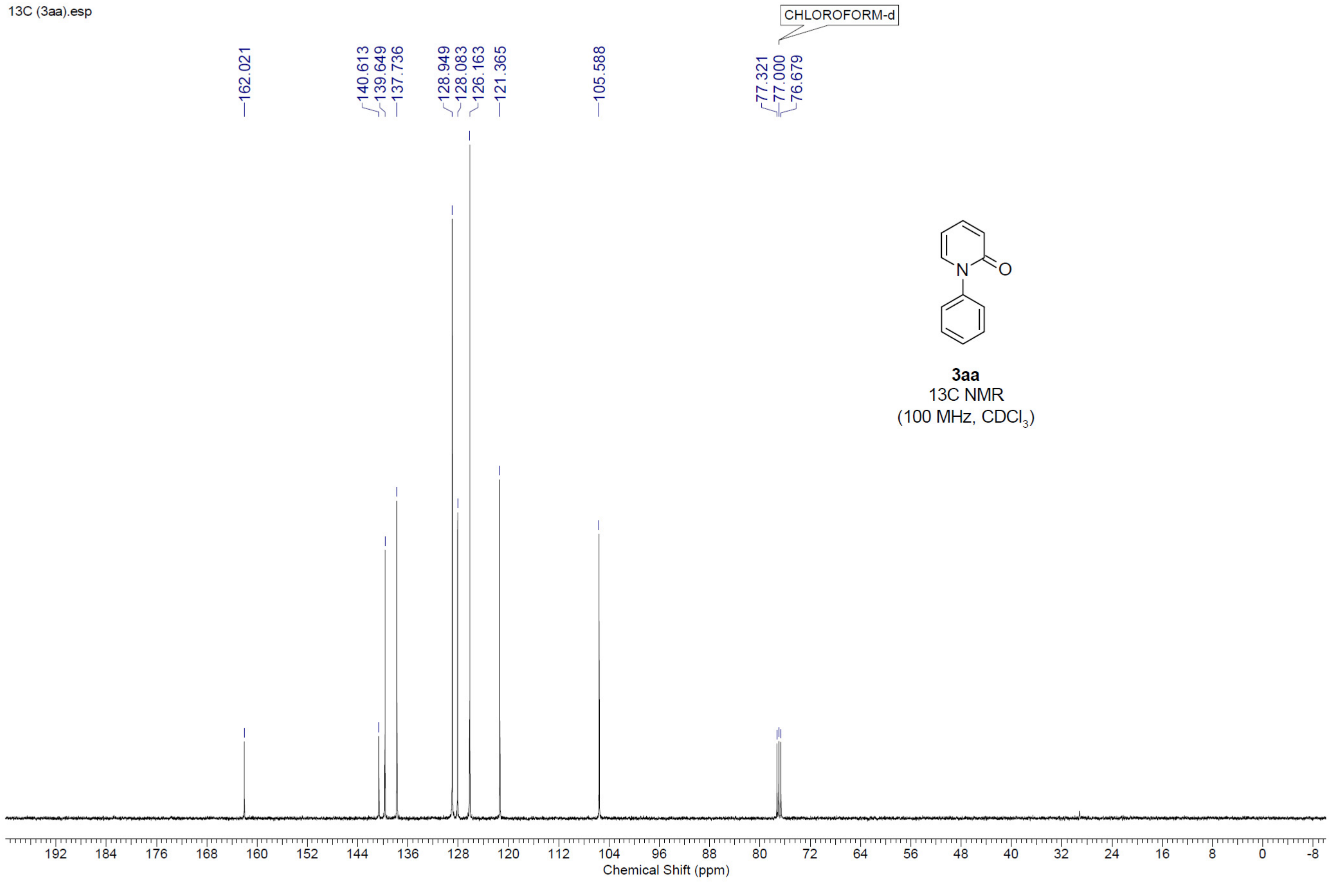
13C (3aa).esp
CHLOROFORM-d
162.021
140.613
139.649
137.736
128.949
128.083
126.163
121.365
105.588
77.321
77.000
76.679
3aa
13C NMR
(100 MHz, CDCl3)
Chemical Shift (ppm)

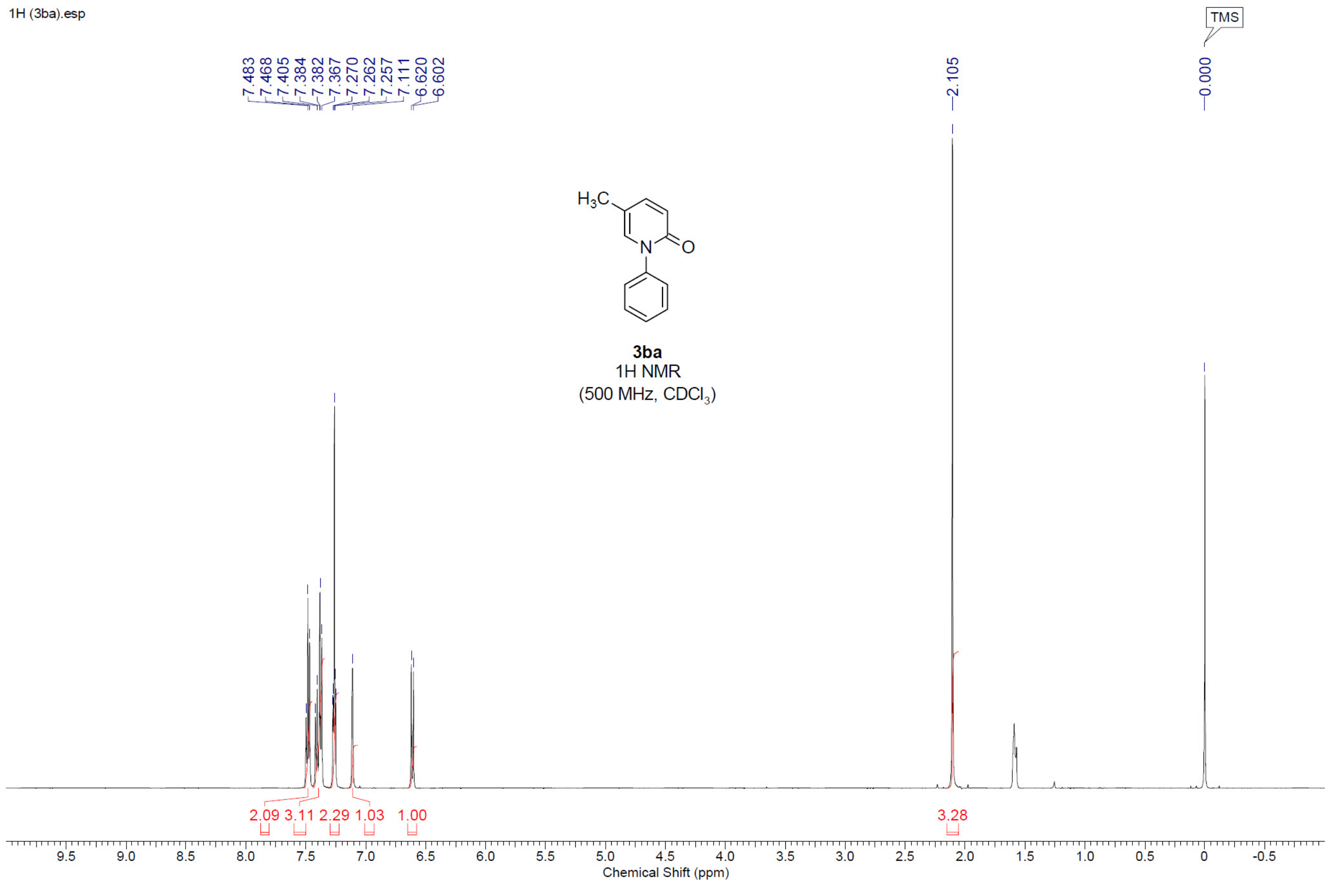
1H (3ba).esp
TMS
7.483
7.468
7.405
7.384
7.382
7.367
7.270
7.262
7.257
7.111
6.620
6.602
2.105
0.000
H3C
N
O
3ba
1H NMR
(500 MHz, CDCl3)
2.09
3.11
2.29
1.03
1.00
3.28
9.5
9.0
8.5
8.0
7.5
7.0
6.5
6.0
5.5
5.0
4.5
4.0
3.5
3.0
2.5
2.0
1.5
1.0
0.5
0
-0.5
Chemical Shift (ppm)

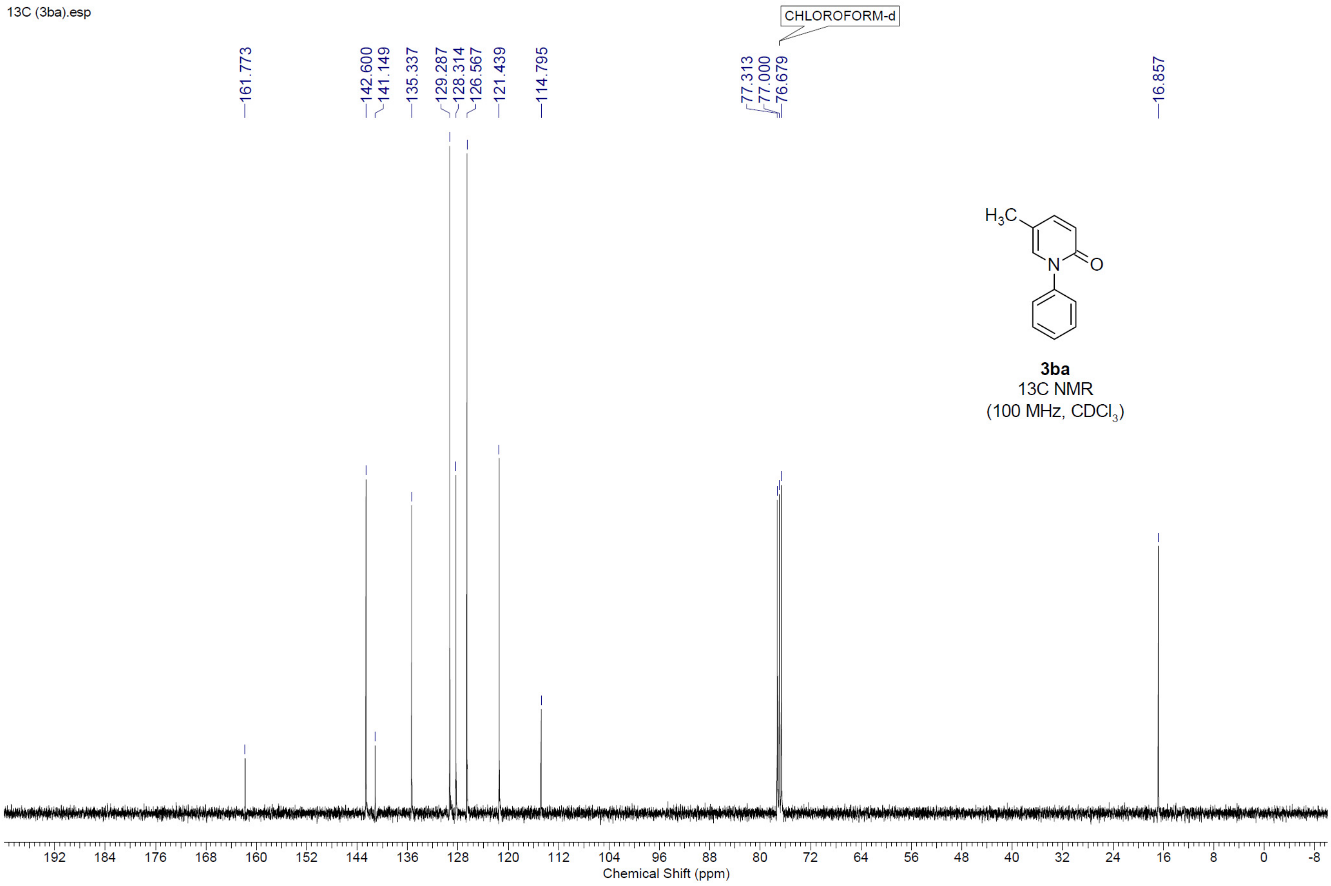
13C (3ba).esp
CHLOROFORM-d
161.773
142.600
141.149
135.337
129.287
128.314
126.567
121.439
114.795
77.313
77.000
76.679
16.857
H3C
N
O
3ba
13C NMR
(100 MHz, CDCl3)
192
184
176
168
160
152
144
136
128
120
112
104
96
88
80
72
64
56
48
40
32
24
16
8
0
-8
Chemical Shift (ppm)

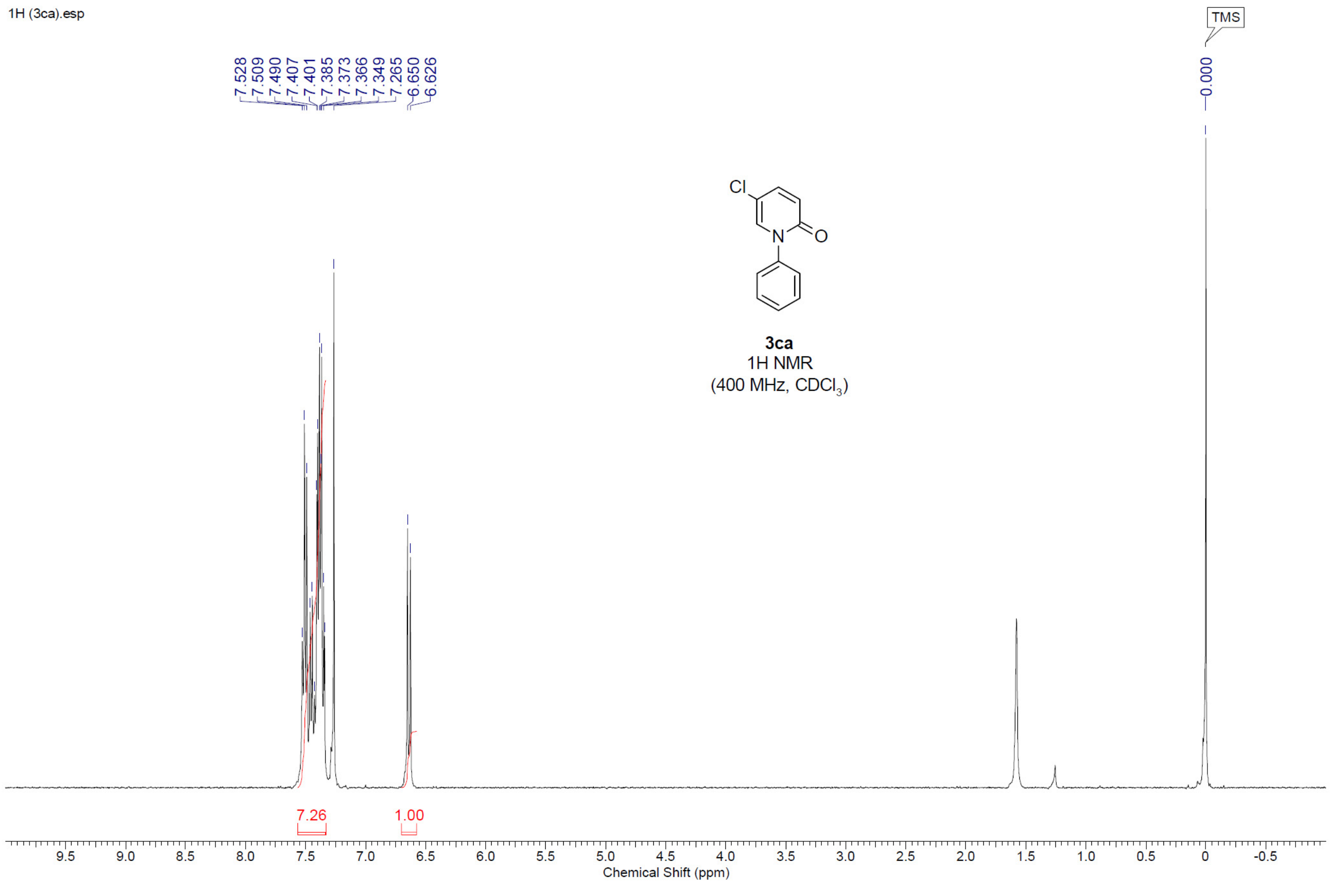
1H (3ca).esp
7.528
7.509
7.490
7.407
7.401
7.385
7.373
7.366
7.349
7.265
6.650
6.626
TMS
0.000
Cl
N
O
3ca
1H NMR
(400 MHz, CDCl3)
7.26
1.00
Chemical Shift (ppm)

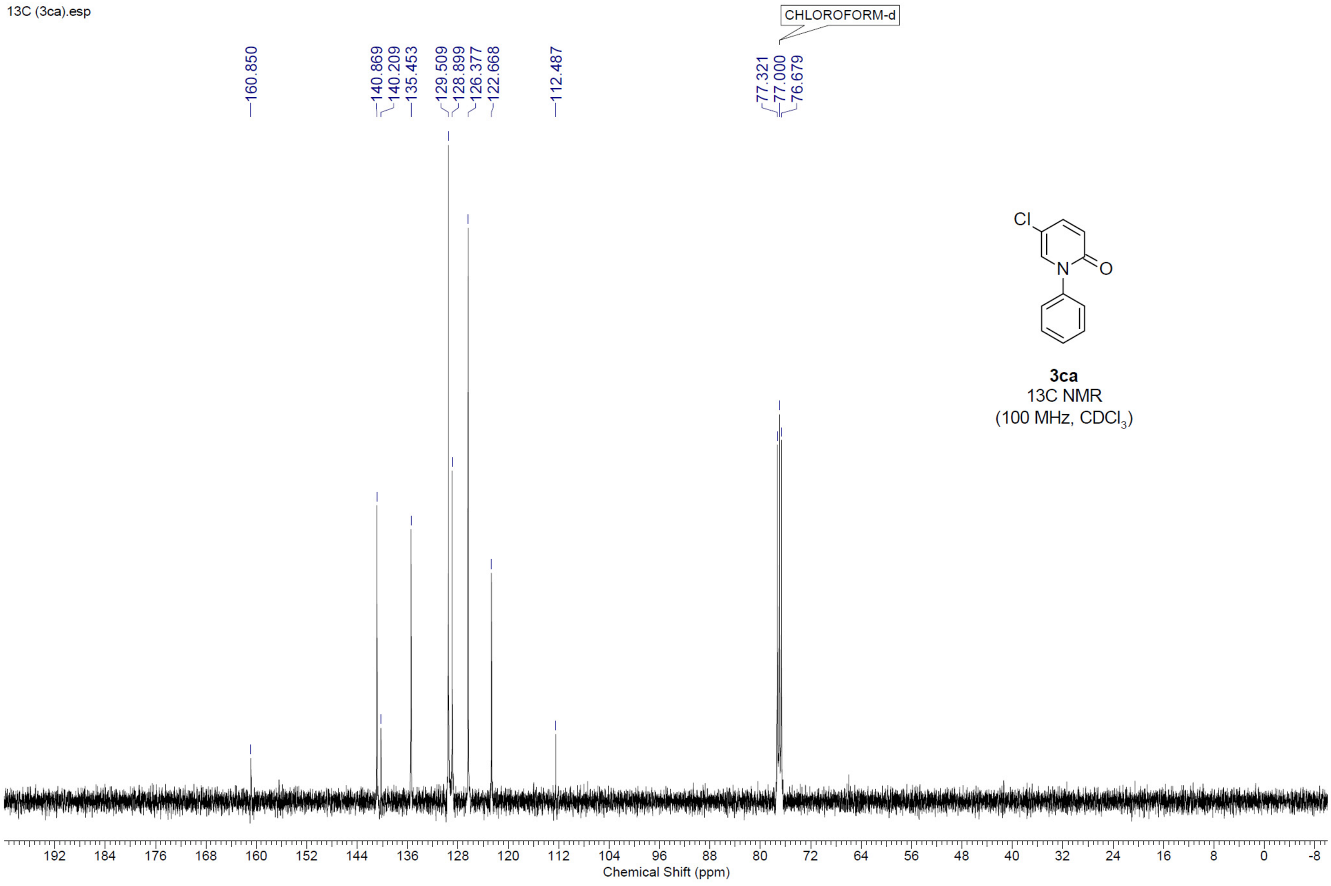
13C (3ca).esp
160.850
140.869
140.209
135.453
129.509
128.899
126.377
122.668
112.487
CHLOROFORM-d
77.321
77.000
76.679
Cl
N
O
3ca
13C NMR
(100 MHz, CDCl3)
Chemical Shift (ppm)

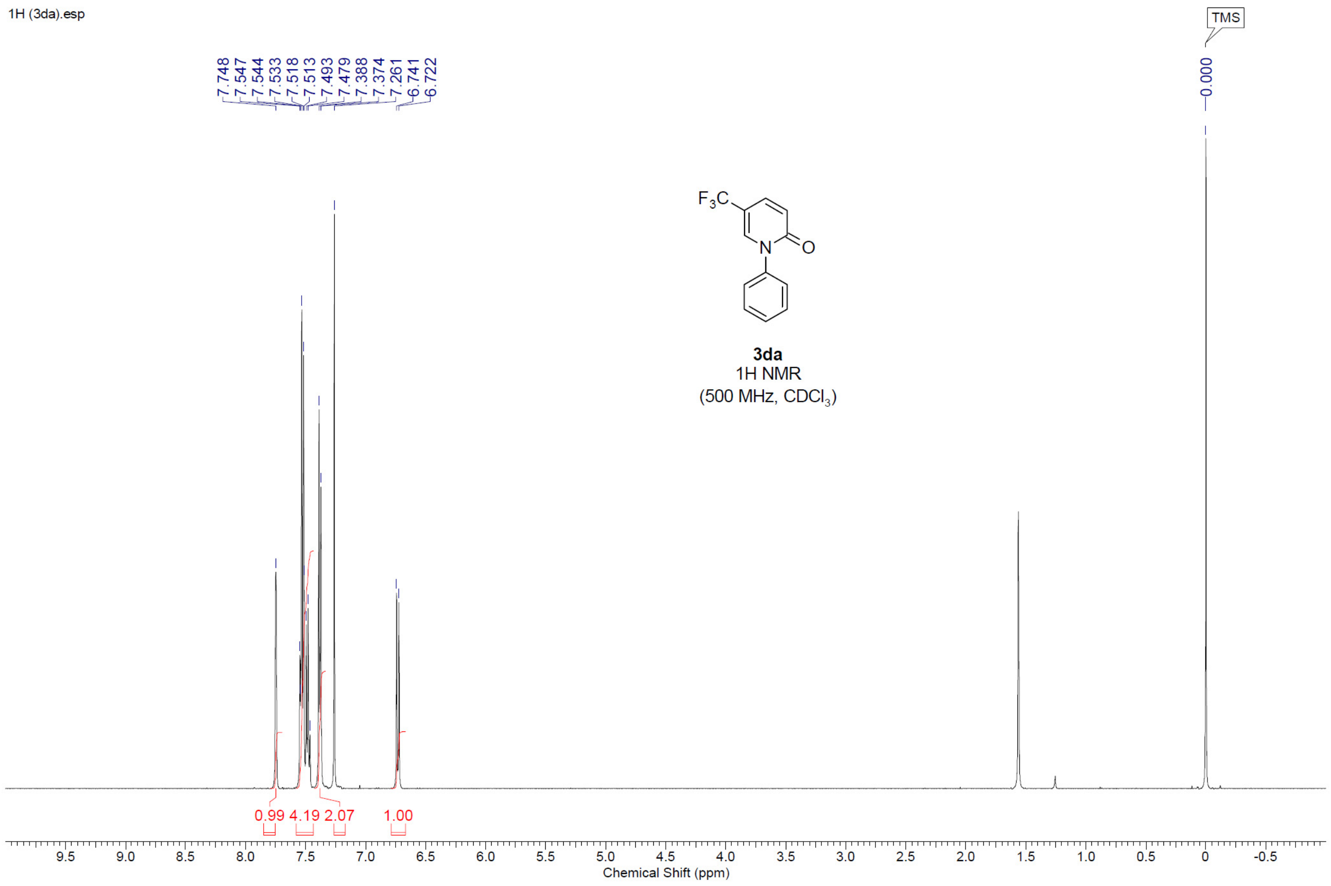
1H (3da).esp
7.748
7.547
7.544
7.533
7.518
7.513
7.493
7.479
7.388
7.374
7.261
6.741
6.722
TMS
0.000
F3C
N
O
3da
1H NMR
(500 MHz, CDCl3)
0.99 4.19 2.07 1.00
Chemical Shift (ppm)

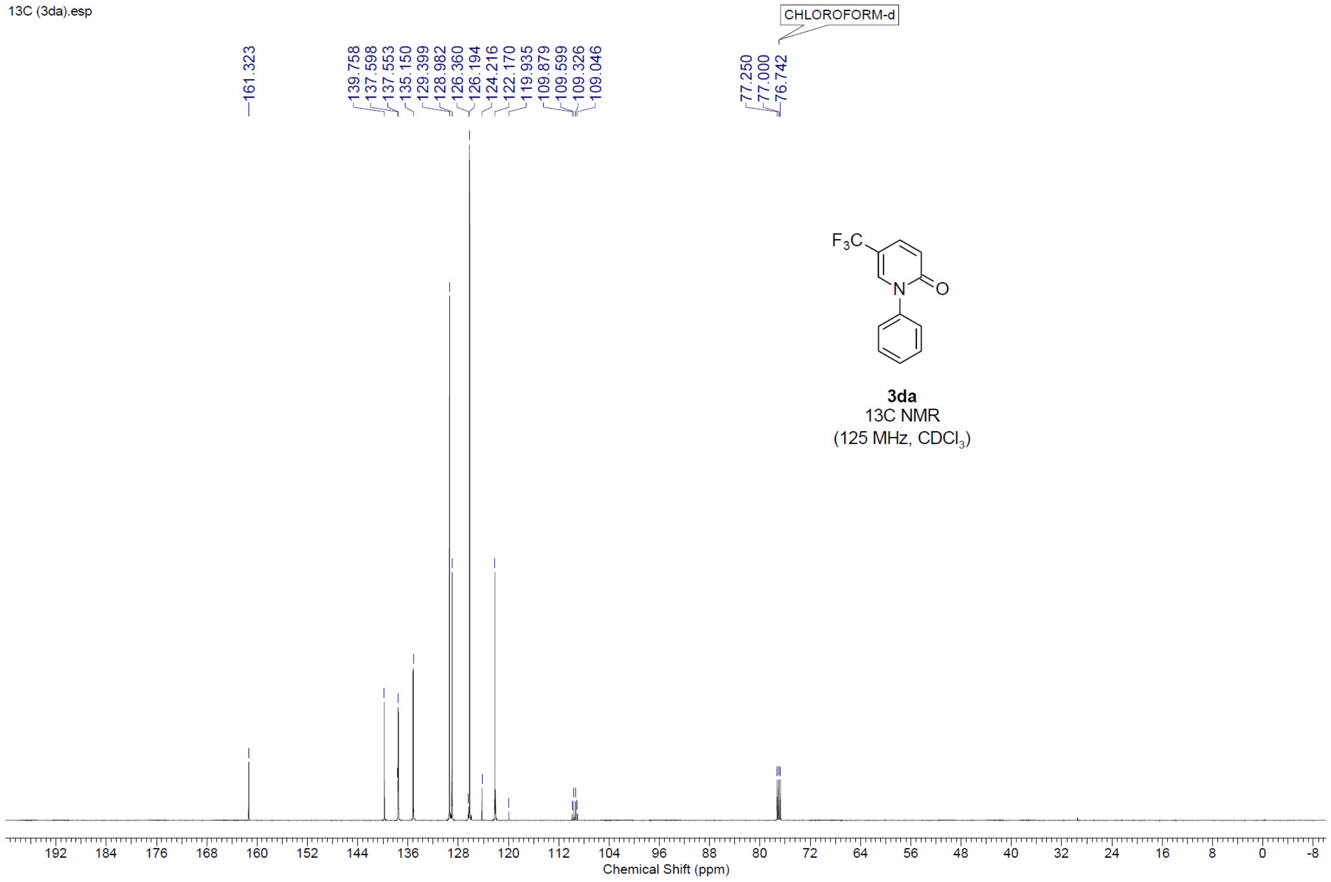
13C (3da).esp
161.323
139.758
137.598
137.553
135.150
129.399
128.982
126.360
126.194
124.216
122.170
119.935
109.879
109.599
109.326
109.046
77.250
77.000
76.742
CHLOROFORM-d
F3C
N
O
3da
13C NMR
(125 MHz, CDCl3)
Chemical Shift (ppm)

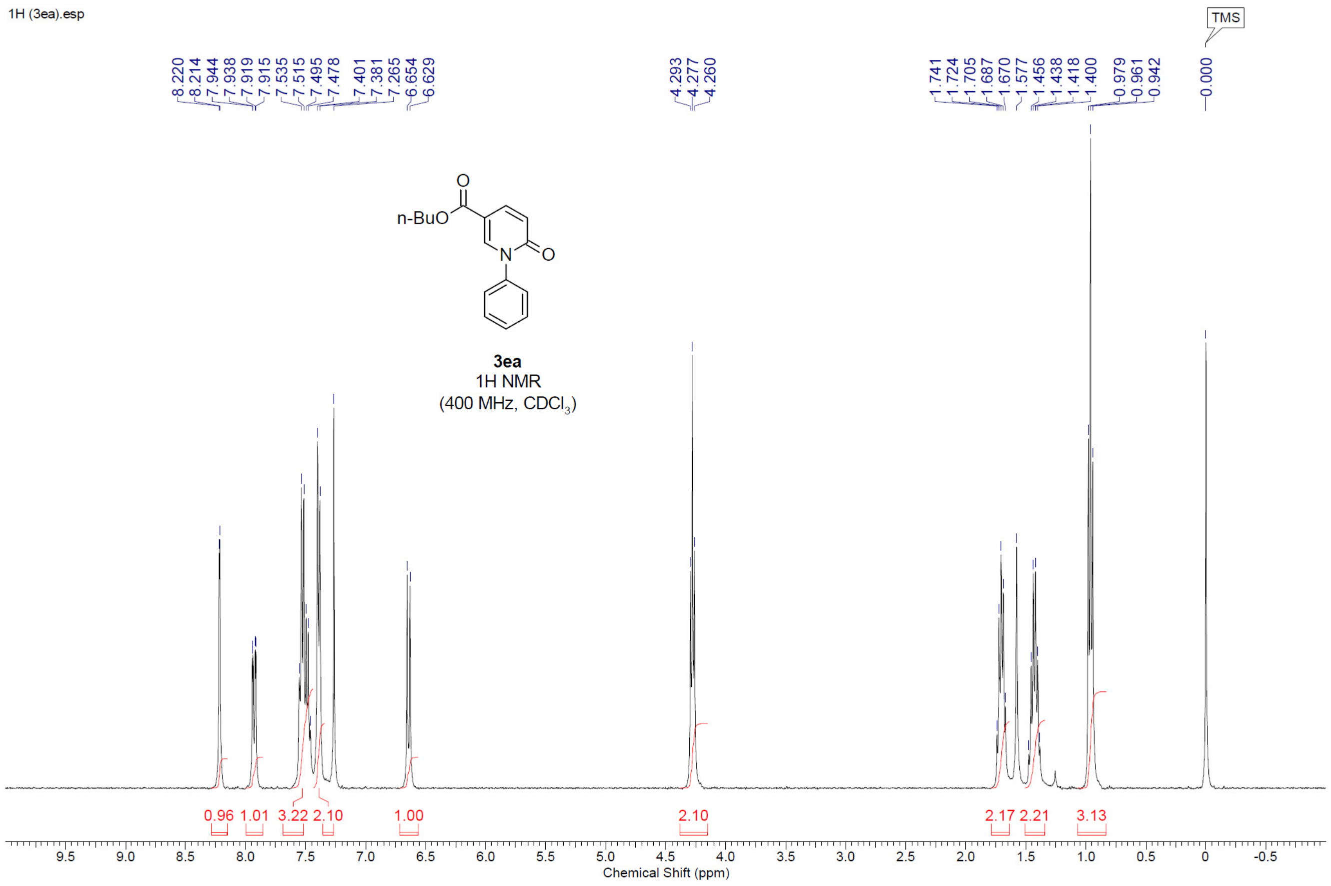
1H (3ea).esp
TMS
8.220
8.214
7.944
7.938
7.919
7.915
7.535
7.515
7.495
7.478
7.401
7.381
7.265
6.654
6.629
4.293
4.277
4.260
1.741
1.724
1.705
1.687
1.670
1.577
1.456
1.438
1.418
1.400
0.979
0.961
0.942
0.000
n-BuO
N
O
3ea
1H NMR
(400 MHz, CDCl3)
0.96
1.01
3.22
2.10
1.00
2.10
2.17
2.21
3.13
Chemical Shift (ppm)

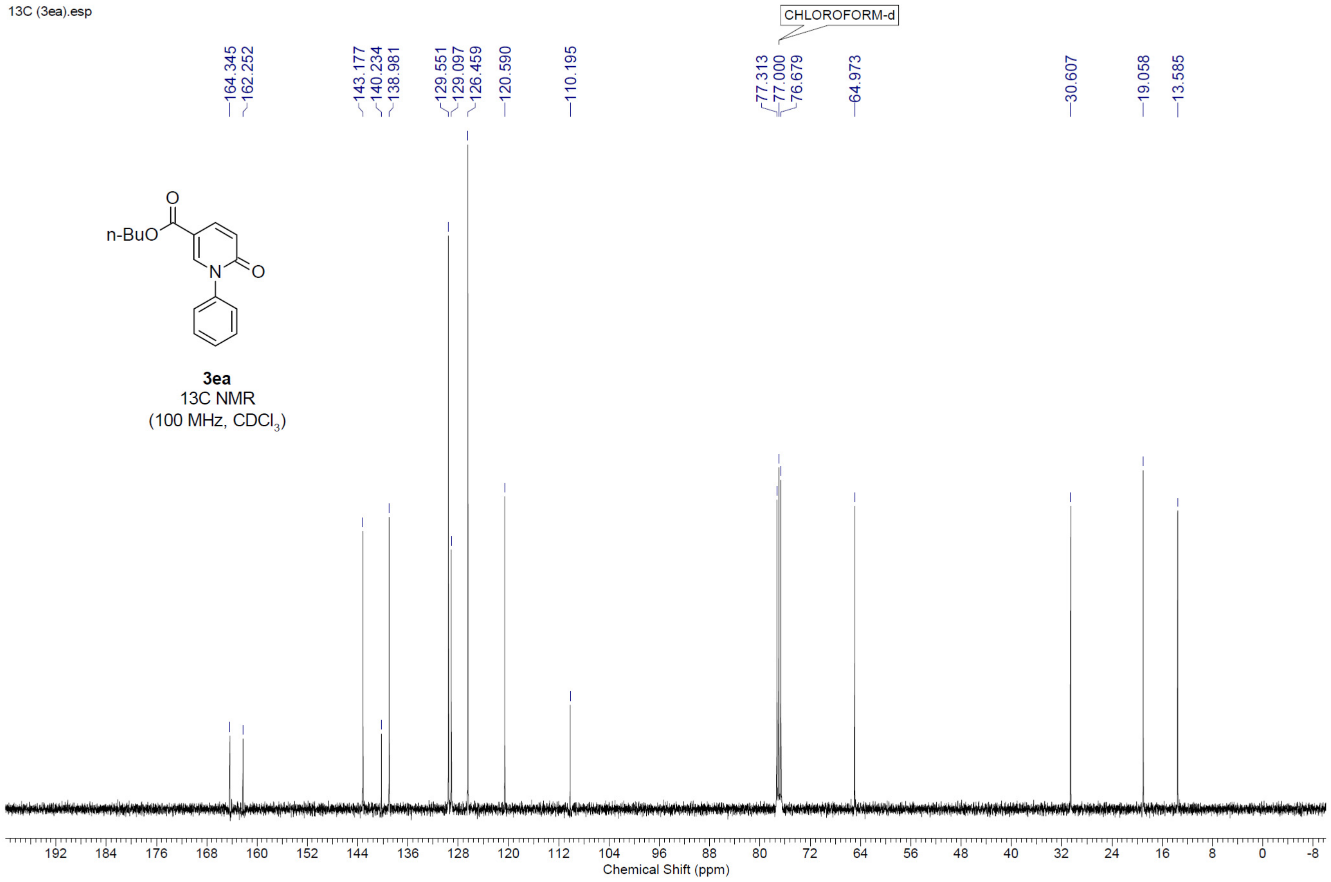
13C (3ea).esp
CHLOROFORM-d
164.345
162.252
143.177
140.234
138.981
129.551
129.097
126.459
120.590
110.195
77.313
77.000
76.679
64.973
30.607
19.058
13.585
n-BuO
N
O
3ea
13C NMR
(100 MHz, CDCl3)
Chemical Shift (ppm)

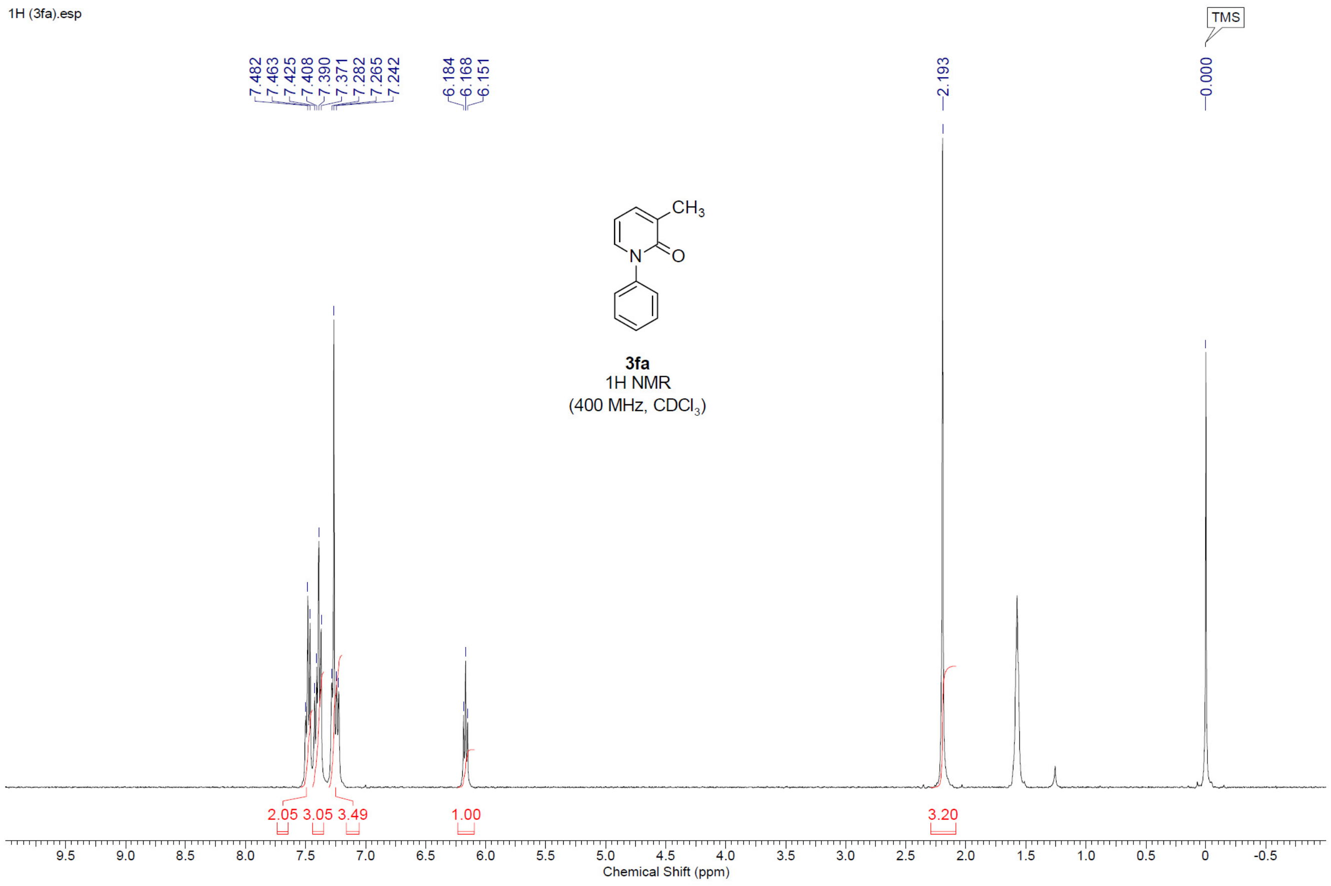
1H (3fa).esp
TMS
7.482
7.463
7.425
7.408
7.390
7.371
7.282
7.265
7.242
6.184
6.168
6.151
2.193
0.000
CH3
N
O
3fa
1H NMR
(400 MHz, CDCl3)
2.05
3.05
3.49
1.00
3.20
Chemical Shift (ppm)

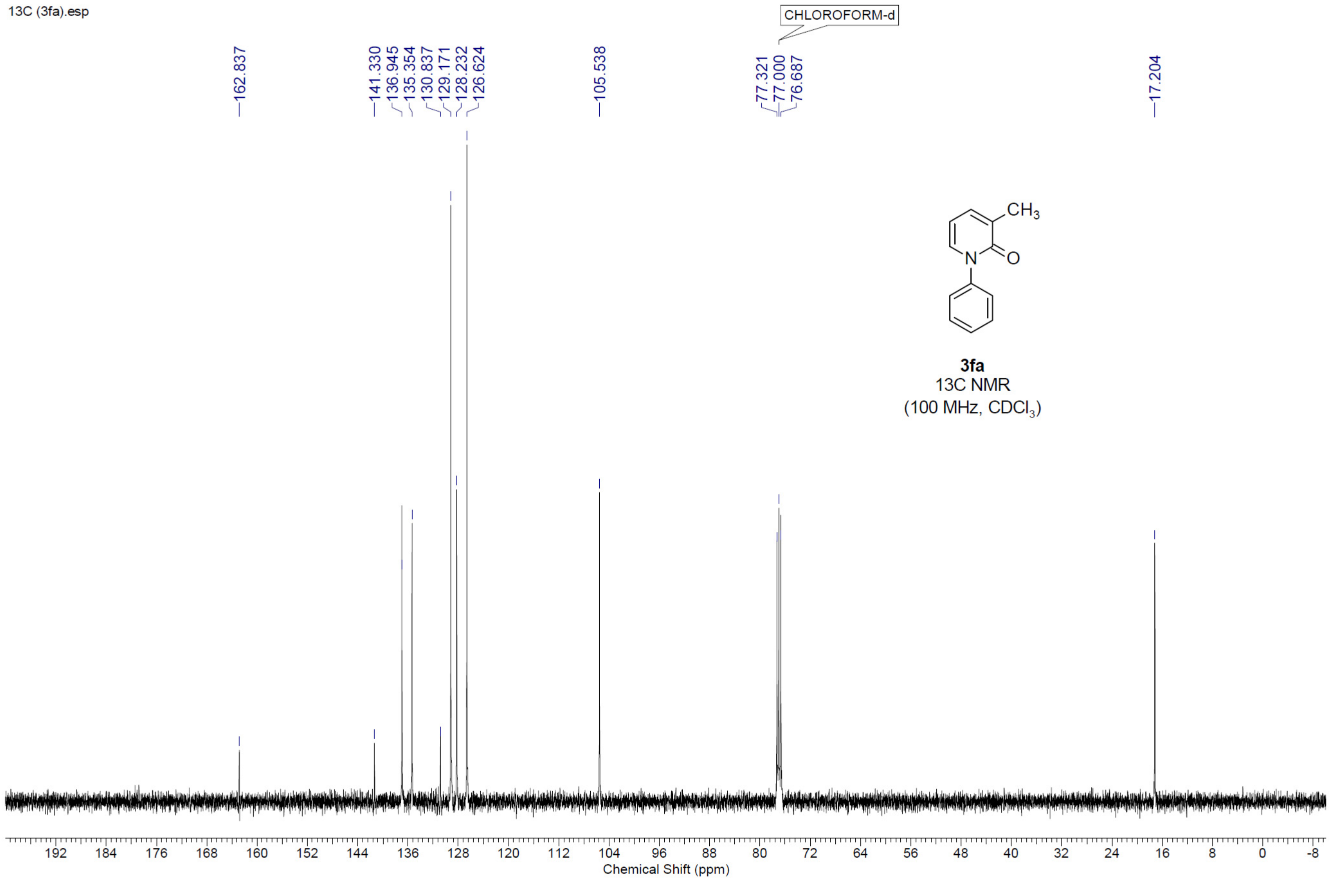
13C (3fa).esp
CHLOROFORM-d
162.837
141.330
136.945
135.354
130.837
129.171
128.232
126.624
105.538
77.321
77.000
76.687
17.204
CH3
N
O
3fa
13C NMR
(100 MHz, CDCl3)
Chemical Shift (ppm)

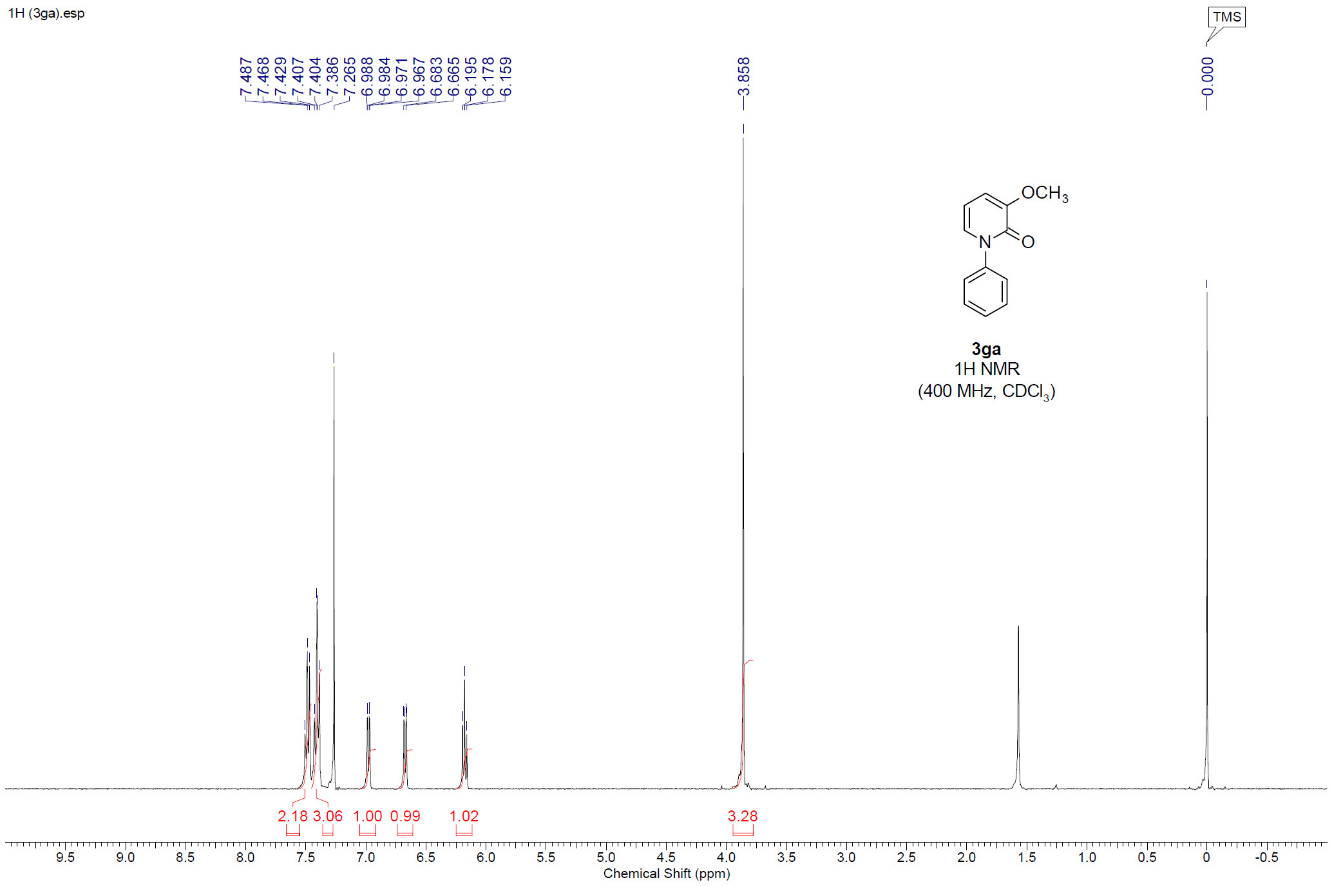
1H (3ga).esp
TMS
7.487
7.468
7.429
7.407
7.404
7.386
7.265
6.988
6.984
6.971
6.967
6.683
6.665
6.195
6.178
6.159
3.858
0.000
OCH3
N
O
3ga
1H NMR
(400 MHz, CDCl3)
2.18
3.06
1.00
0.99
1.02
3.28
Chemical Shift (ppm)

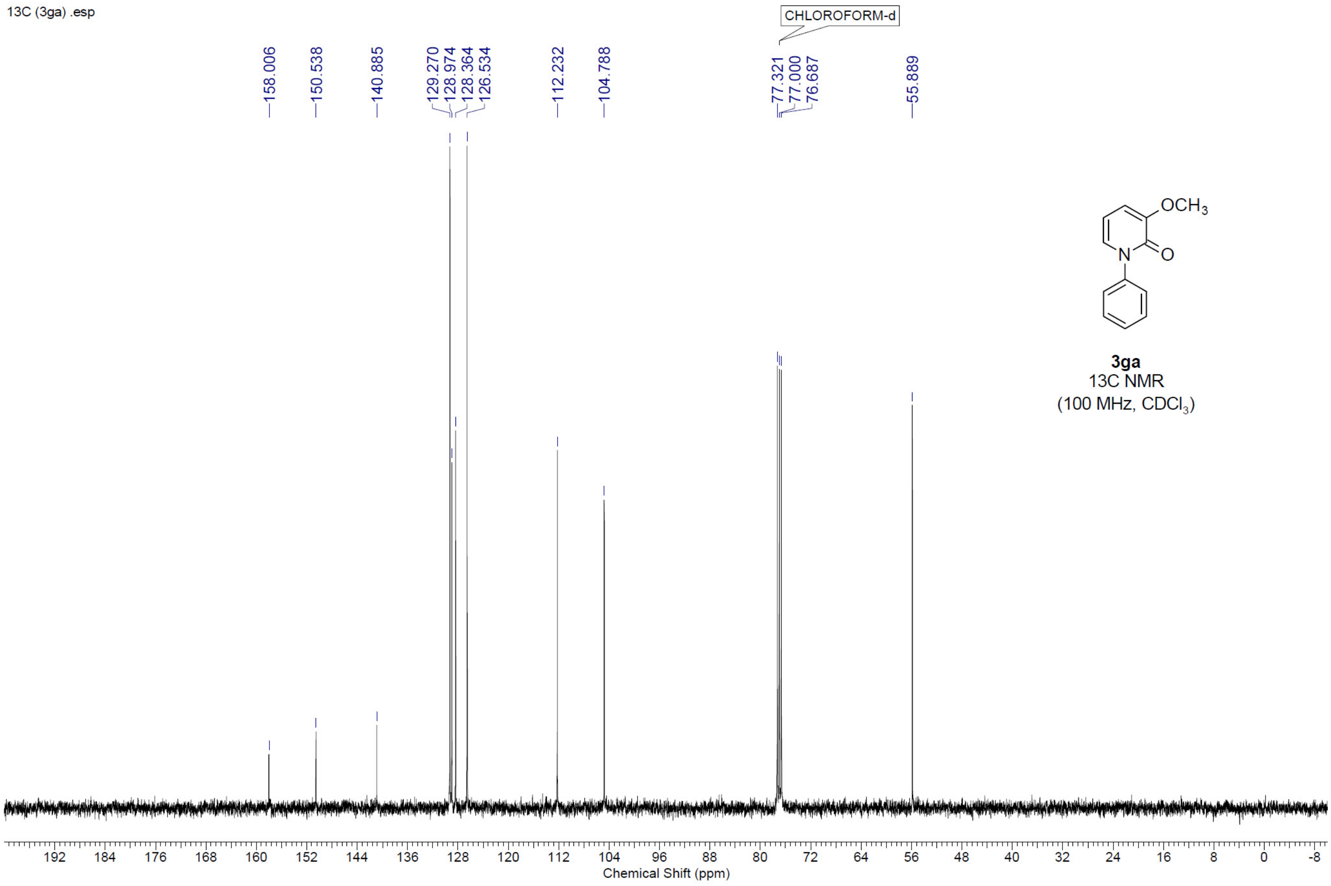
13C (3ga) .esp
CHLOROFORM-d
158.006
150.538
140.885
129.270
128.974
128.364
126.534
112.232
104.788
77.321
77.000
76.687
55.889
OCH3
N
O
3ga
13C NMR
(100 MHz, CDCl3)
Chemical Shift (ppm)

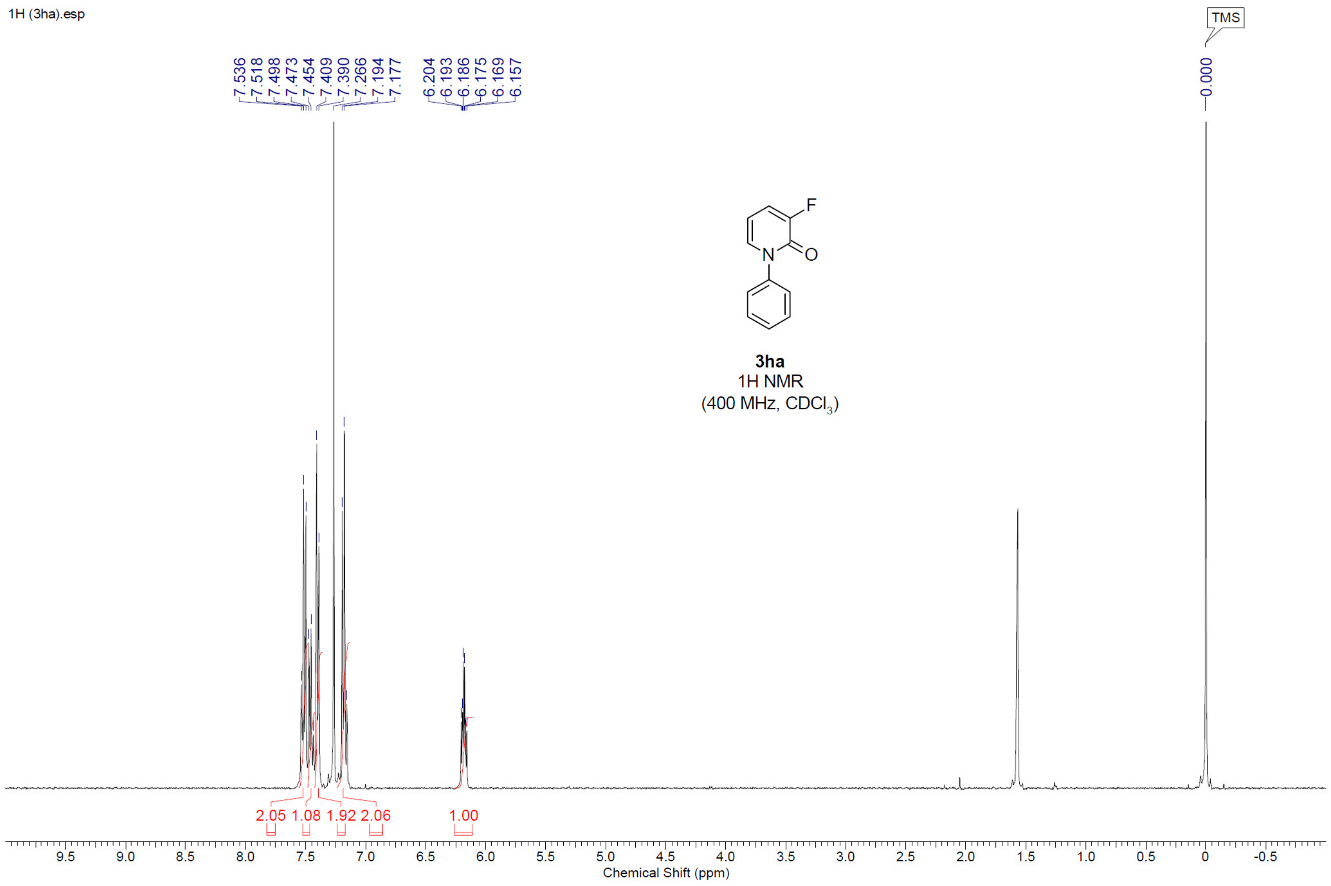
1H (3ha).esp
TMS
7.536
7.518
7.498
7.473
7.454
7.409
7.390
7.266
7.194
7.177
6.204
6.193
6.186
6.175
6.169
6.157
-0.000
3ha
1H NMR
(400 MHz, CDCl3)
2.05
1.08
1.92
2.06
1.00
Chemical Shift (ppm)

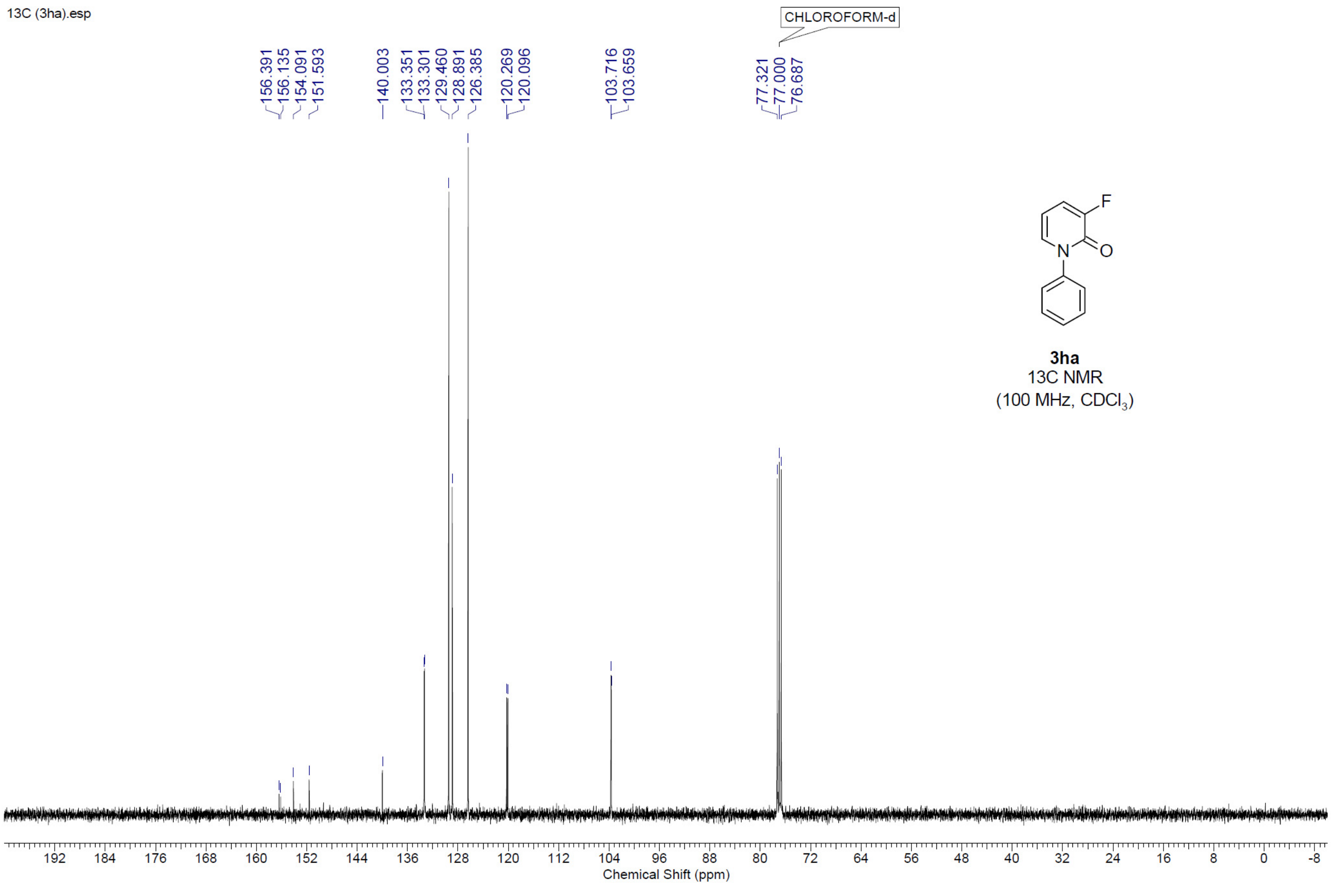
13C (3ha).esp
CHLOROFORM-d
156.391
156.135
154.091
151.593
140.003
133.351
133.301
129.460
128.891
126.385
120.269
120.096
103.716
103.659
77.321
77.000
76.687
3ha
13C NMR
(100 MHz, CDCl3)
Chemical Shift (ppm)

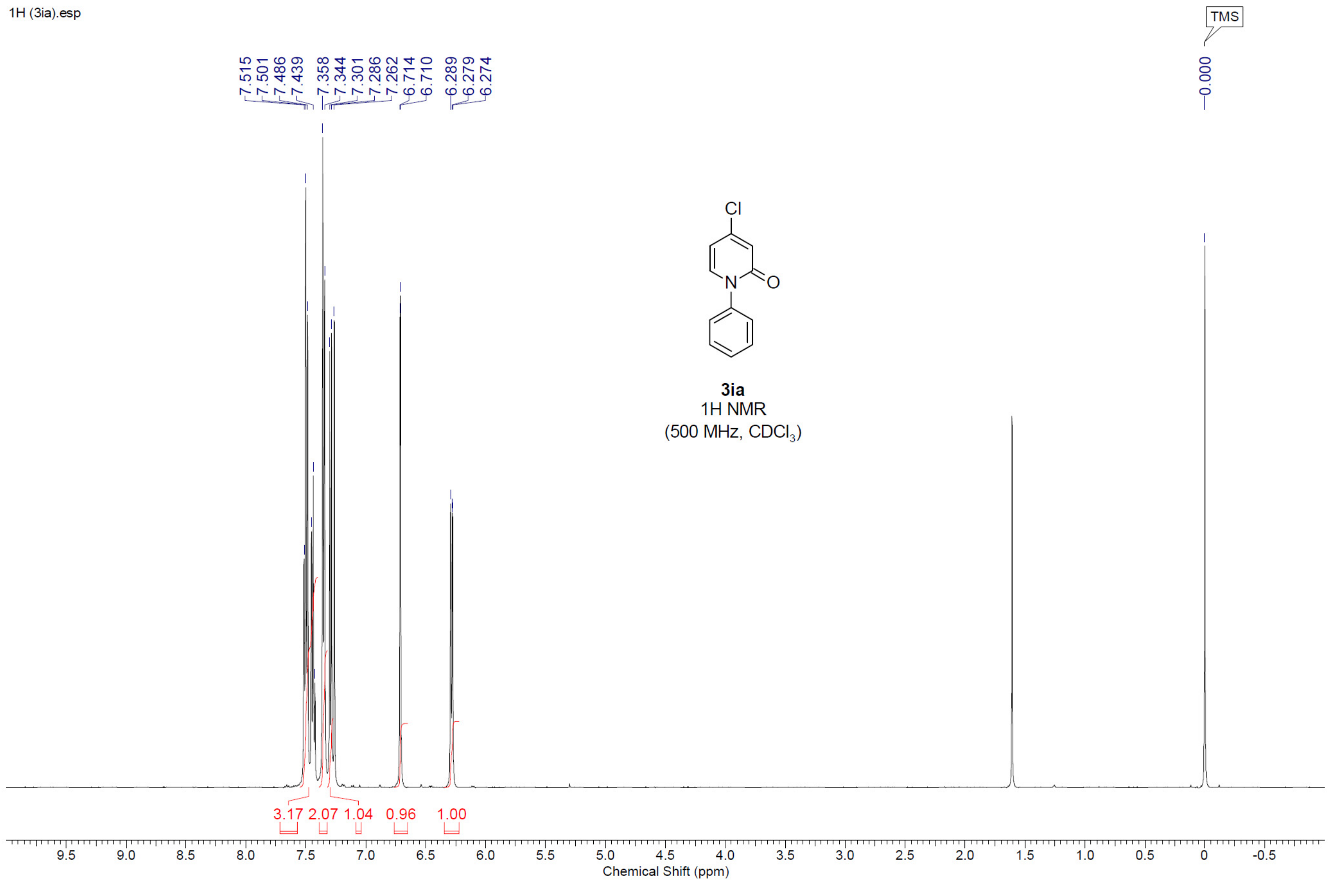
1H (3ia).esp
TMS
7.515
7.501
7.486
7.439
7.358
7.344
7.301
7.286
7.262
6.714
6.710
6.289
6.279
6.274
0.000
Cl
N
O
3ia
1H NMR
(500 MHz, CDCl3)
3.17
2.07
1.04
0.96
1.00
9.5
9.0
8.5
8.0
7.5
7.0
6.5
6.0
5.5
5.0
4.5
4.0
3.5
3.0
2.5
2.0
1.5
1.0
0.5
0
-0.5
Chemical Shift (ppm)

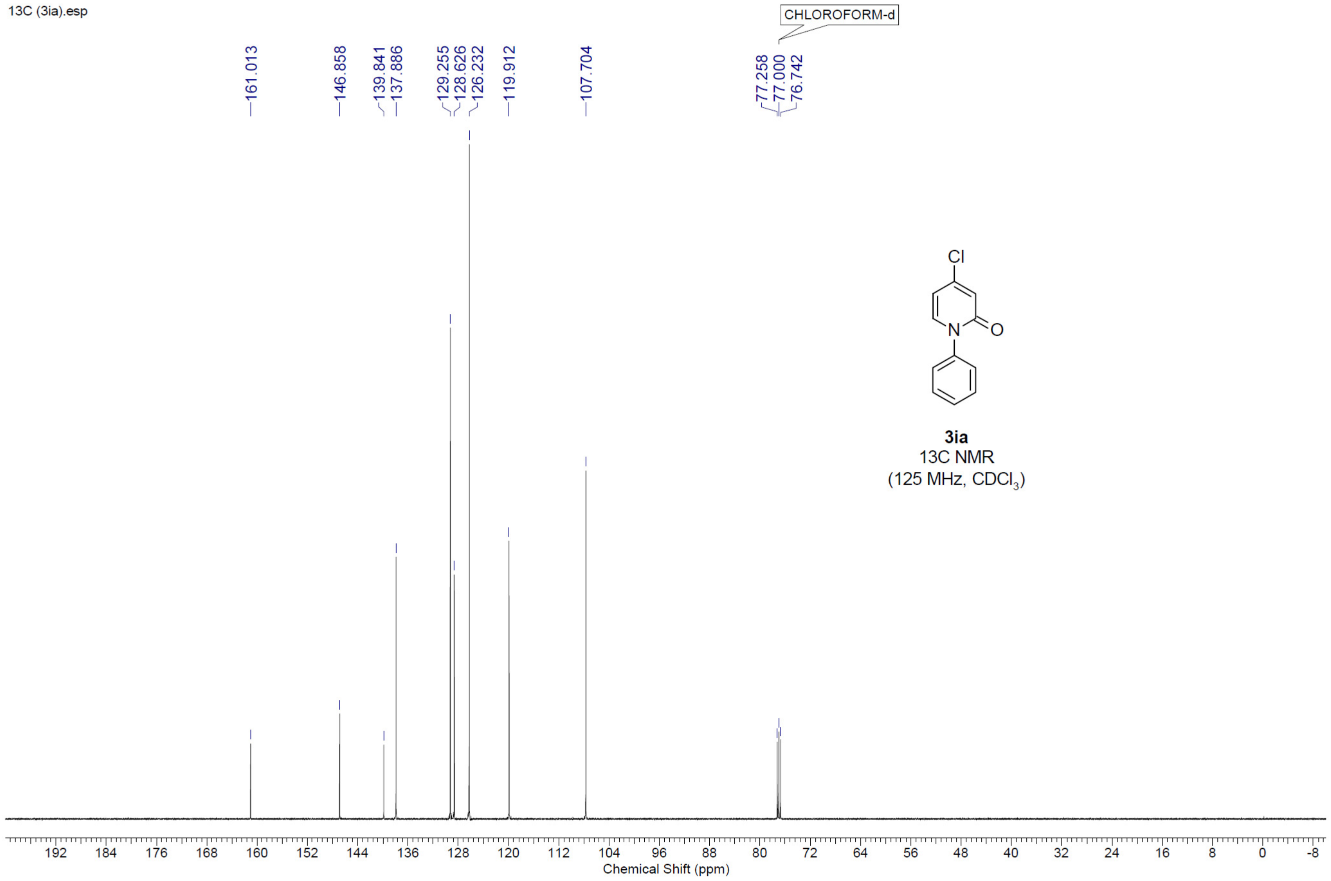
13C (3ia).esp
CHLOROFORM-d
161.013
146.858
139.841
137.886
129.255
128.626
126.232
119.912
107.704
77.258
77.000
76.742
Cl
N
O
3ia
13C NMR
(125 MHz, CDCl3)
192
184
176
168
160
152
144
136
128
120
112
104
96
88
80
72
64
56
48
40
32
24
16
8
0
-8
Chemical Shift (ppm)

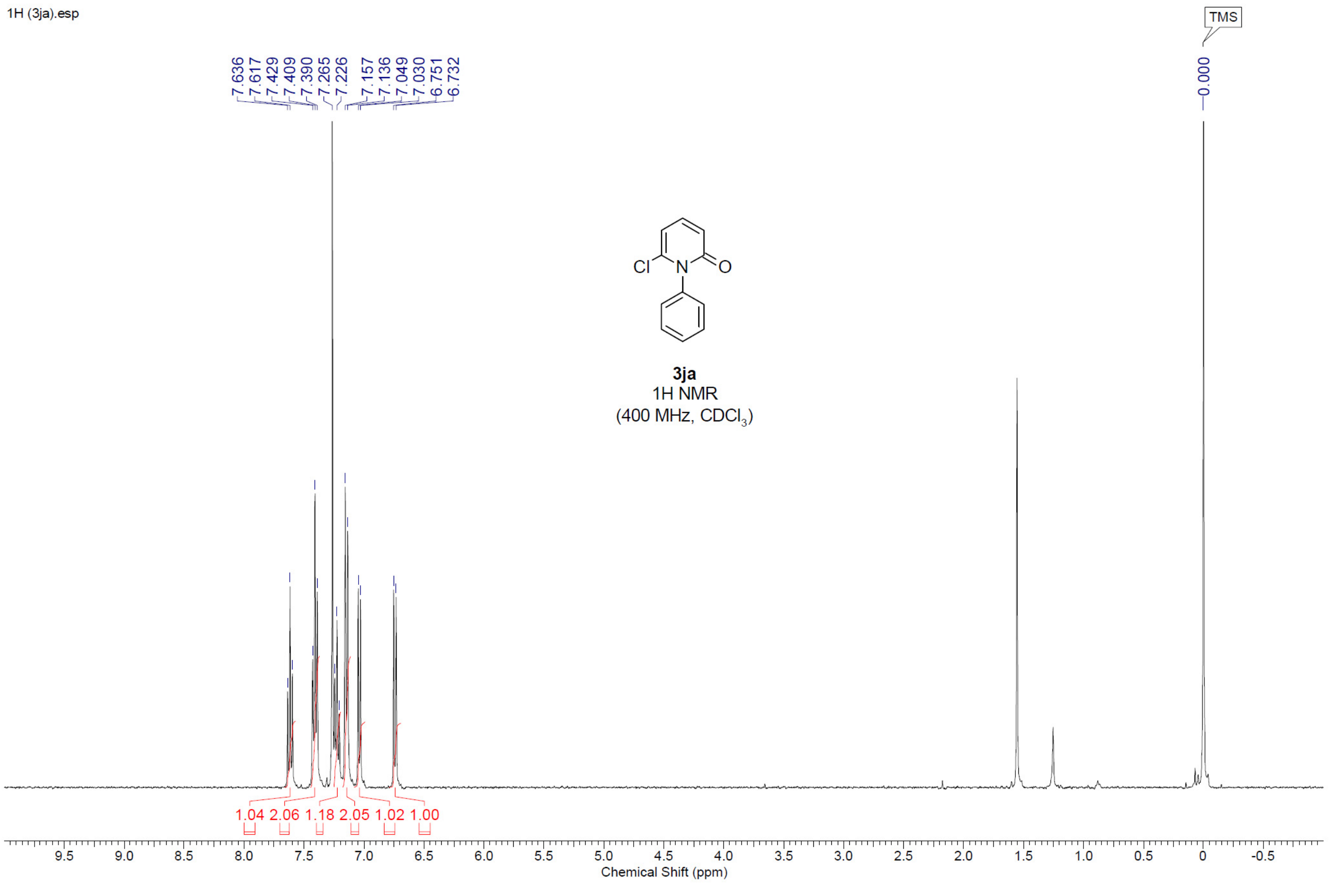
1H (3ja).esp
7.636
7.617
7.429
7.409
7.390
7.265
7.226
7.157
7.136
7.049
7.030
6.751
6.732
TMS
0.000
Cl
N
O
3ja
1H NMR
(400 MHz, CDCl3)
1.04 2.06 1.18 2.05 1.02 1.00
Chemical Shift (ppm)

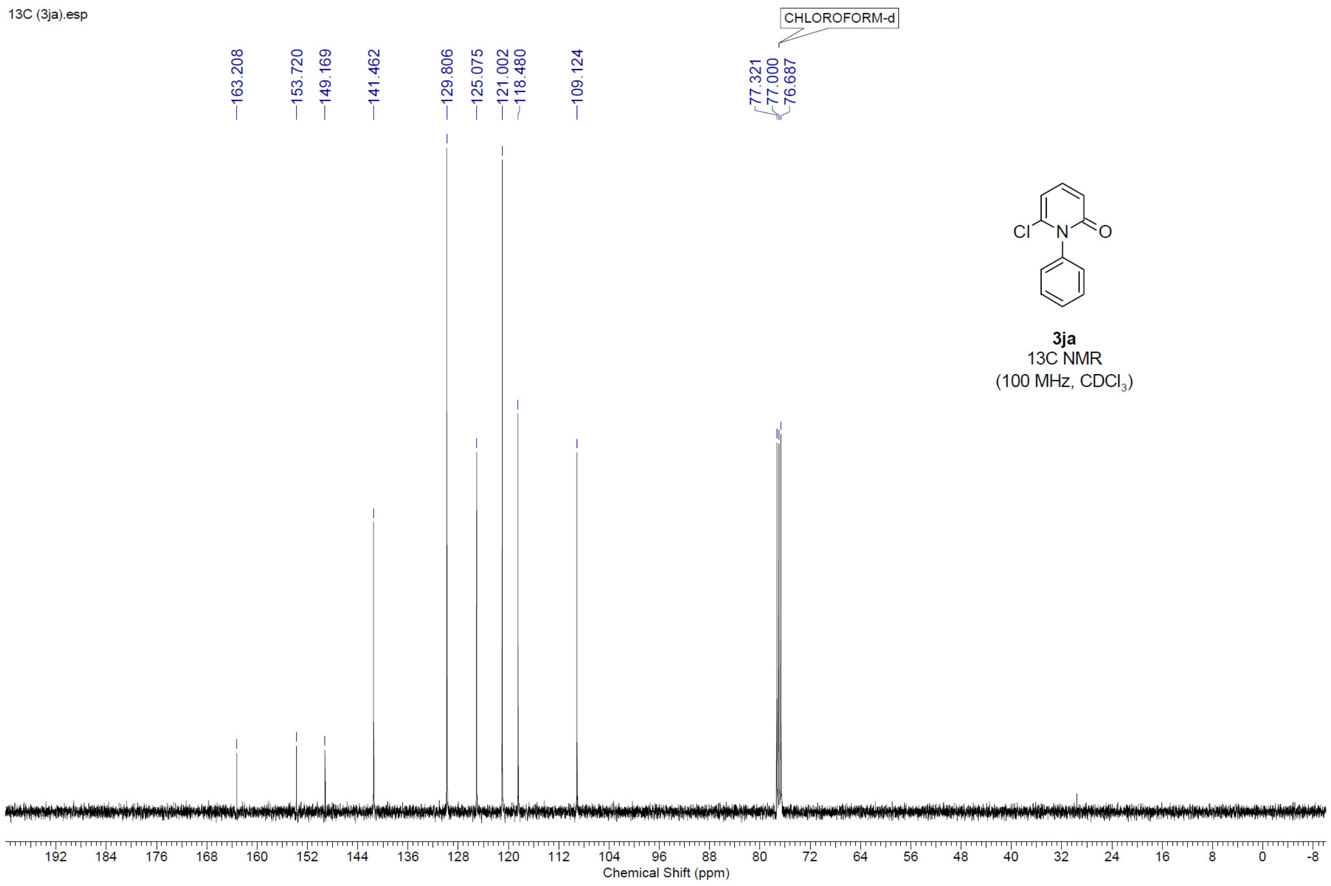
13C (3ja).esp
163.208
153.720
149.169
141.462
129.806
125.075
121.002
118.480
109.124
CHLOROFORM-d
77.321
77.000
76.687
Cl
N
O
3ja
13C NMR
(100 MHz, CDCl3)
Chemical Shift (ppm)

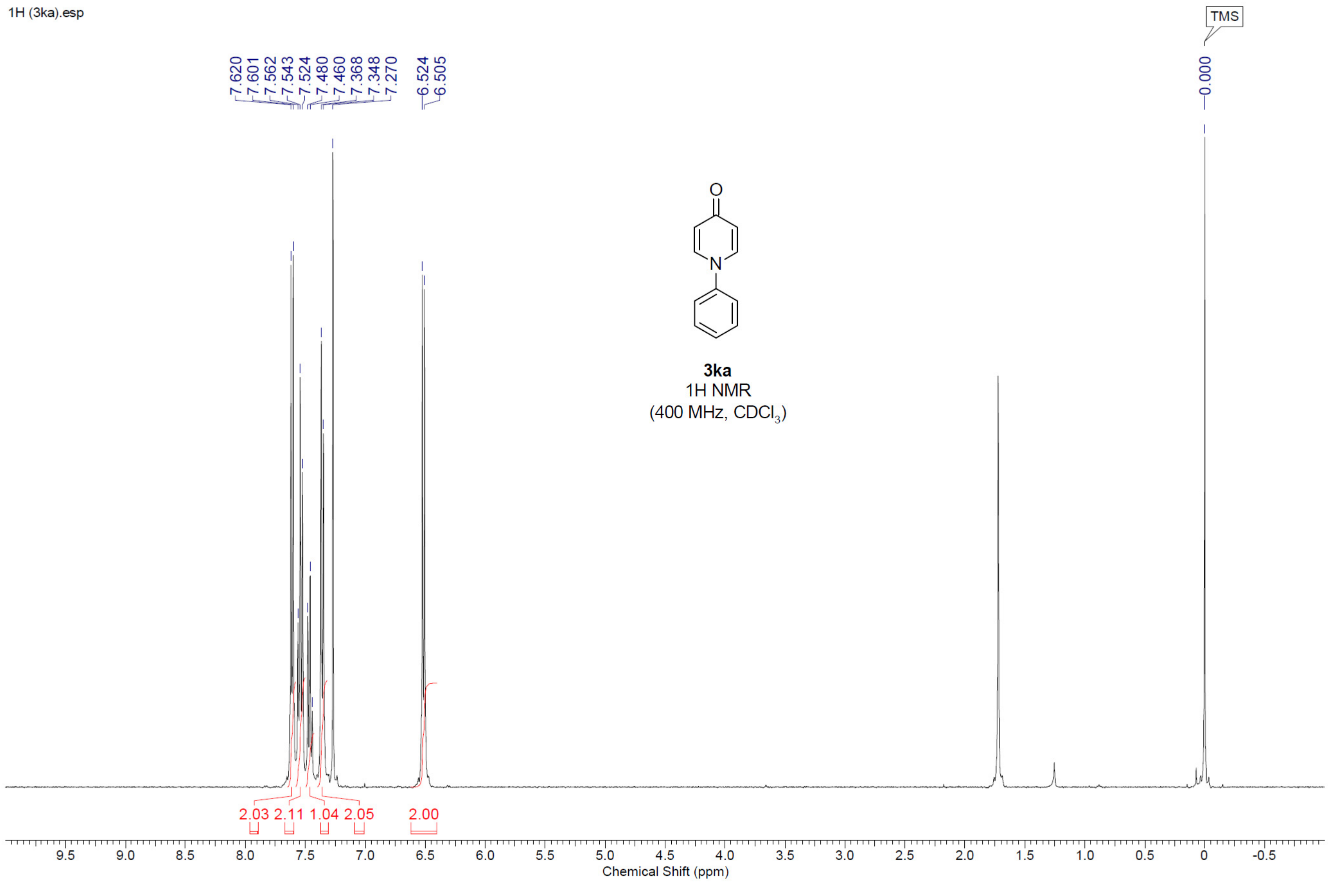
1H (3ka).esp
7.620
7.601
7.562
7.543
7.524
7.480
7.460
7.368
7.348
7.270
6.524
6.505
TMS
0.000
O
N
3ka
1H NMR
(400 MHz, CDCl3)
2.03
2.11
1.04
2.05
2.00
Chemical Shift (ppm)

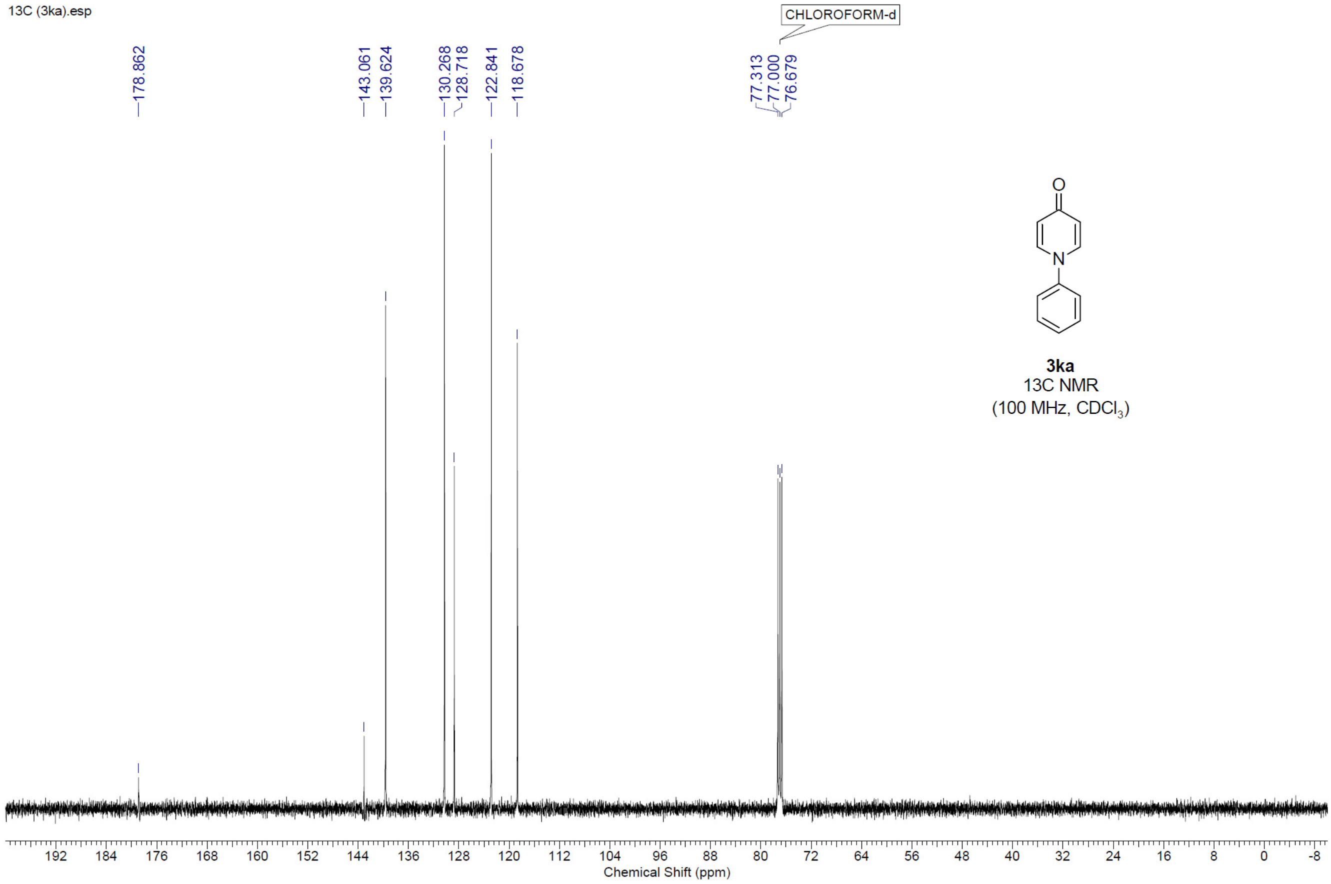
13C (3ka).esp
178.862
143.061
139.624
130.268
128.718
122.841
118.678
CHLOROFORM-d
77.313
77.000
76.679
O
N
3ka
13C NMR
(100 MHz, CDCl3)
Chemical Shift (ppm)

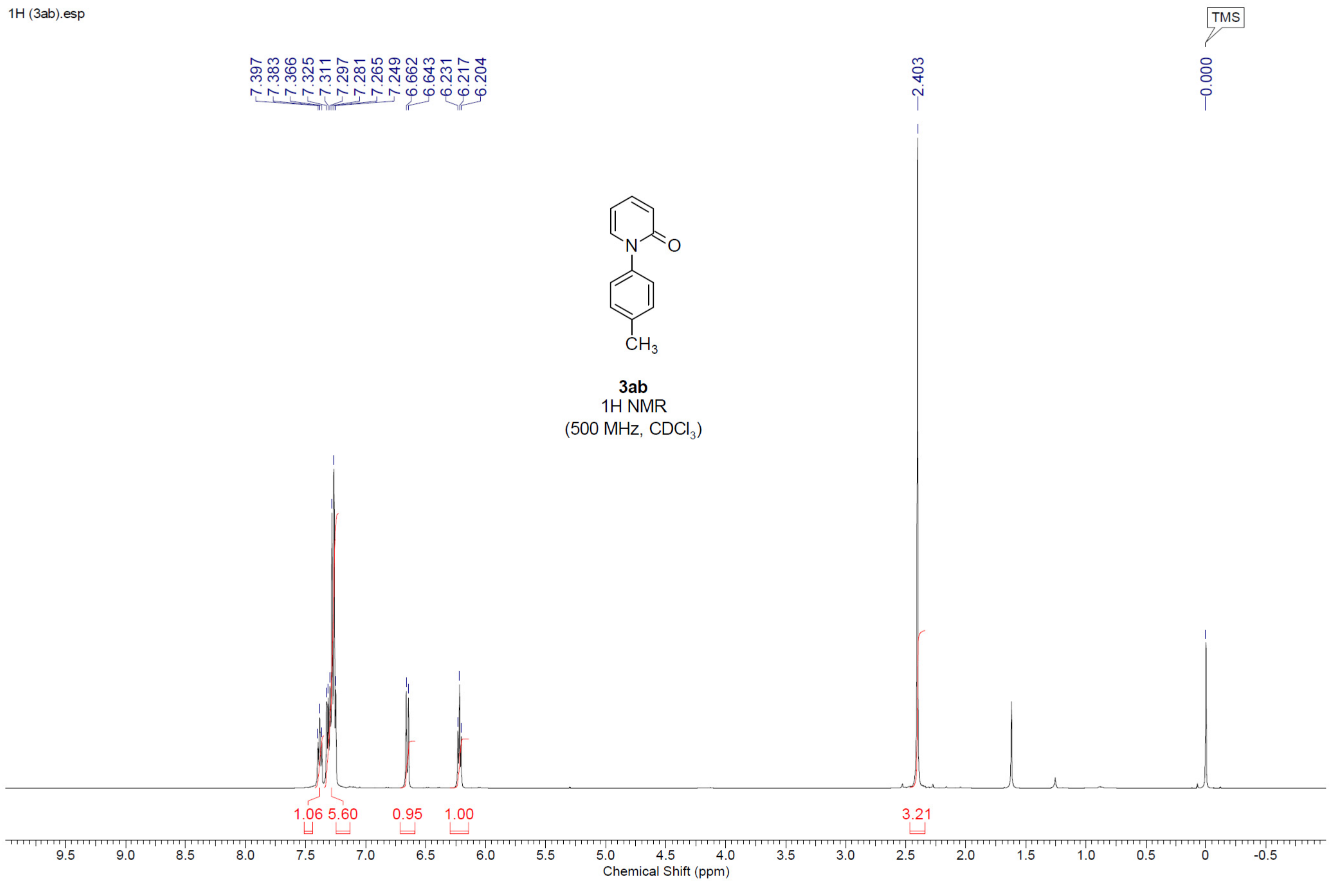
1H (3ab).esp
TMS
7.397
7.383
7.366
7.325
7.311
7.297
7.281
7.265
7.249
6.662
6.643
6.231
6.217
6.204
2.403
0.000
3ab
1H NMR
(500 MHz, CDCl3)
1.06
5.60
0.95
1.00
3.21
Chemical Shift (ppm)

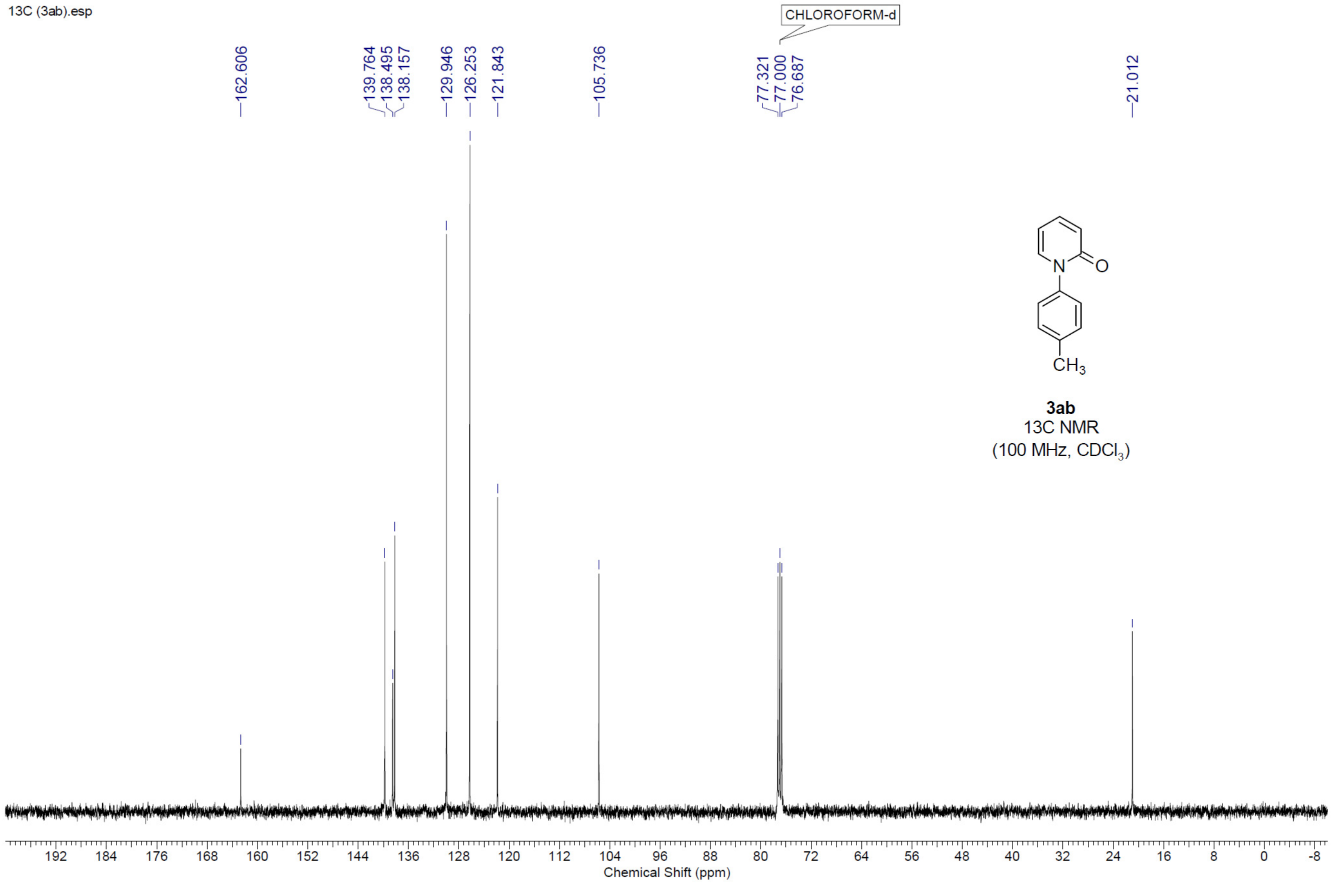
13C (3ab).esp
CHLOROFORM-d
162.606
139.764
138.495
138.157
129.946
126.253
121.843
105.736
77.321
77.000
76.687
21.012
3ab
13C NMR
(100 MHz, CDCl3)
Chemical Shift (ppm)

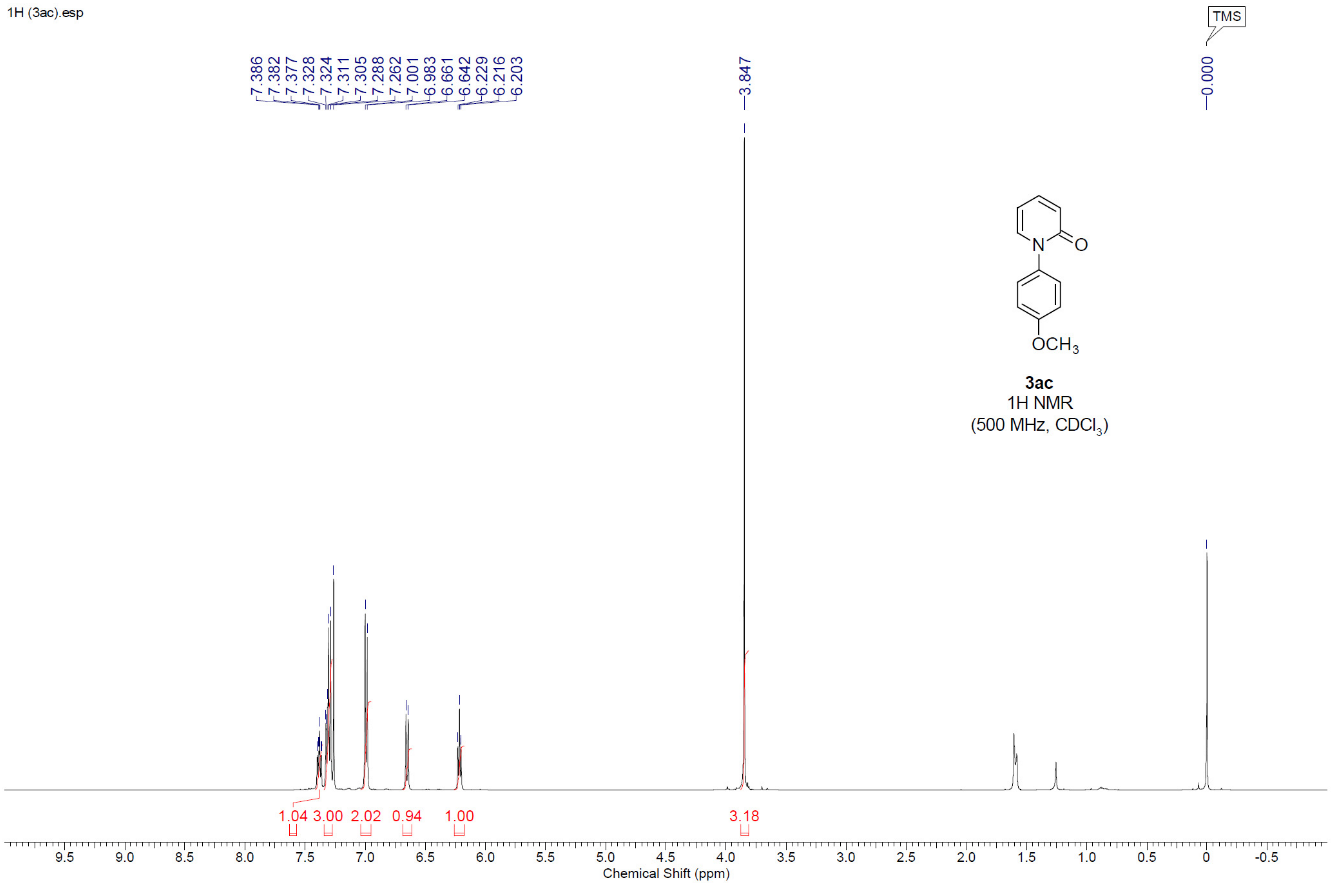
1H (3ac).esp
TMS
7.386
7.382
7.377
7.328
7.324
7.311
7.305
7.288
7.262
7.001
6.983
6.661
6.642
6.229
6.216
6.203
3.847
0.000
OCH3
3ac
1H NMR
(500 MHz, CDCl3)
1.04
3.00
2.02
0.94
1.00
3.18
Chemical Shift (ppm)

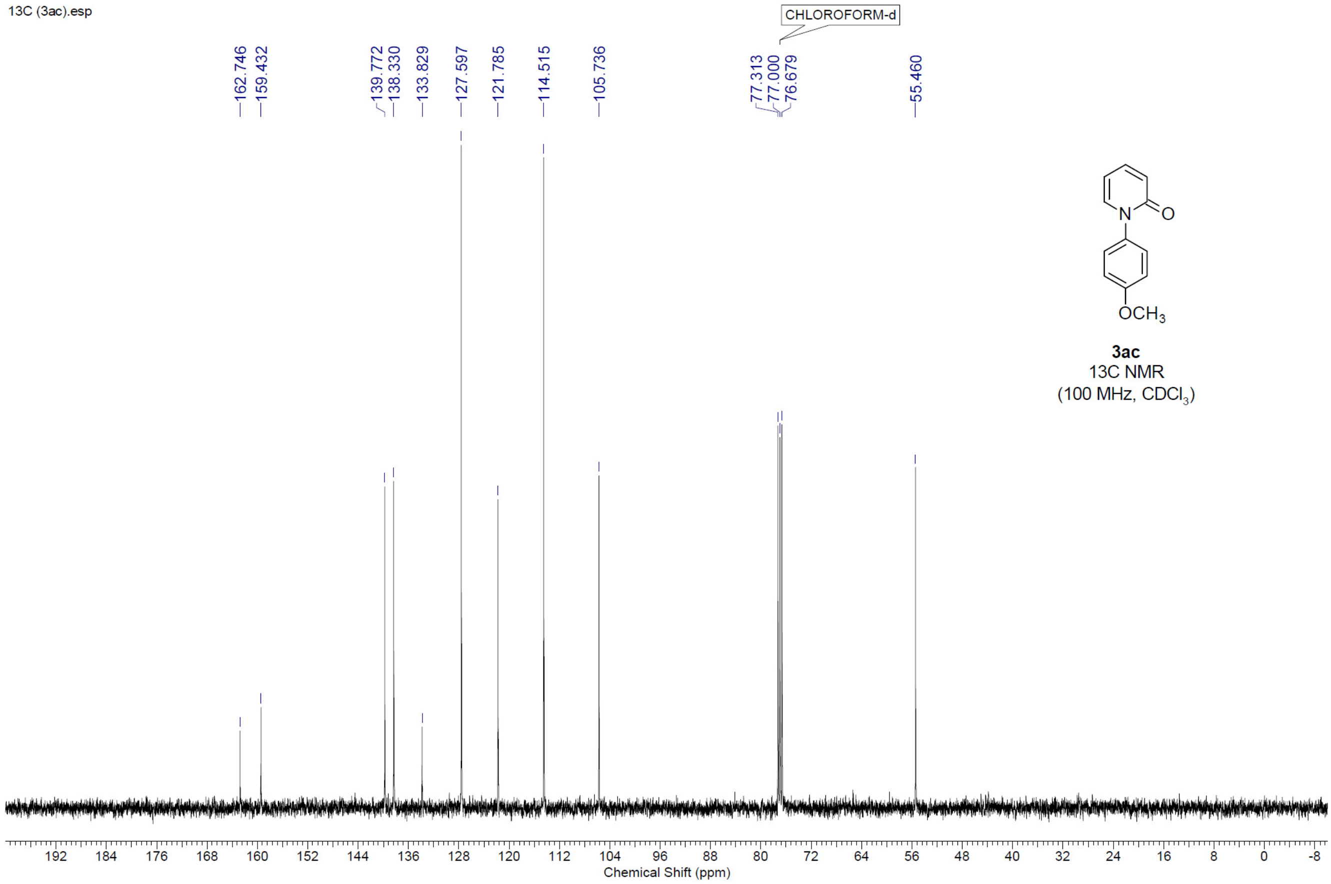
13C (3ac).esp
CHLOROFORM-d
162.746
159.432
139.772
138.330
133.829
127.597
121.785
114.515
105.736
77.313
77.000
76.679
55.460
OCH3
3ac
13C NMR
(100 MHz, CDCl3)
Chemical Shift (ppm)

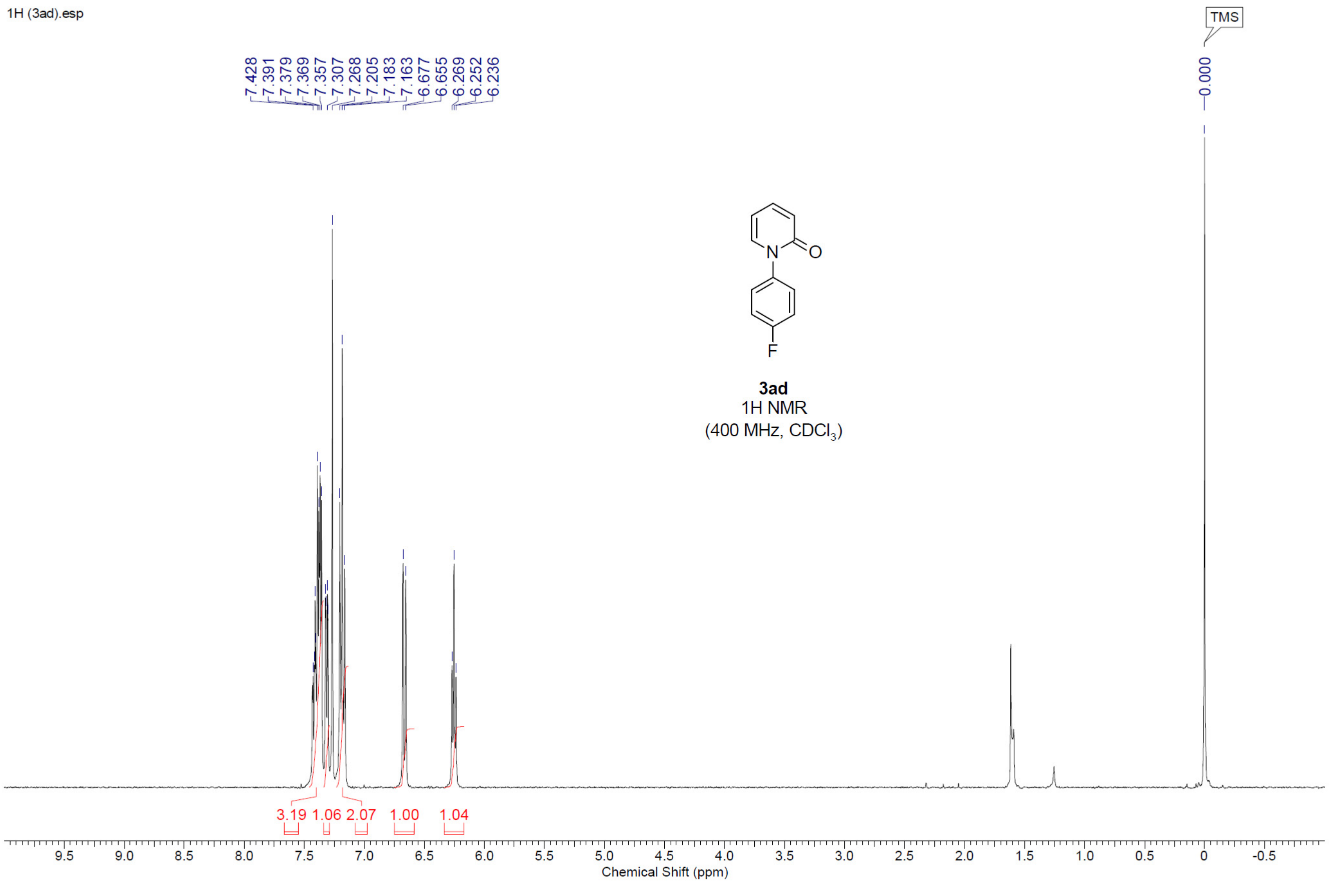
1H (3ad).esp
7.428
7.391
7.379
7.369
7.357
7.307
7.268
7.205
7.183
7.163
6.677
6.655
6.269
6.252
6.236
TMS
0.000
3ad
1H NMR
(400 MHz, CDCl3)
3.19 1.06 2.07 1.00 1.04
Chemical Shift (ppm)

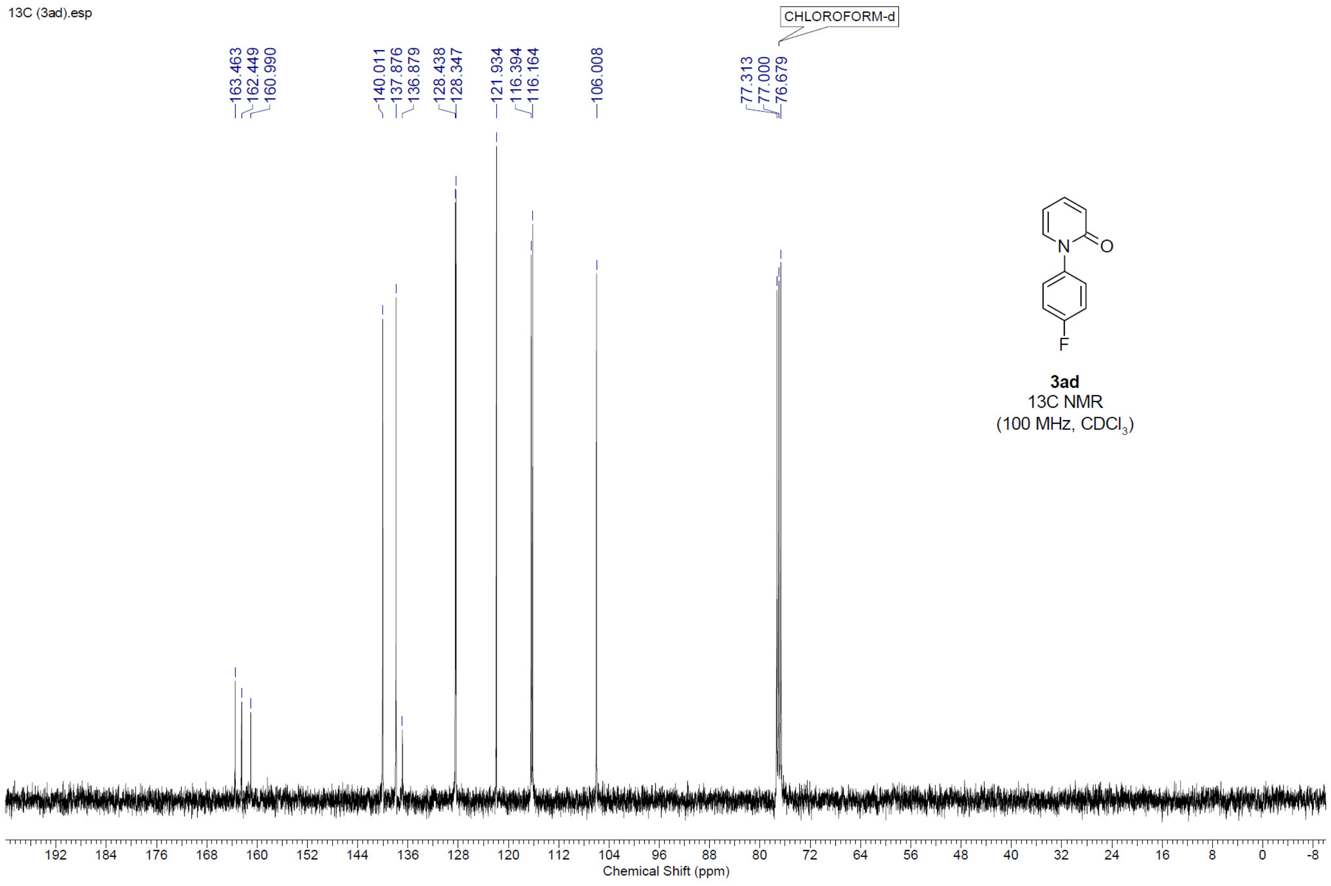
13C (3ad).esp
163.463
162.449
160.990
140.011
137.876
136.879
128.438
128.347
121.934
116.394
116.164
106.008
77.313
77.000
76.679
CHLOROFORM-d
3ad
13C NMR
(100 MHz, CDCl3)
Chemical Shift (ppm)

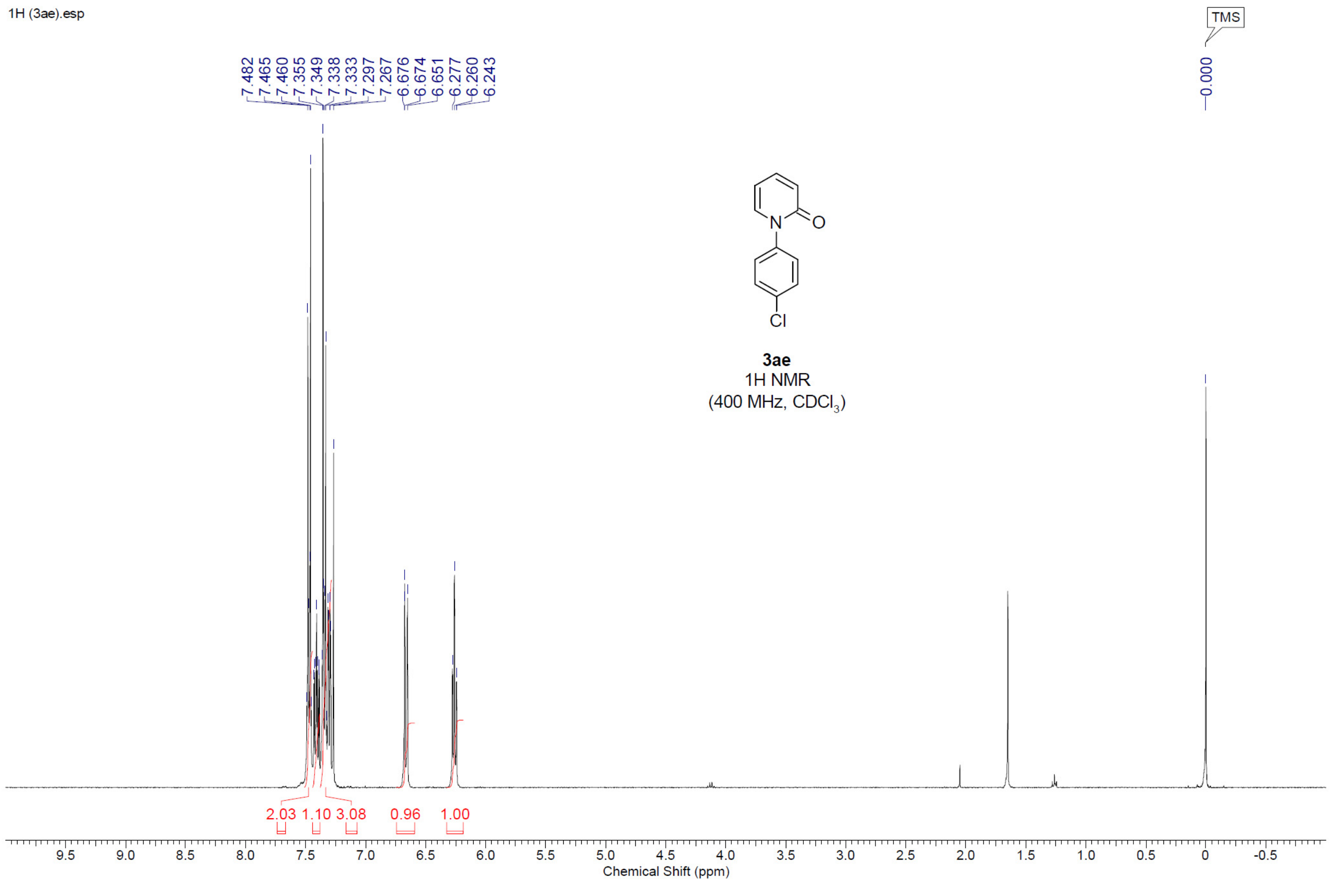
1H (3ae).esp
TMS
7.482
7.465
7.460
7.355
7.349
7.338
7.333
7.297
7.267
6.676
6.674
6.651
6.277
6.260
6.243
0.000
N
O
Cl
3ae
1H NMR
(400 MHz, CDCl3)
2.03
1.10
3.08
0.96
1.00
9.5 9.0 8.5 8.0 7.5 7.0 6.5 6.0 5.5 5.0 4.5 4.0 3.5 3.0 2.5 2.0 1.5 1.0 0.5 0 -0.5
Chemical Shift (ppm)

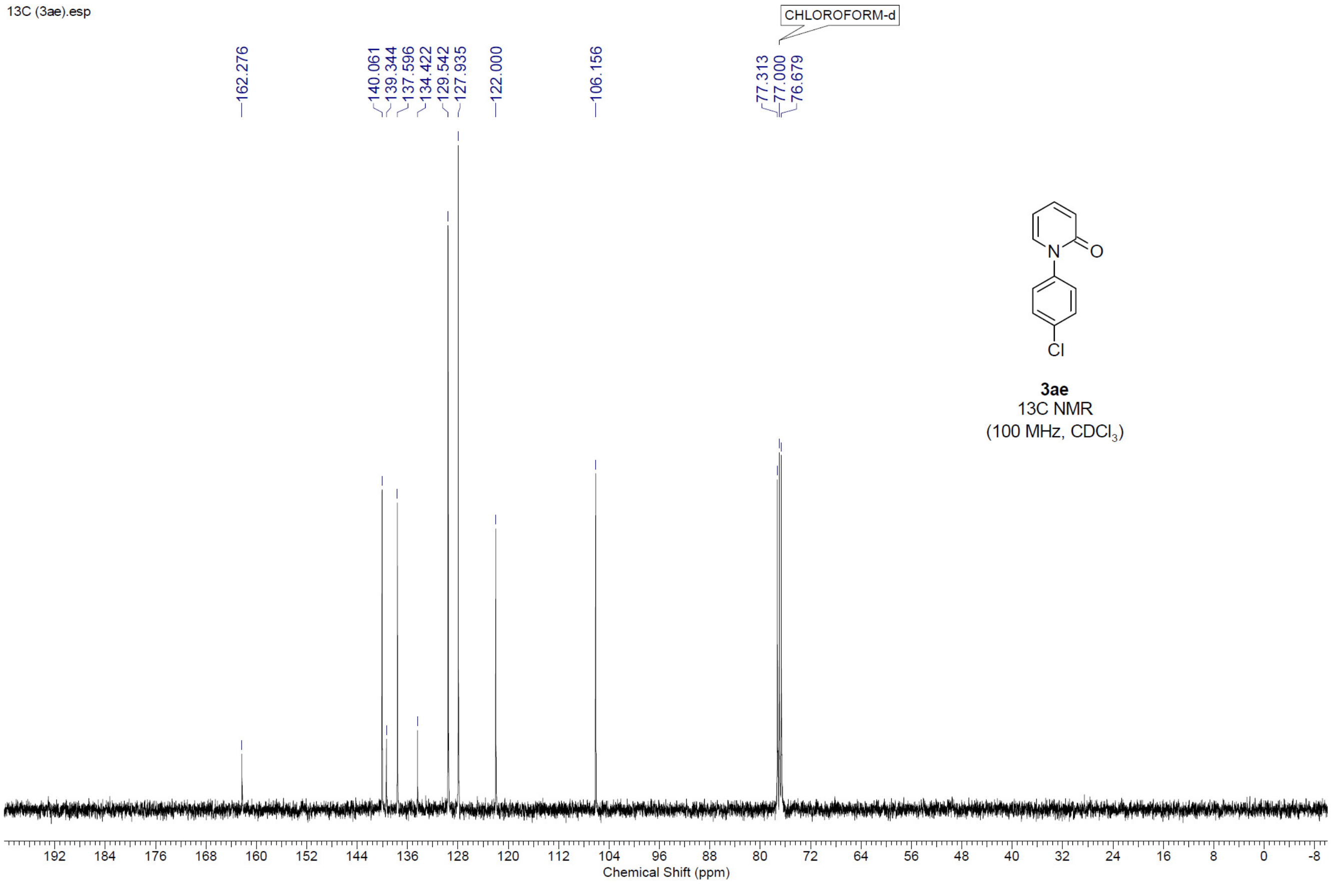
13C (3ae).esp
CHLOROFORM-d
162.276
140.061
139.344
137.596
134.422
129.542
127.935
122.000
106.156
77.313
77.000
76.679
N
O
Cl
3ae
13C NMR
(100 MHz, CDCl3)
192 184 176 168 160 152 144 136 128 120 112 104 96 88 80 72 64 56 48 40 32 24 16 8 0 -8
Chemical Shift (ppm)

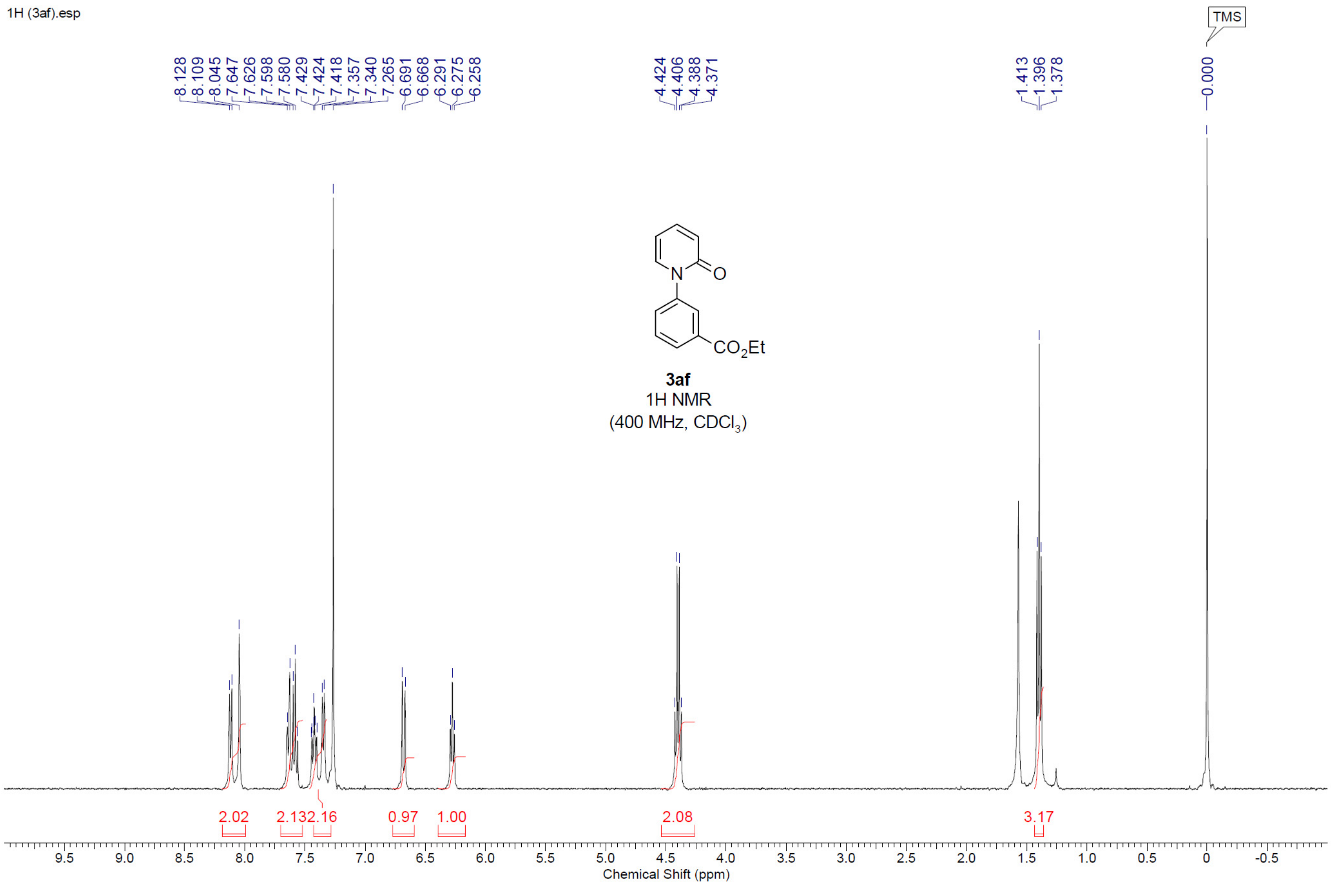
1H (3af).esp
TMS
8.128
8.109
8.045
7.647
7.626
7.598
7.580
7.429
7.424
7.418
7.357
7.340
7.265
6.691
6.668
6.291
6.275
6.258
4.424
4.406
4.388
4.371
1.413
1.396
1.378
0.000
N
O
CO2Et
3af
1H NMR
(400 MHz, CDCl3)
2.02
2.13
2.16
0.97
1.00
2.08
3.17
Chemical Shift (ppm)

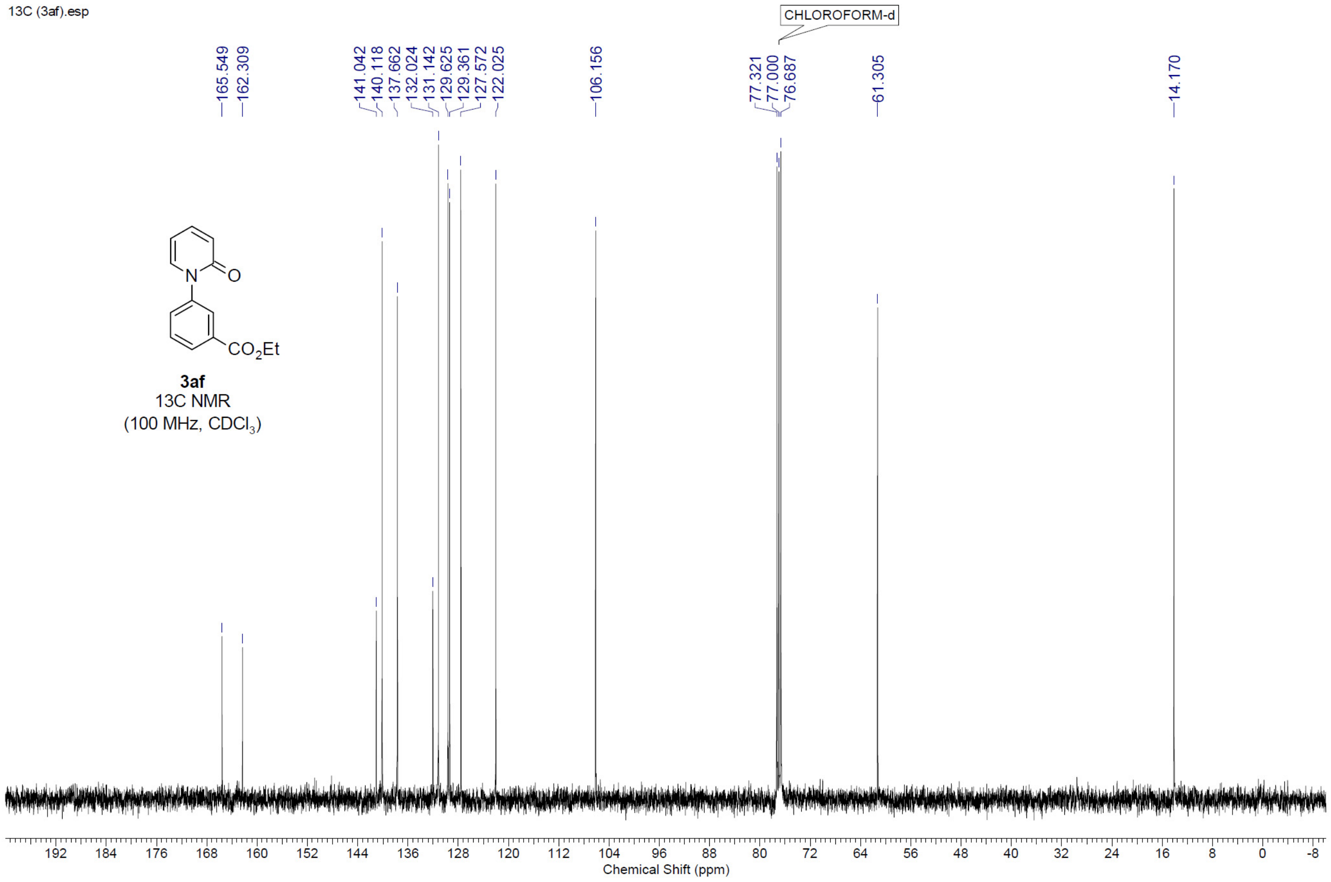
13C (3af).esp
CHLOROFORM-d
165.549
162.309
141.042
140.118
137.662
132.024
131.142
129.625
129.361
127.572
122.025
106.156
77.321
77.000
76.687
61.305
14.170
N
O
CO2Et
3af
13C NMR
(100 MHz, CDCl3)
Chemical Shift (ppm)

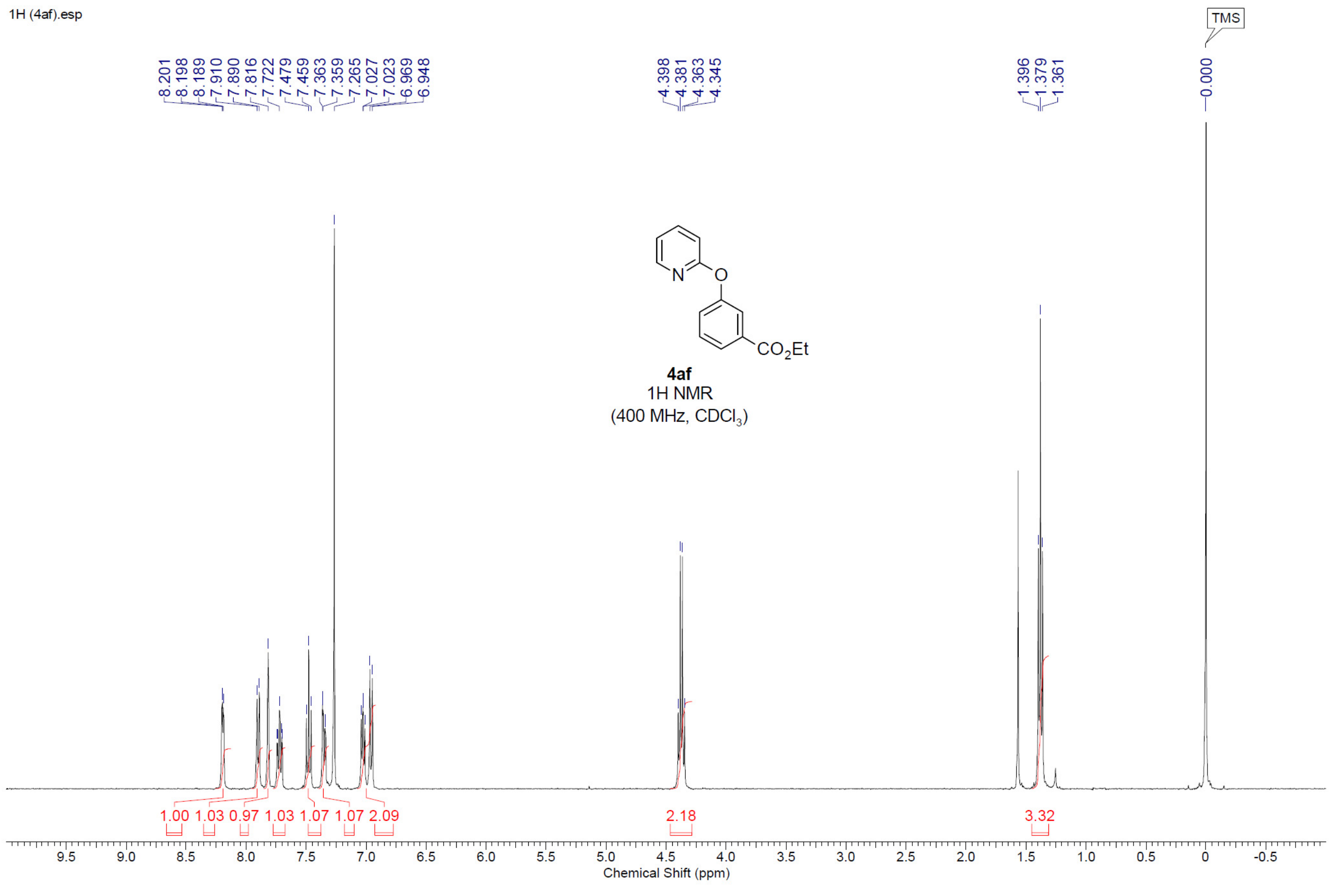
1H (4af).esp
TMS
8.201
8.198
8.189
7.910
7.890
7.816
7.722
7.479
7.459
7.363
7.359
7.265
7.027
7.023
6.969
6.948
4.398
4.381
4.363
4.345
1.396
1.379
1.361
0.000
N
O
CO_2Et
4af
1H NMR
(400 MHz, $CDCl_3$)
1.00 1.03 0.97 1.03 1.07 1.07 2.09
2.18
3.32
9.5 9.0 8.5 8.0 7.5 7.0 6.5 6.0 5.5 5.0 4.5 4.0 3.5 3.0 2.5 2.0 1.5 1.0 0.5 0 -0.5
Chemical Shift (ppm)

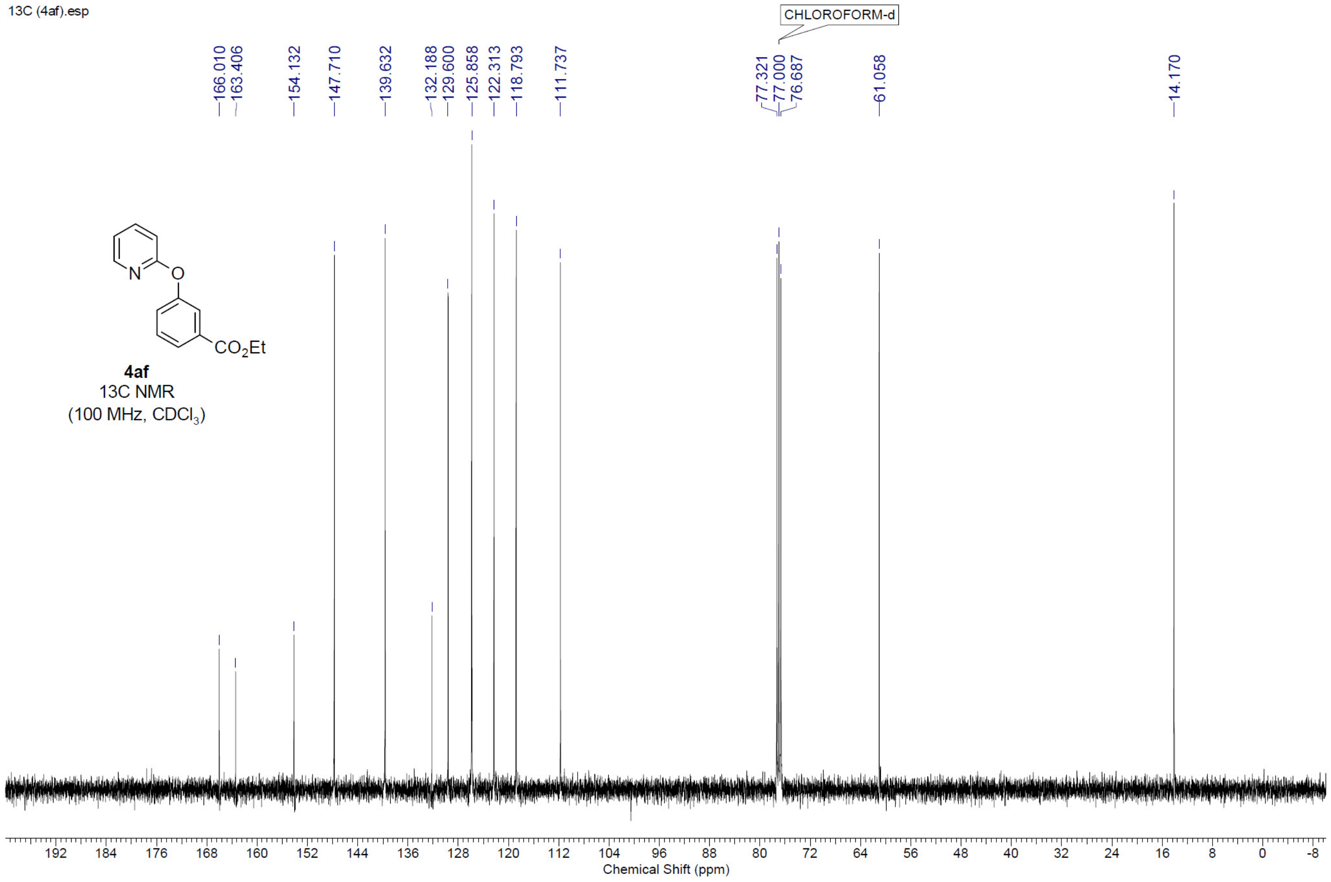
13C (4af).esp
CHLOROFORM-d
166.010
163.406
154.132
147.710
139.632
132.188
129.600
125.858
122.313
118.793
111.737
77.321
77.000
76.687
61.058
14.170
N
O
CO_2Et
4af
13C NMR
(100 MHz, $CDCl_3$)
192 184 176 168 160 152 144 136 128 120 112 104 96 88 80 72 64 56 48 40 32 24 16 8 0 -8
Chemical Shift (ppm)

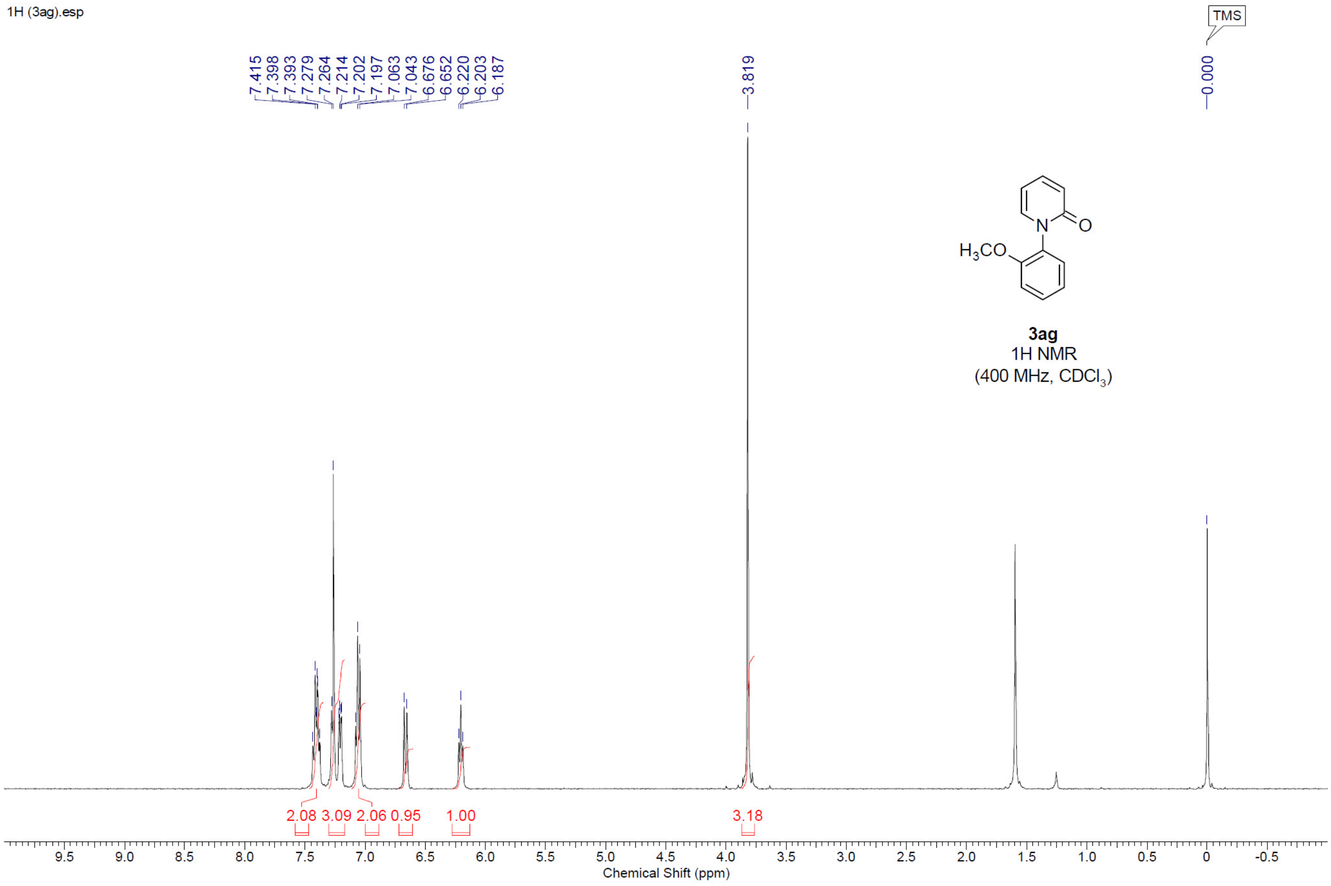
1H (3ag).esp
TMS
7.415
7.398
7.393
7.279
7.264
7.214
7.202
7.197
7.063
7.043
6.676
6.652
6.220
6.203
6.187
3.819
0.000
H3CO
N
O
3ag
1H NMR
(400 MHz, CDCl3)
2.08
3.09
2.06
0.95
1.00
3.18
Chemical Shift (ppm)

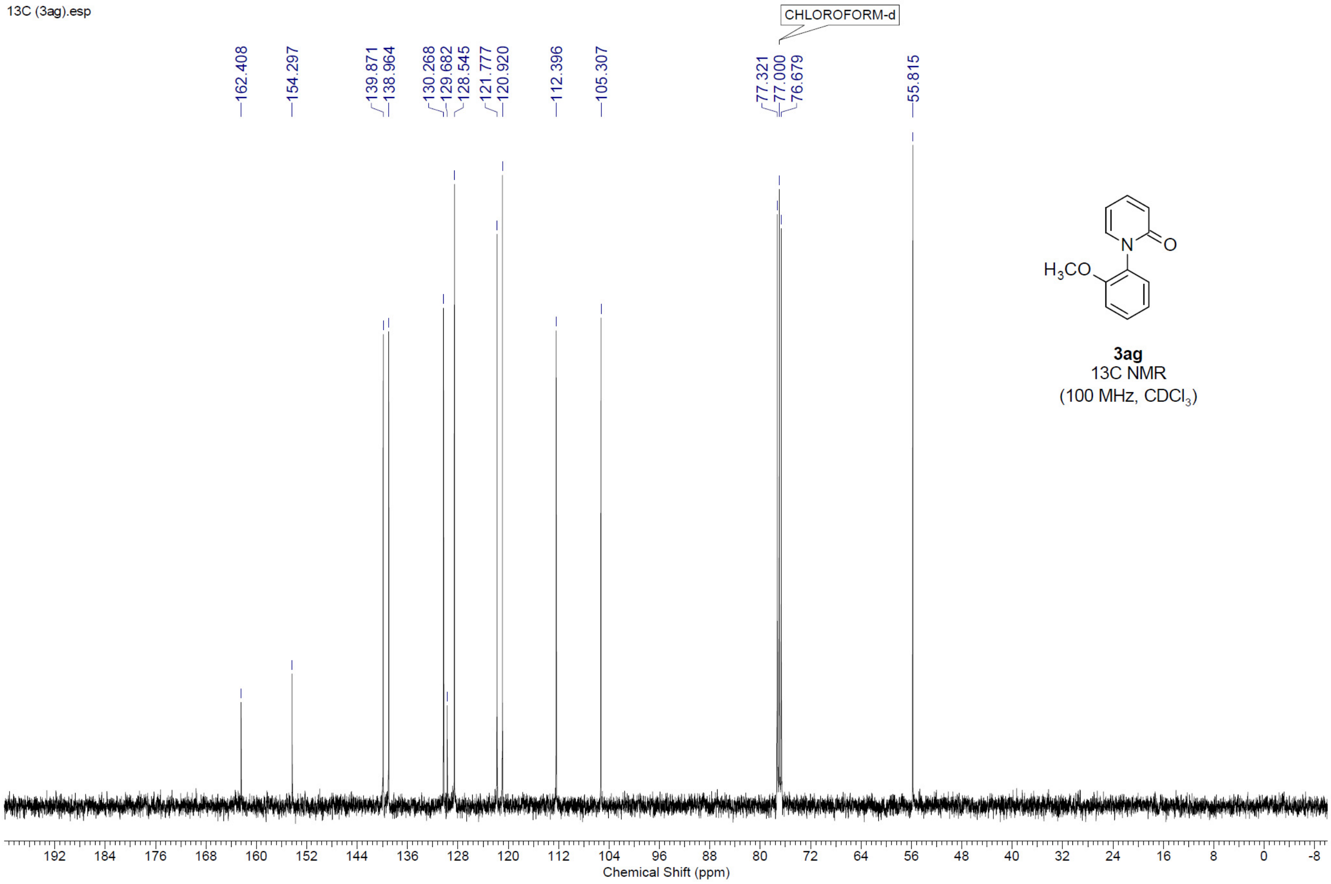
13C (3ag).esp
CHLOROFORM-d
162.408
154.297
139.871
138.964
130.268
129.682
128.545
121.777
120.920
112.396
105.307
77.321
77.000
76.679
55.815
H3CO
N
O
3ag
13C NMR
(100 MHz, CDCl3)
Chemical Shift (ppm)

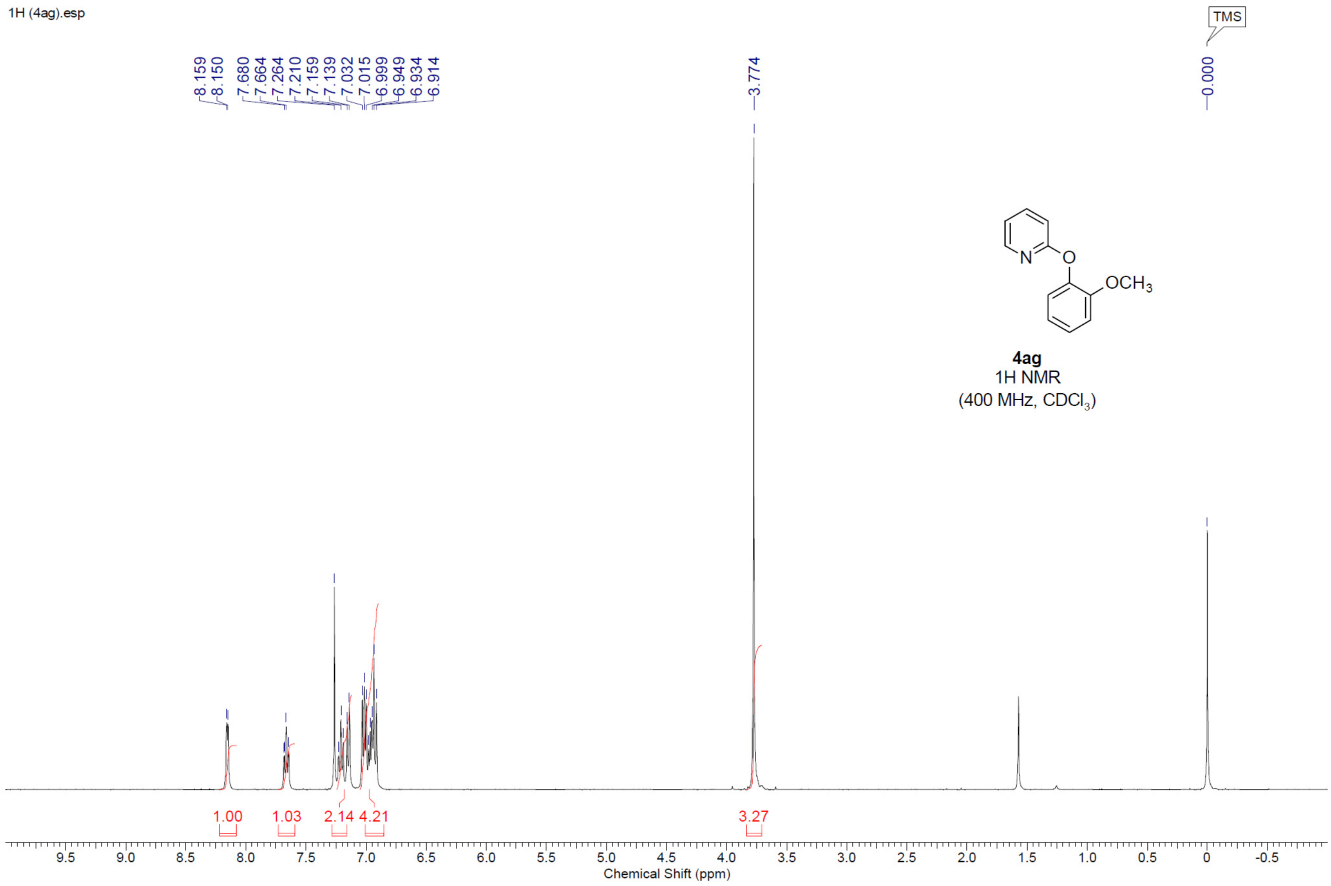
1H (4ag).esp
8.159
8.150
7.680
7.664
7.264
7.210
7.159
7.139
7.032
7.015
6.999
6.949
6.934
6.914
3.774
TMS
0.000
N
O
OCH3
4ag
1H NMR
(400 MHz, CDCl3)
1.00
1.03
2.14
4.21
3.27
9.5
9.0
8.5
8.0
7.5
7.0
6.5
6.0
5.5
5.0
4.5
4.0
3.5
3.0
2.5
2.0
1.5
1.0
0.5
0
-0.5
Chemical Shift (ppm)

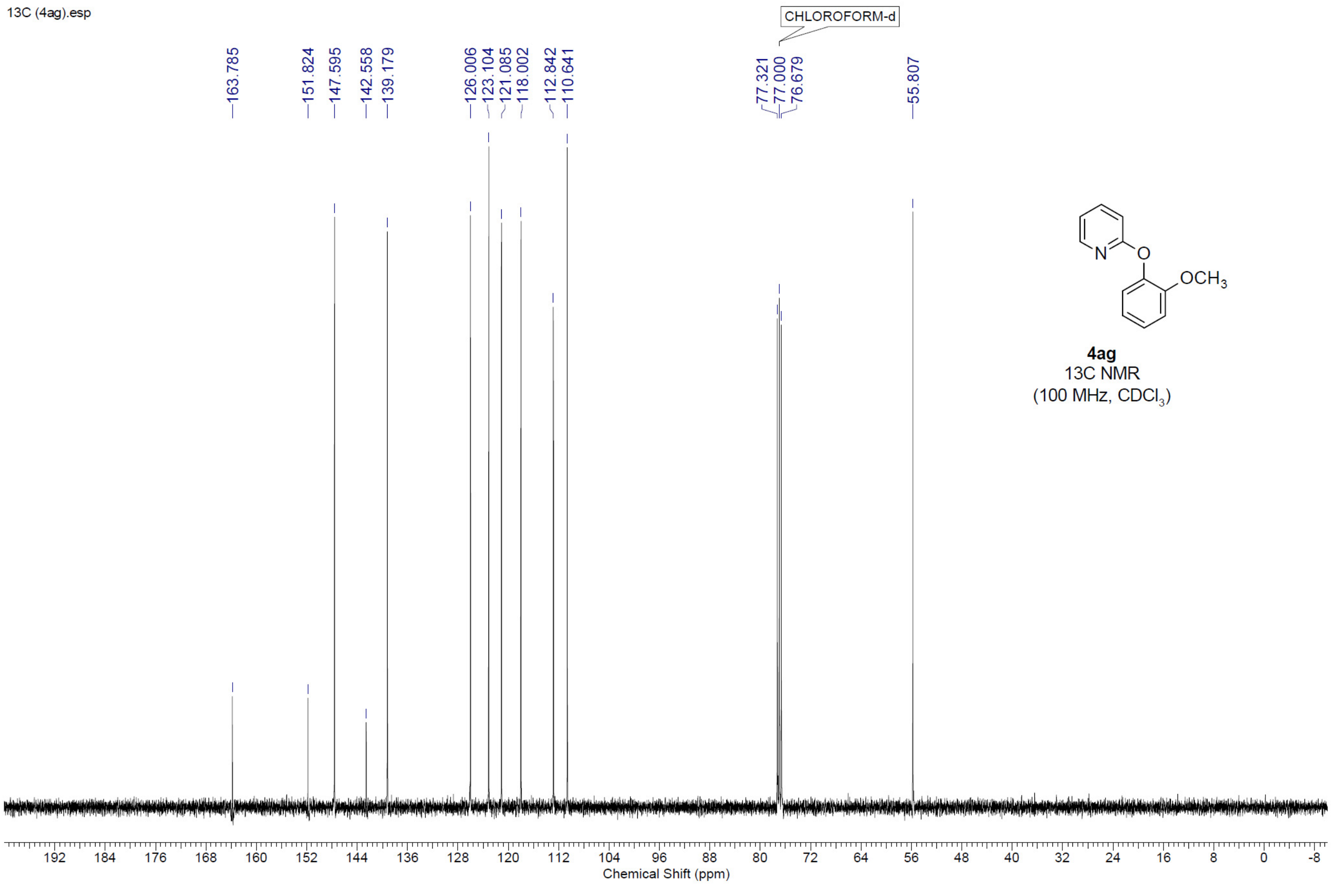
13C (4ag).esp
163.785
151.824
147.595
142.558
139.179
126.006
123.104
121.085
118.002
112.842
110.641
CHLOROFORM-d
77.321
77.000
76.679
55.807
N
O
OCH3
4ag
13C NMR
(100 MHz, CDCl3)
192
184
176
168
160
152
144
136
128
120
112
104
96
88
80
72
64
56
48
40
32
24
16
8
0
-8
Chemical Shift (ppm)

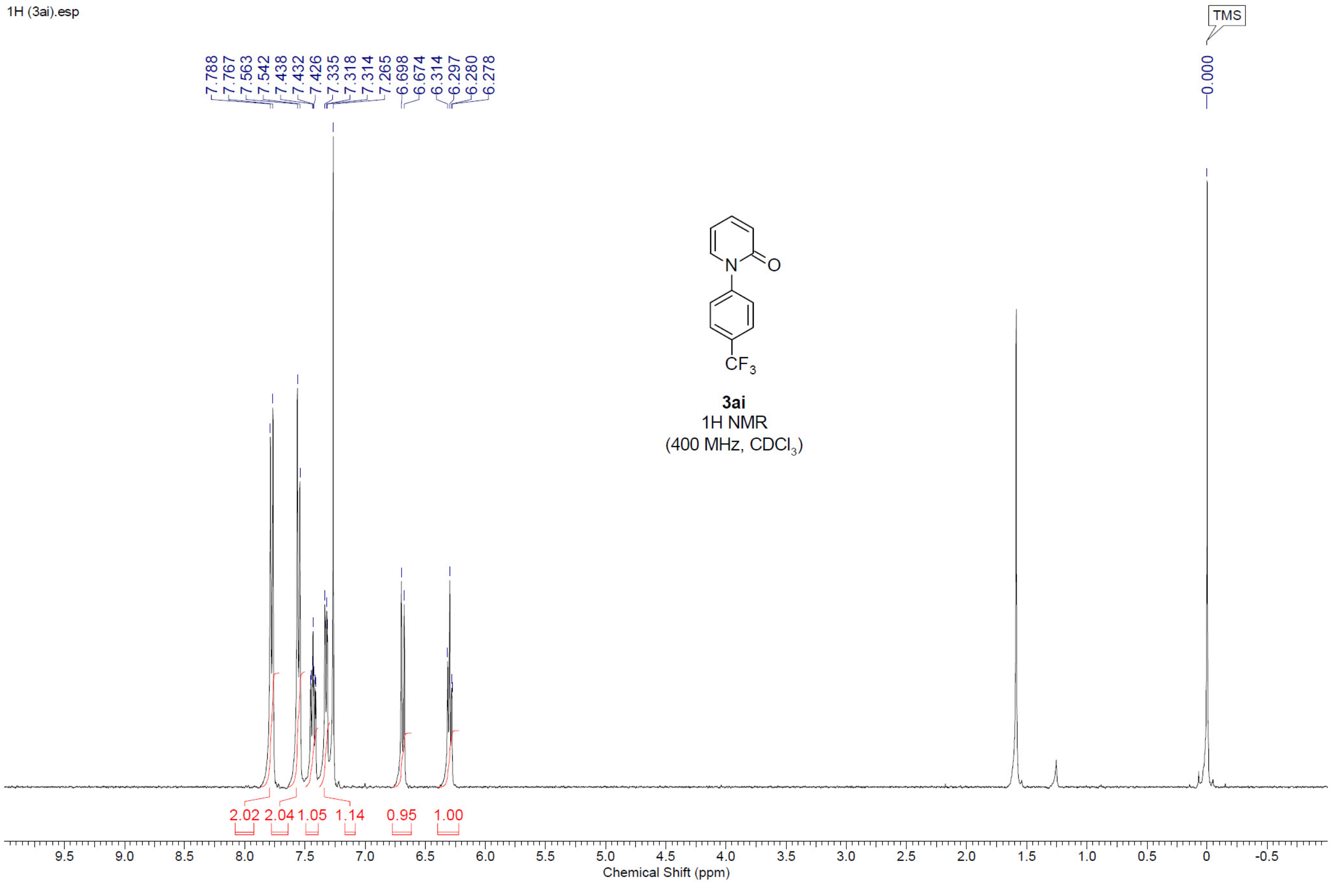

1H (3ai).esp
7.788
7.767
7.563
7.542
7.438
7.432
7.426
7.335
7.318
7.314
7.265
6.698
6.674
6.314
6.297
6.280
6.278
TMS
0.000
N
O
CF3
3ai
1H NMR
(400 MHz, CDCl3)
2.02
2.04
1.05
1.14
0.95
1.00
Chemical Shift (ppm)

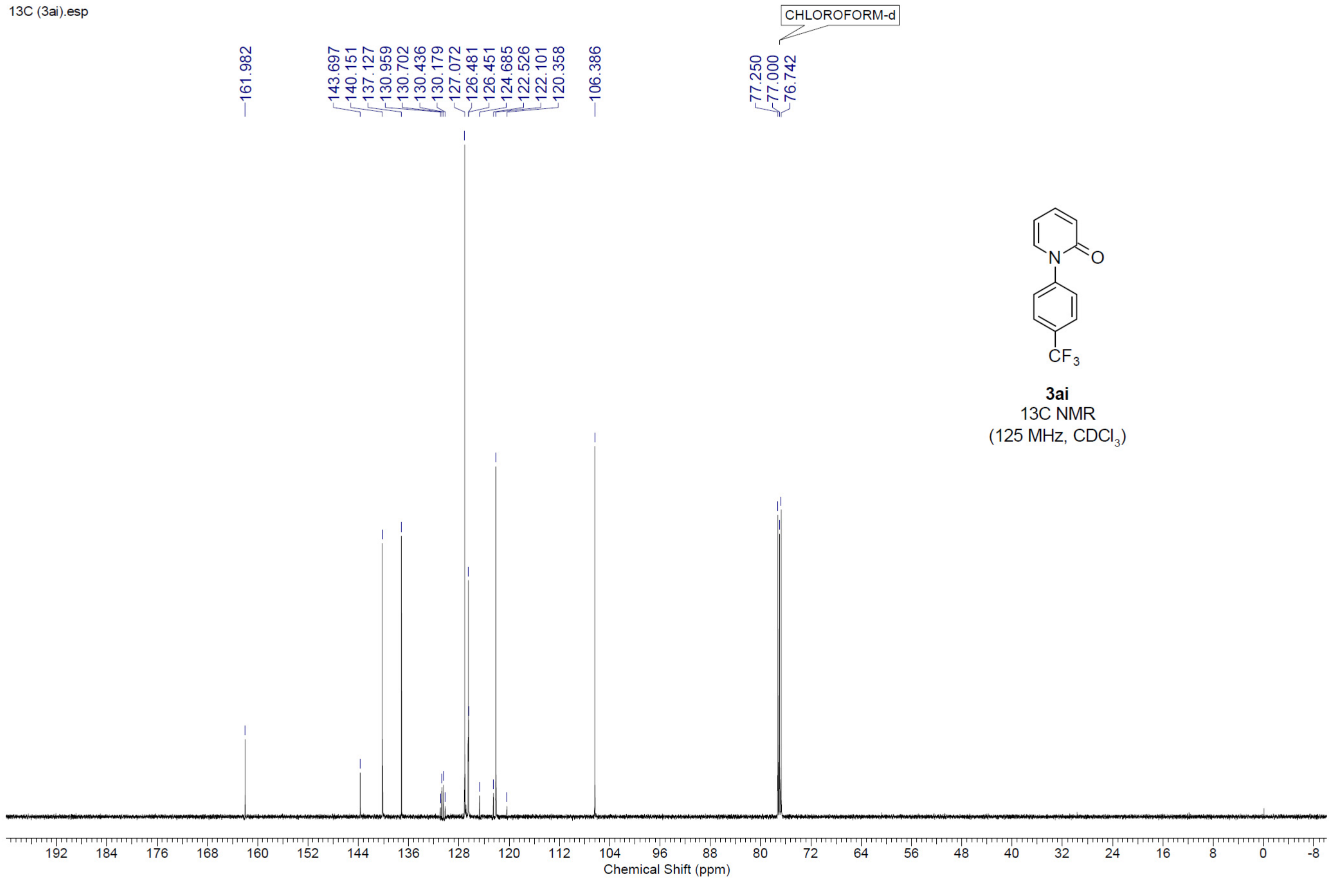

13C (3ai).esp
161.982
143.697
140.151
137.127
130.959
130.702
130.436
130.179
127.072
126.481
126.451
124.685
122.526
122.101
120.358
106.386
CHLOROFORM-d
77.250
77.000
76.742
N
O
CF3
3ai
13C NMR
(125 MHz, CDCl3)
Chemical Shift (ppm)

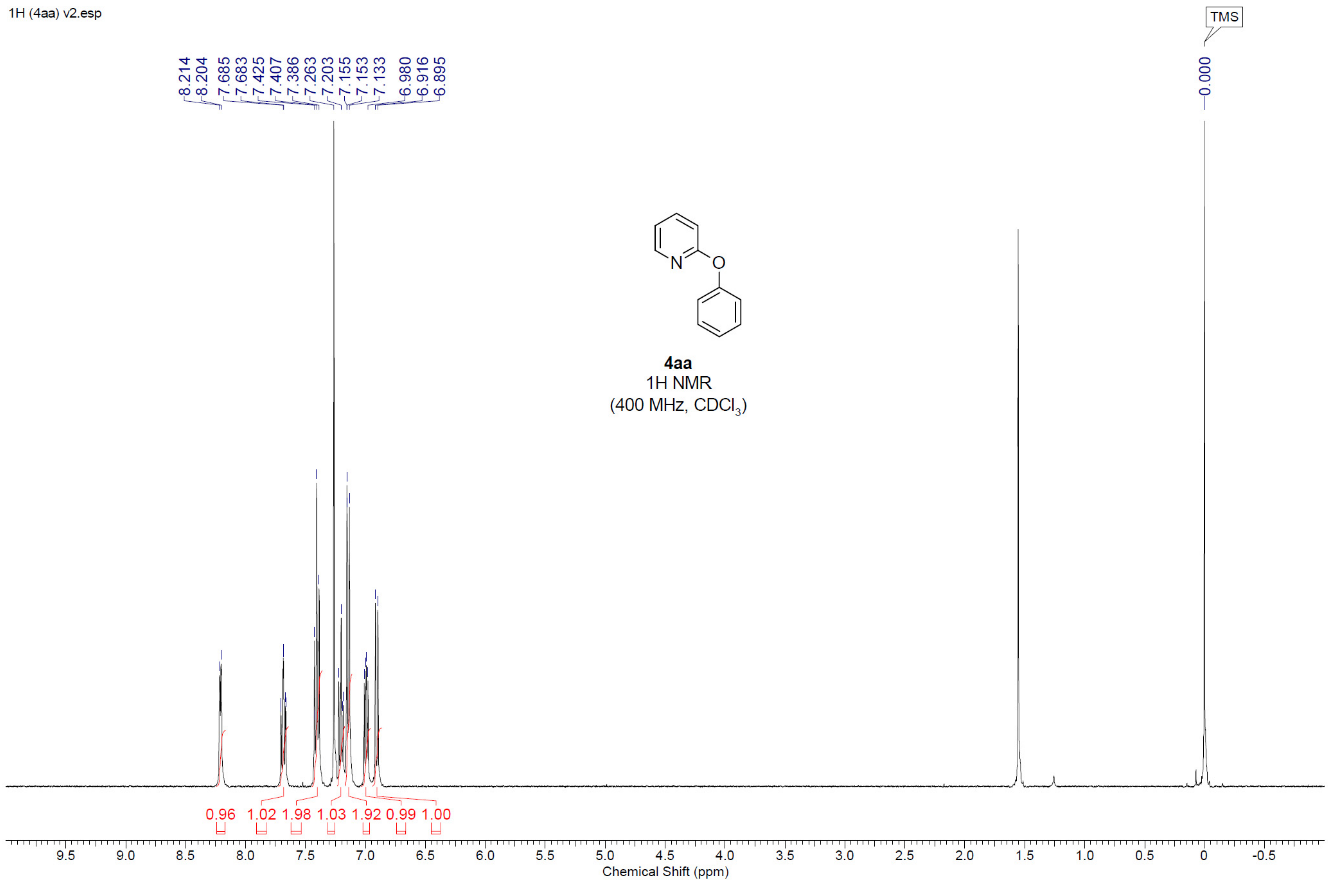
1H (4aa) v2.esp
8.214
8.204
7.685
7.683
7.425
7.407
7.386
7.263
7.203
7.155
7.153
7.133
6.980
6.916
6.895
TMS
0.000
N
O
4aa
1H NMR
(400 MHz, CDCl3)
0.96
1.02
1.98
1.03
1.92
0.99
1.00
9.5
9.0
8.5
8.0
7.5
7.0
6.5
6.0
5.5
5.0
4.5
4.0
3.5
3.0
2.5
2.0
1.5
1.0
0.5
0
-0.5
Chemical Shift (ppm)

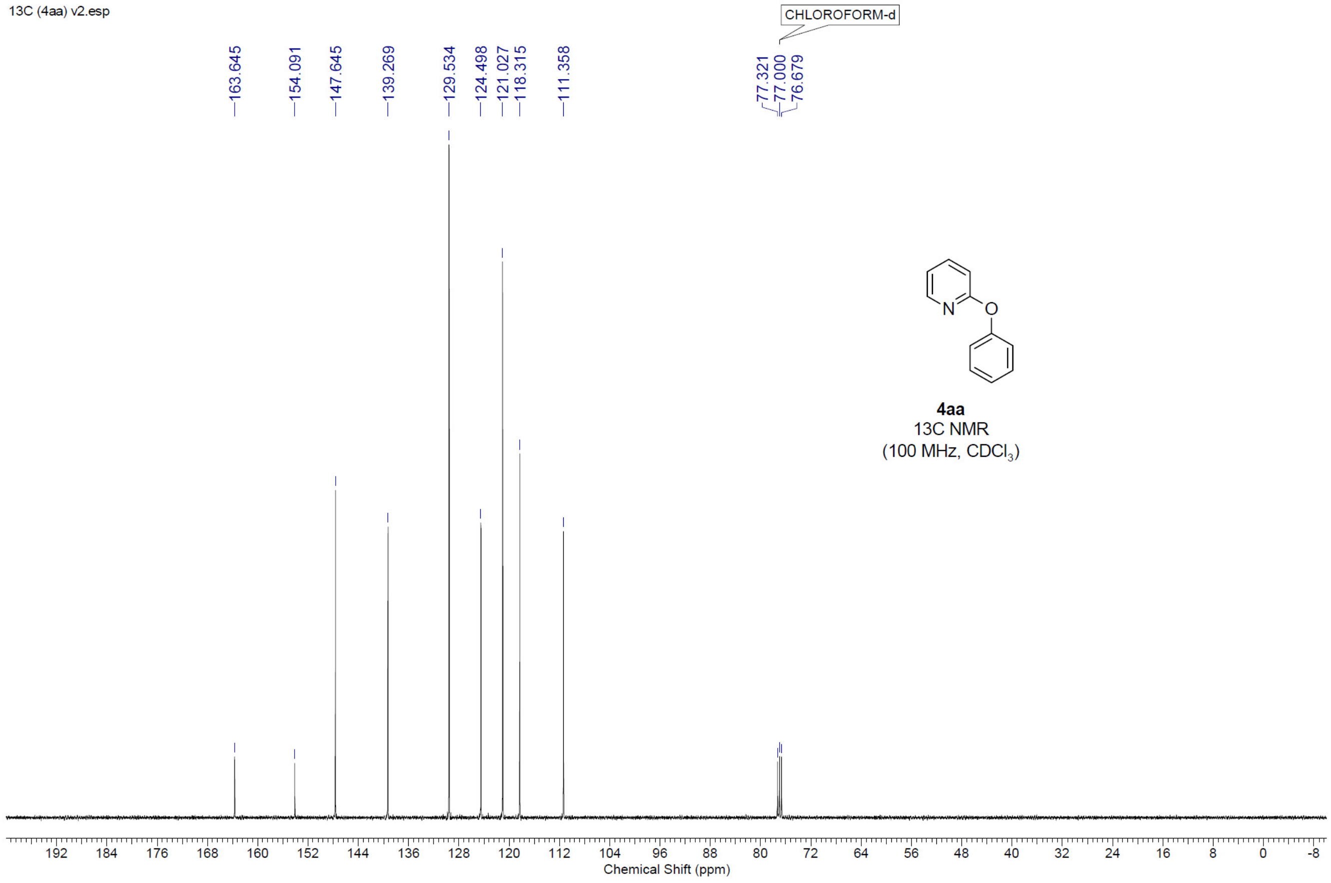
13C (4aa) v2.esp
163.645
154.091
147.645
139.269
129.534
124.498
121.027
118.315
111.358
CHLOROFORM-d
77.321
77.000
76.679
N
O
4aa
13C NMR
(100 MHz, CDCl3)
192
184
176
168
160
152
144
136
128
120
112
104
96
88
80
72
64
56
48
40
32
24
16
8
0
-8
Chemical Shift (ppm)

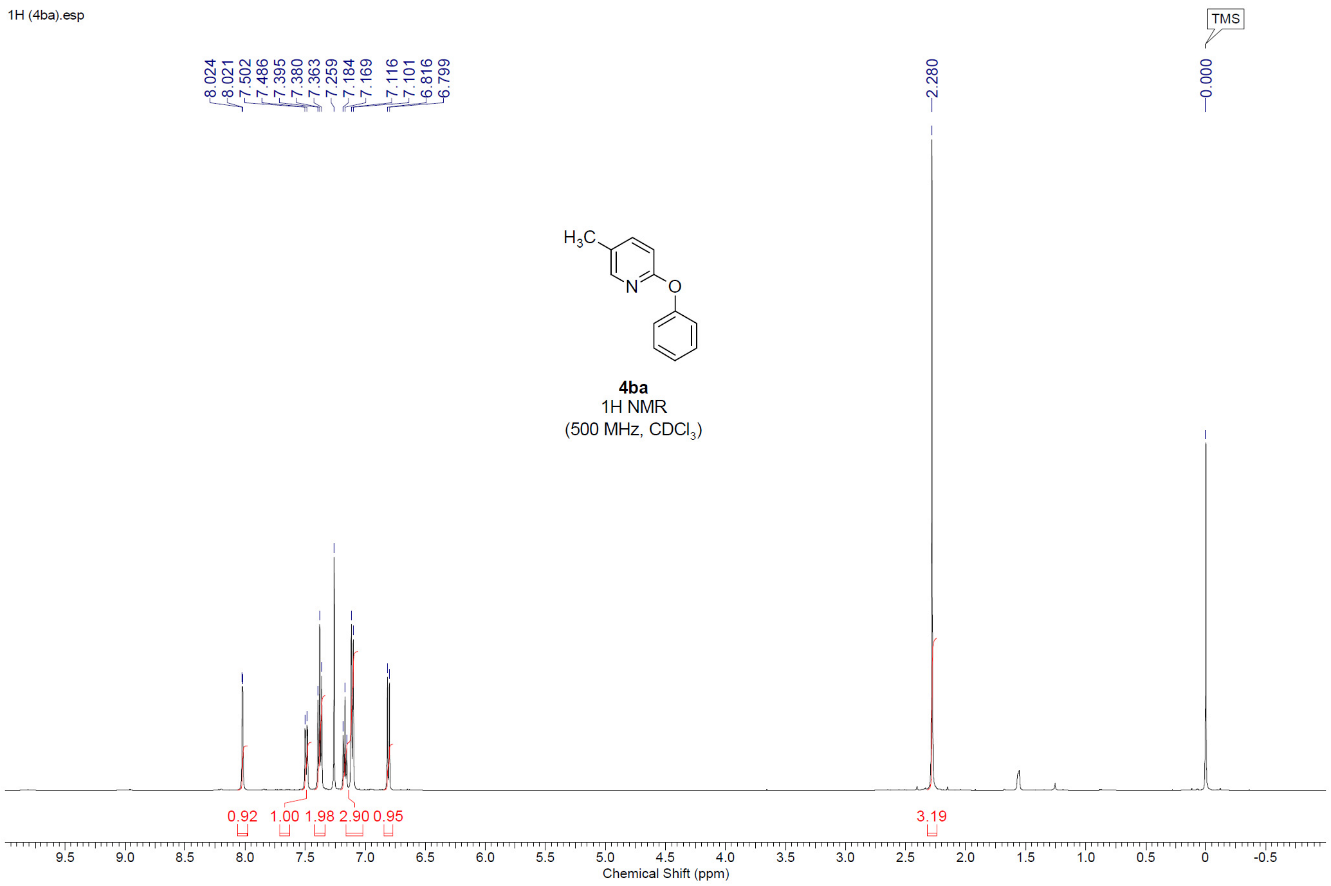
1H (4ba).esp
TMS
8.024
8.021
7.502
7.486
7.395
7.380
7.363
7.259
7.184
7.169
7.116
7.101
6.816
6.799
2.280
0.000
H_3C
N
O
4ba
1H NMR
(500 MHz, $CDCl_3$)
0.92
1.00
1.98
2.90
0.95
3.19
Chemical Shift (ppm)

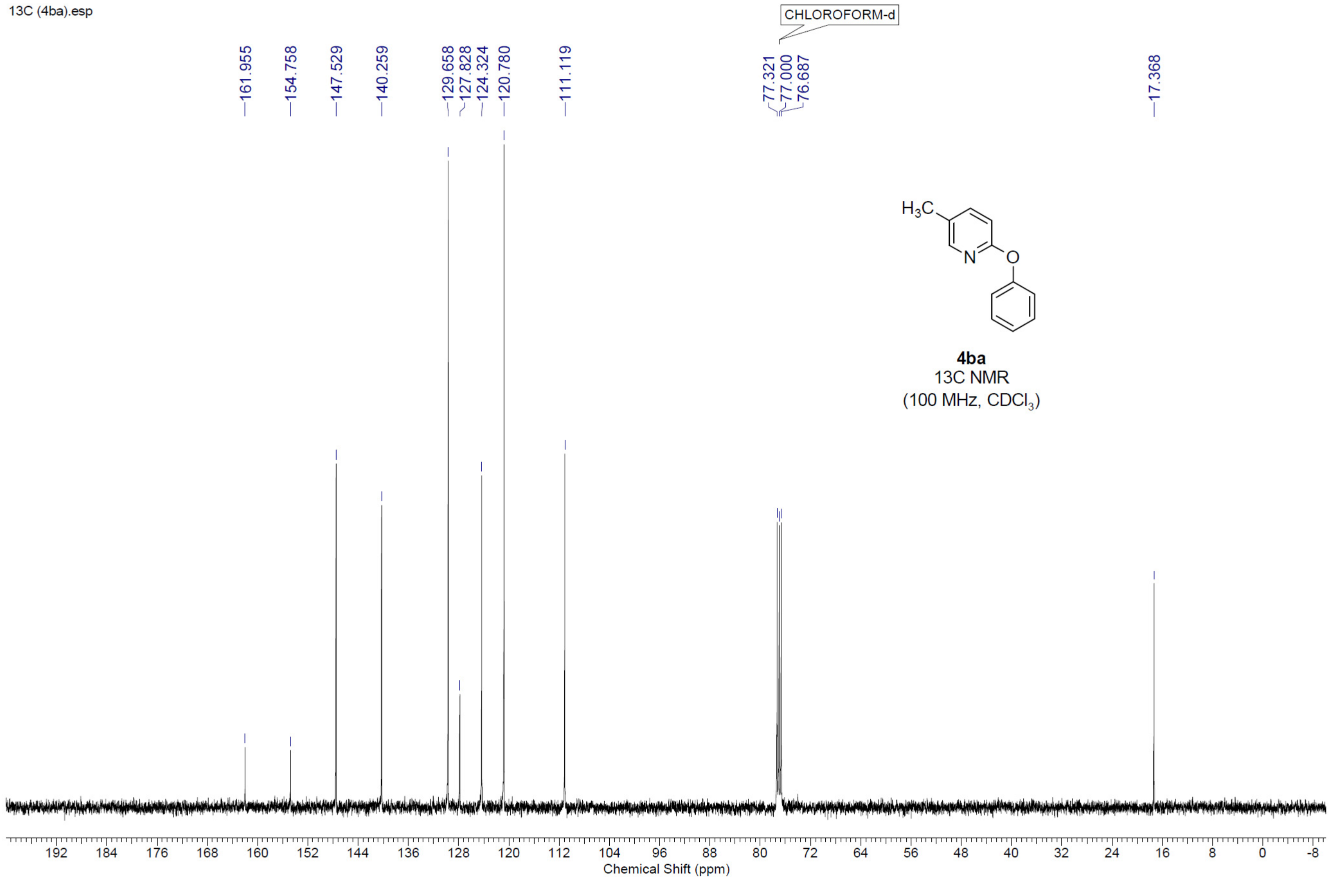
13C (4ba).esp
CHLOROFORM-d
161.955
154.758
147.529
140.259
129.658
127.828
124.324
120.780
111.119
77.321
77.000
76.687
17.368
H_3C
N
O
4ba
13C NMR
(100 MHz, $CDCl_3$)
Chemical Shift (ppm)

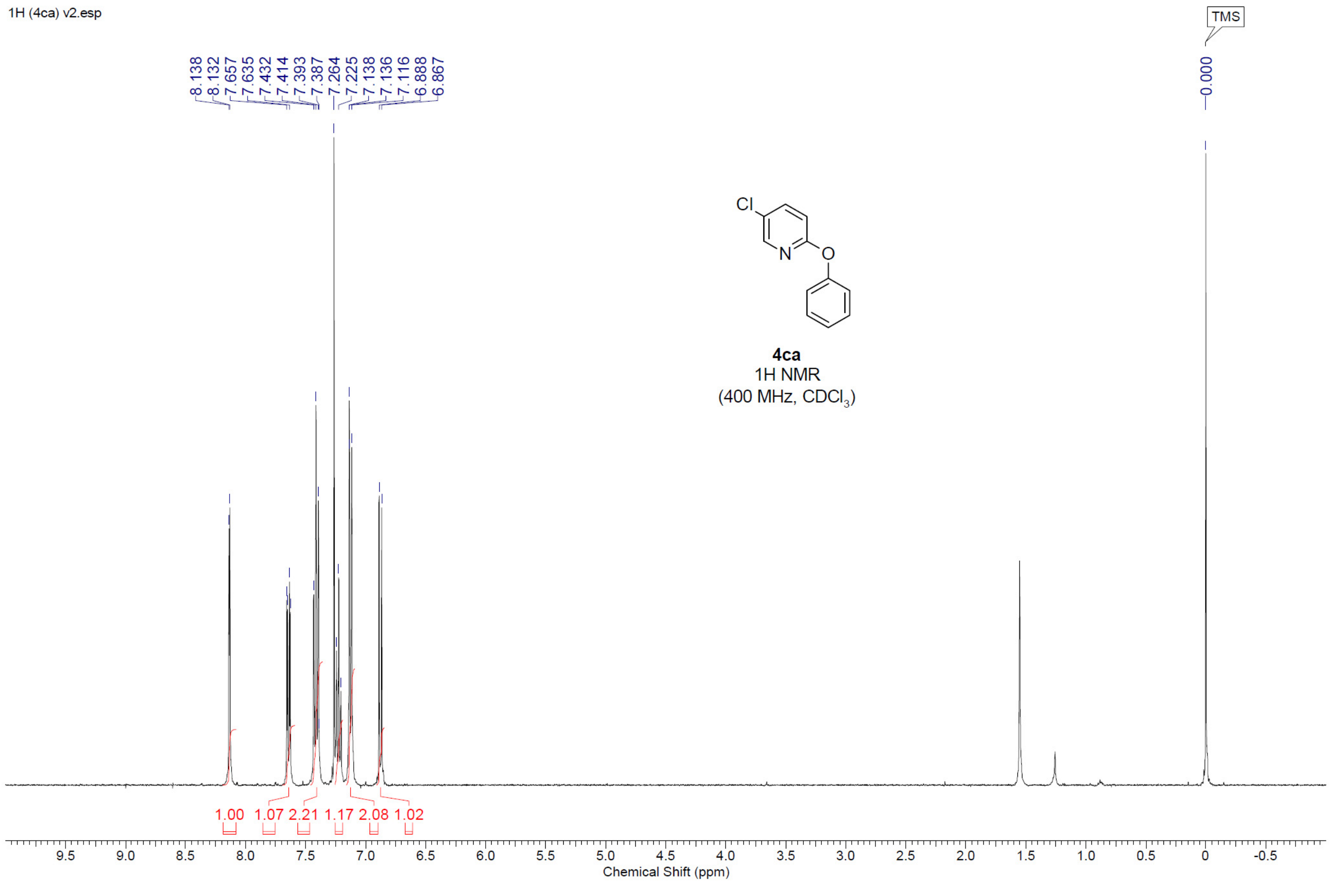
1H (4ca) v2.esp
TMS
8.138
8.132
7.657
7.635
7.432
7.414
7.393
7.387
7.264
7.225
7.138
7.136
7.116
6.888
6.867
-0.000
Cl
N
O
4ca
1H NMR
(400 MHz, CDCl3)
1.00 1.07 2.21 1.17 2.08 1.02
Chemical Shift (ppm)

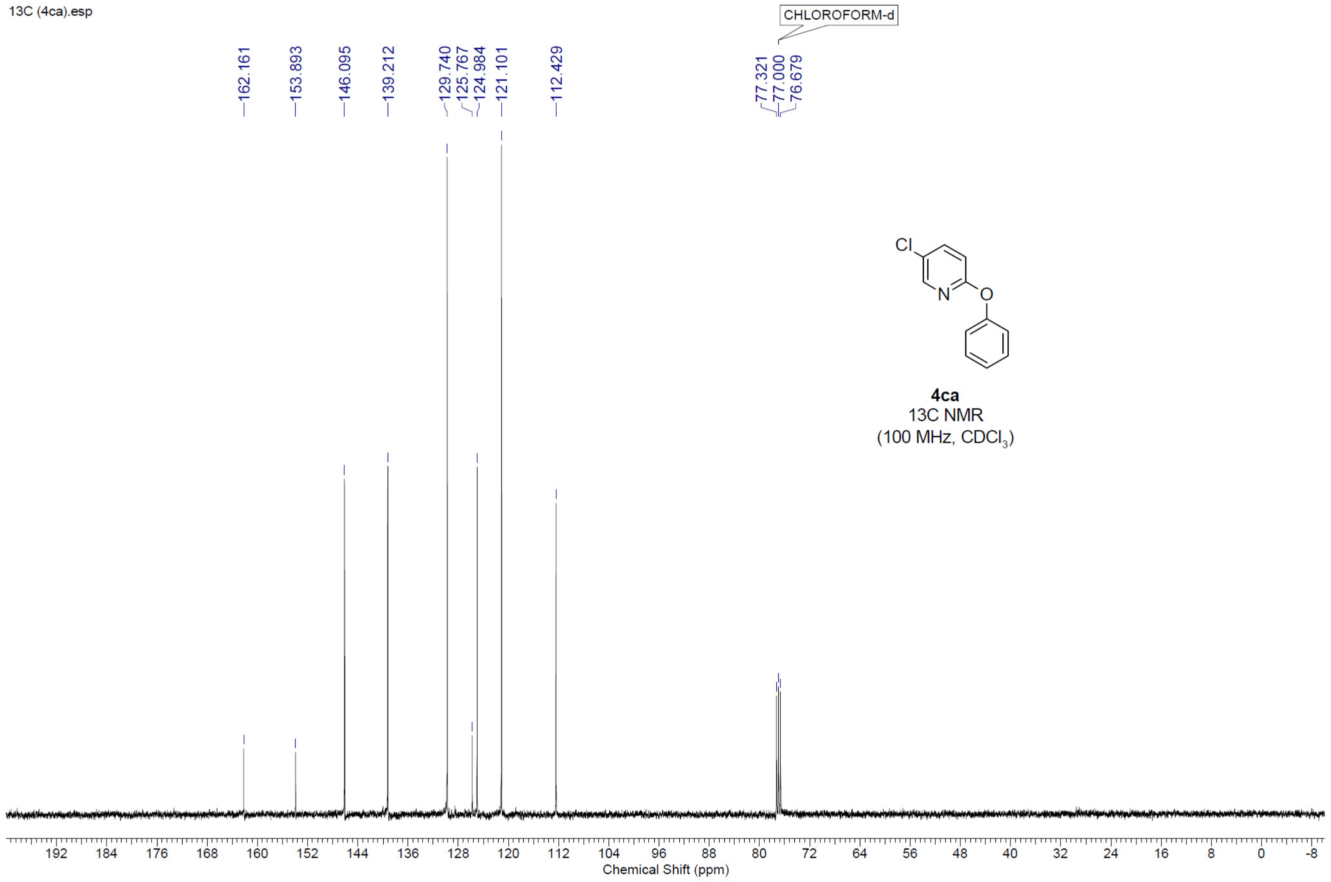
13C (4ca).esp
CHLOROFORM-d
162.161
153.893
146.095
139.212
129.740
125.767
124.984
121.101
112.429
77.321
77.000
76.679
Cl
N
O
4ca
13C NMR
(100 MHz, CDCl3)
Chemical Shift (ppm)

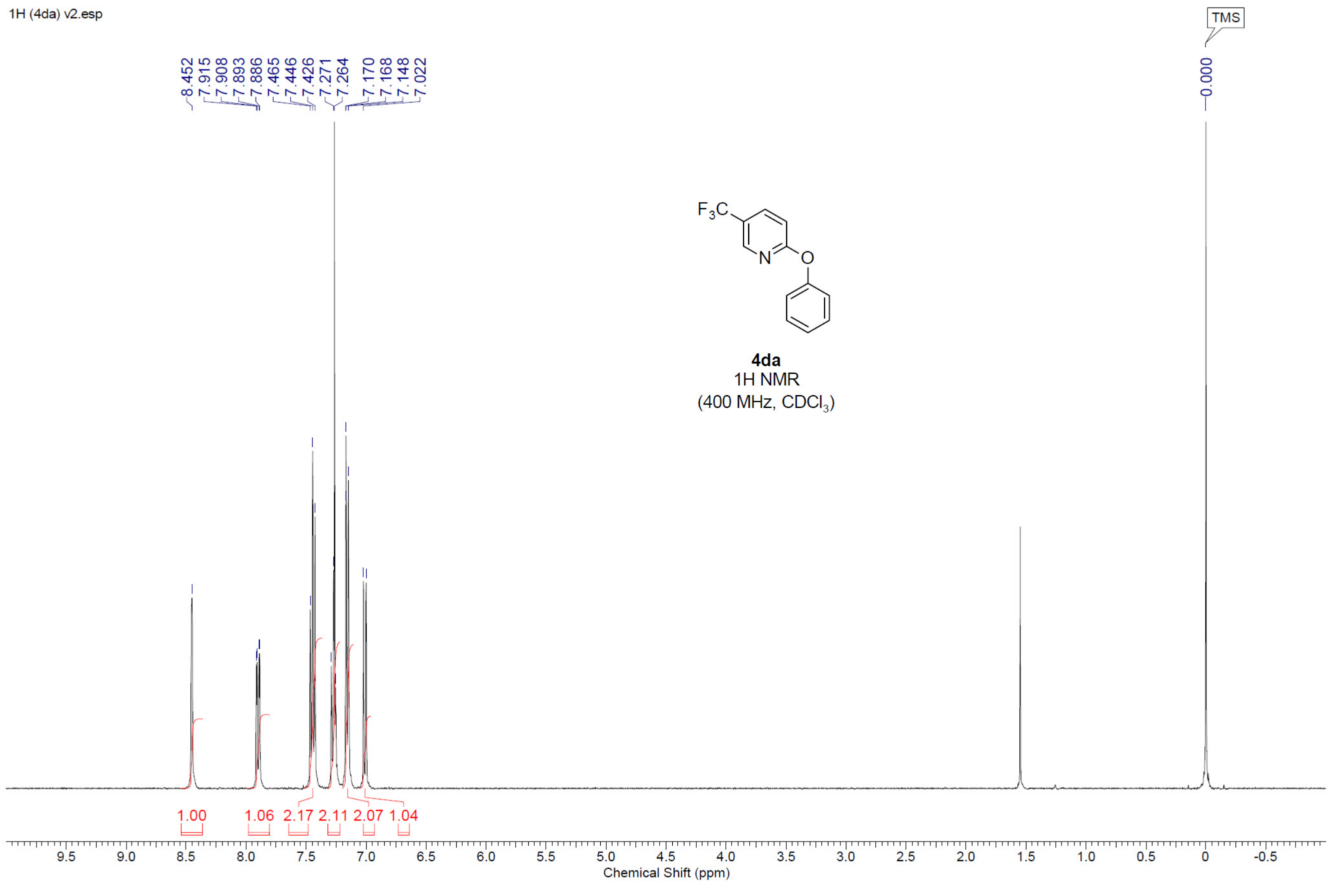
1H (4da) v2.esp
TMS
8.452
7.915
7.908
7.893
7.886
7.465
7.446
7.426
7.271
7.264
7.170
7.168
7.148
7.022
0.000
F3C
N
O
4da
1H NMR
(400 MHz, CDCl3)
1.00
1.06
2.17
2.11
2.07
1.04
Chemical Shift (ppm)

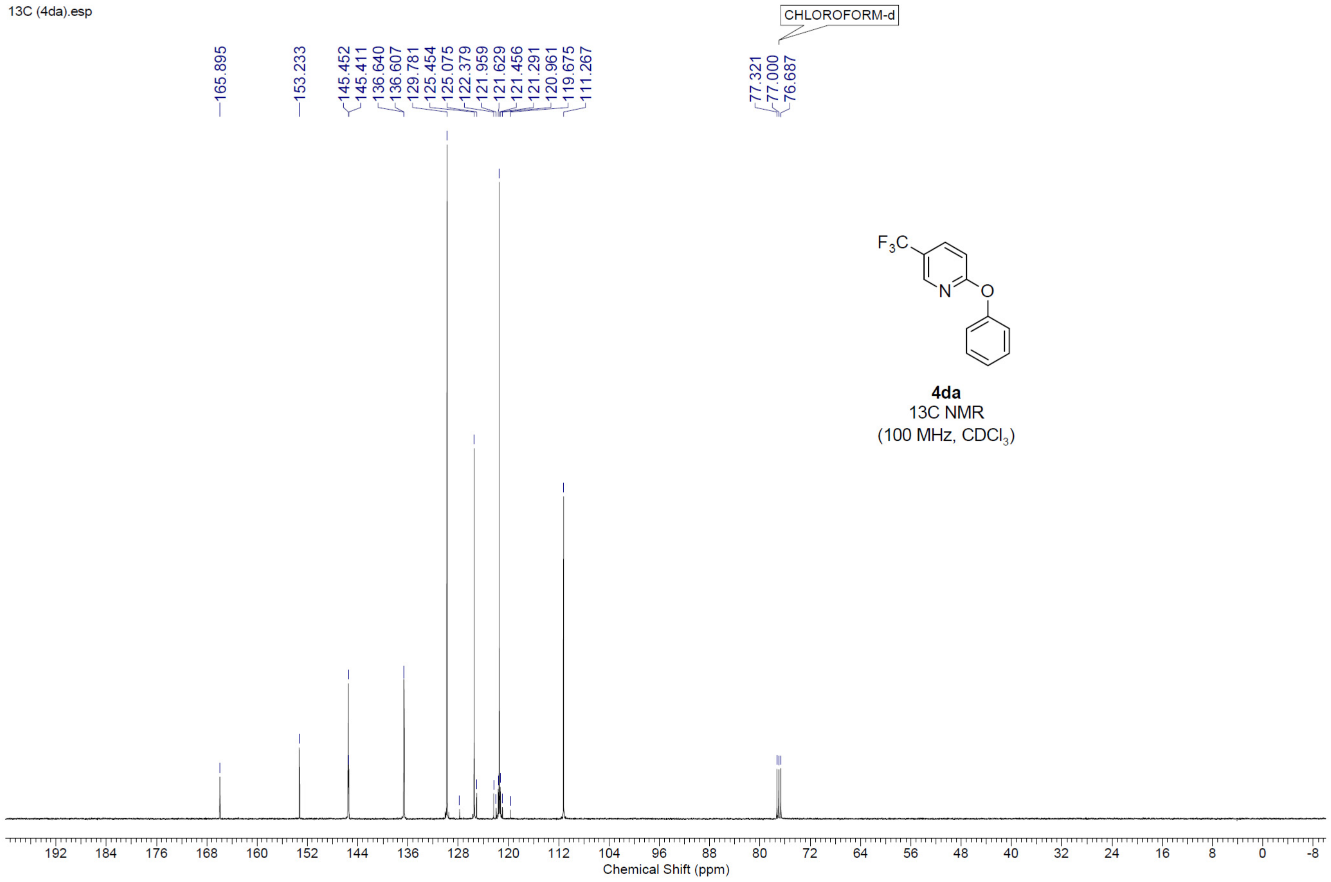
13C (4da).esp
CHLOROFORM-d
165.895
153.233
145.452
145.411
136.640
136.607
129.781
125.454
125.075
122.379
121.959
121.629
121.456
121.291
120.961
119.675
111.267
77.321
77.000
76.687
F3C
N
O
4da
13C NMR
(100 MHz, CDCl3)
Chemical Shift (ppm)

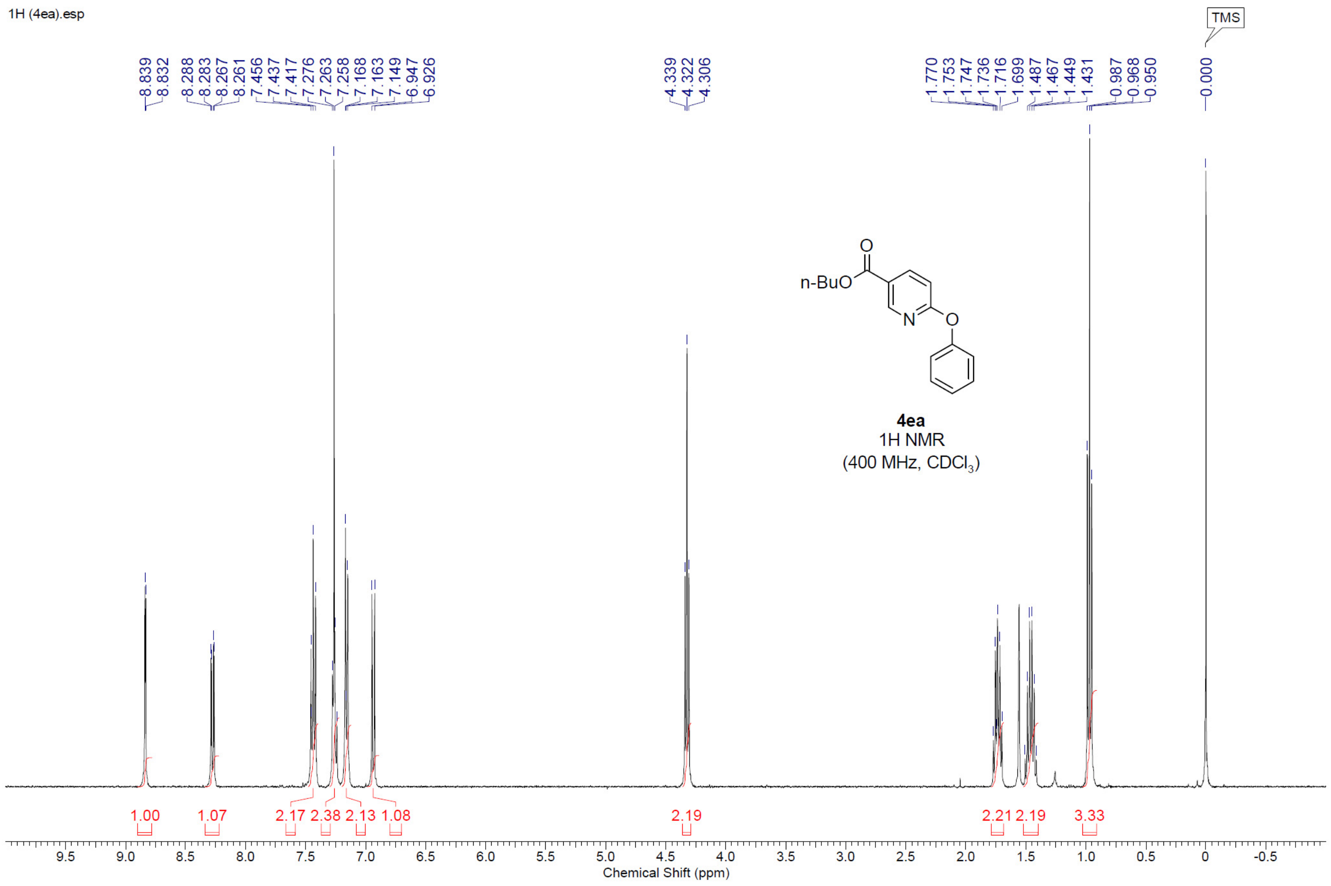
1H (4ea).esp
8.839
8.832
8.288
8.283
8.267
8.261
7.456
7.437
7.417
7.276
7.263
7.258
7.168
7.163
7.149
6.947
6.926
4.339
4.322
4.306
1.770
1.753
1.747
1.736
1.716
1.699
1.487
1.467
1.449
1.431
0.987
0.968
0.950
0.000
TMS
n-BuO
O
N
O
4ea
1H NMR
(400 MHz, CDCl3)
1.00
1.07
2.17
2.38
2.13
1.08
2.19
2.21
2.19
3.33
Chemical Shift (ppm)

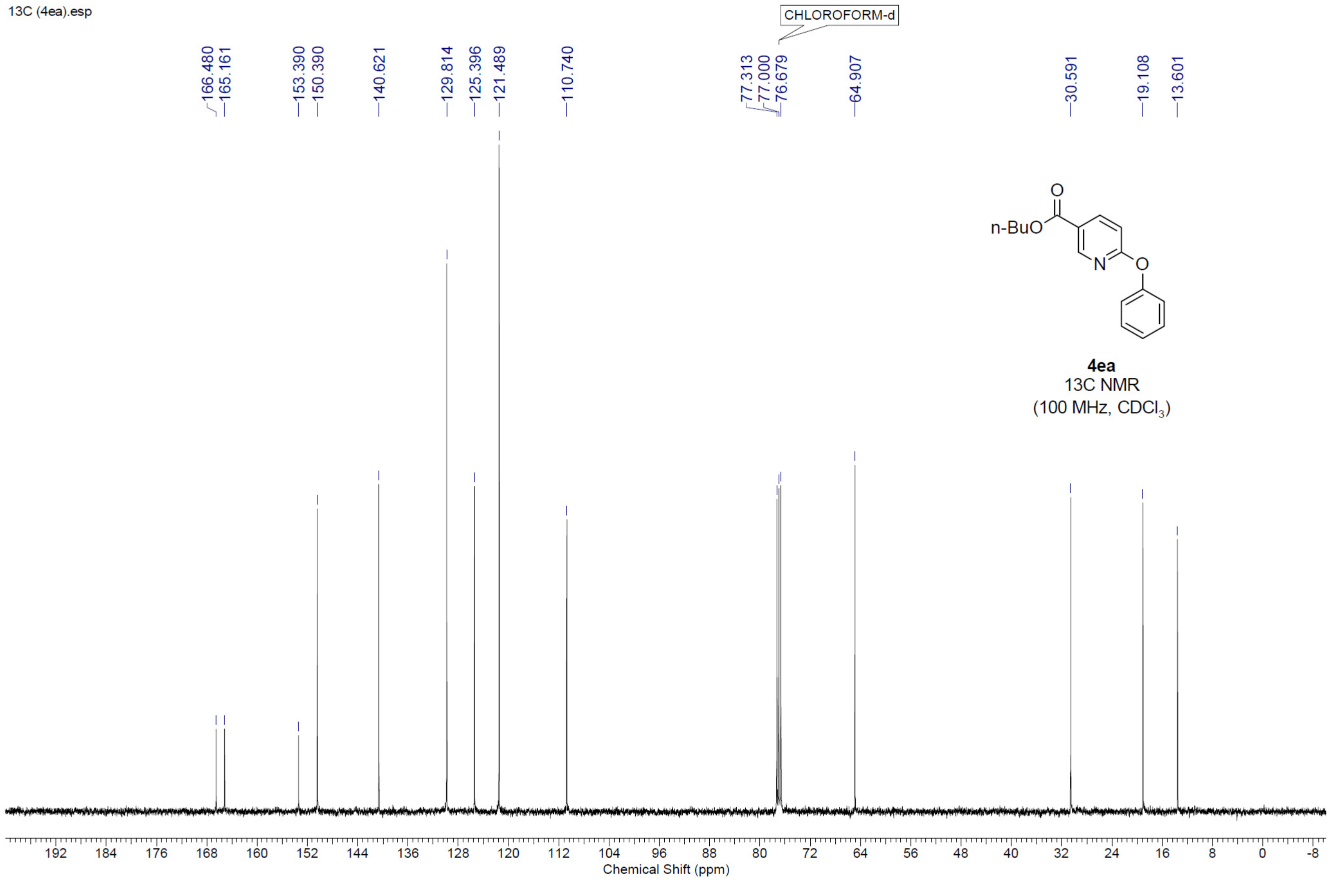
13C (4ea).esp
166.480
165.161
153.390
150.390
140.621
129.814
125.396
121.489
110.740
77.313
77.000
76.679
CHLOROFORM-d
64.907
30.591
19.108
13.601
n-BuO
O
N
O
4ea
13C NMR
(100 MHz, CDCl3)
Chemical Shift (ppm)

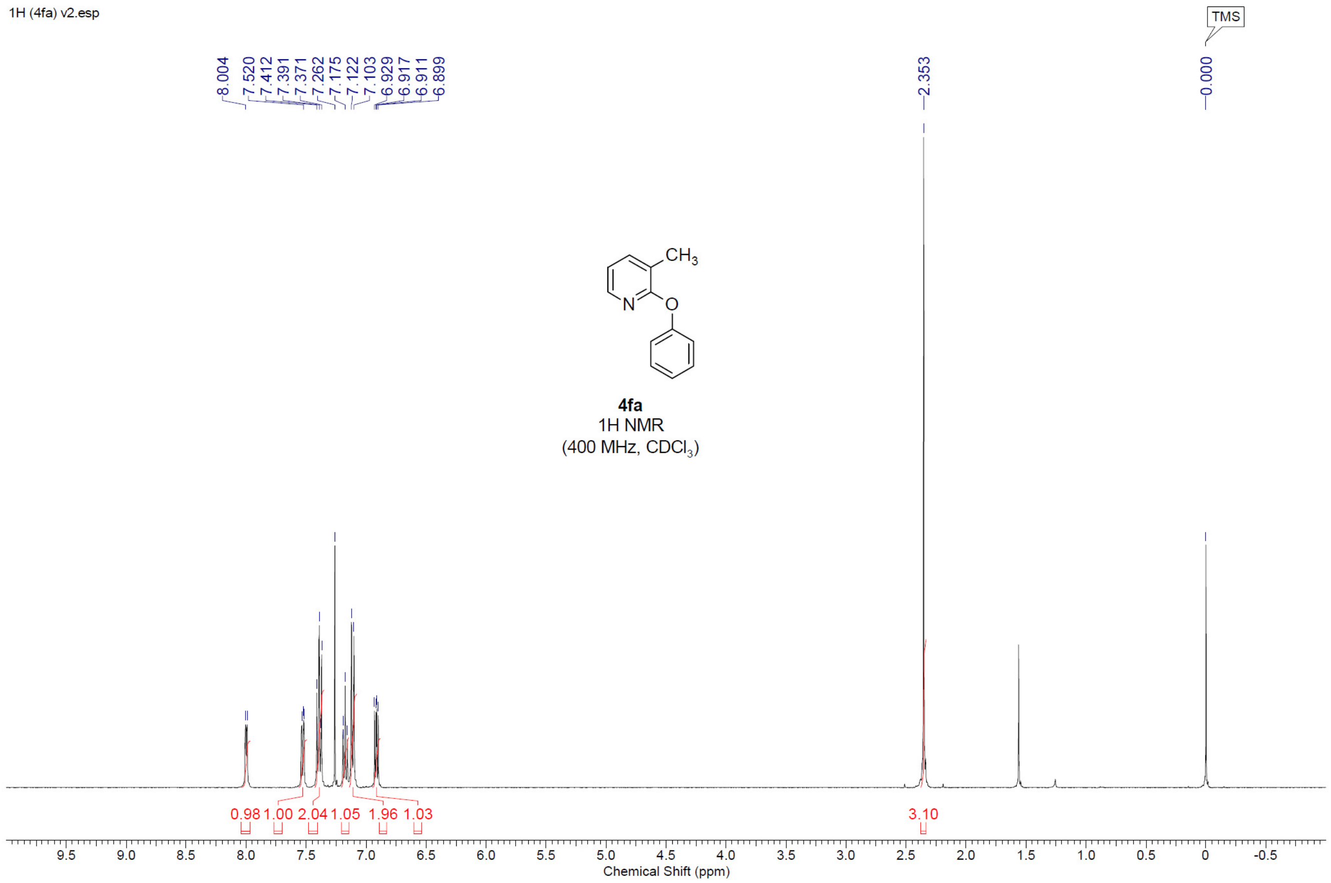
1H (4fa) v2.esp
TMS
8.004
7.520
7.412
7.391
7.371
7.262
7.175
7.122
7.103
6.929
6.917
6.911
6.899
2.353
0.000
CH3
N
O
4fa
1H NMR
(400 MHz, CDCl3)
0.98
1.00
2.04
1.05
1.96
1.03
3.10
Chemical Shift (ppm)

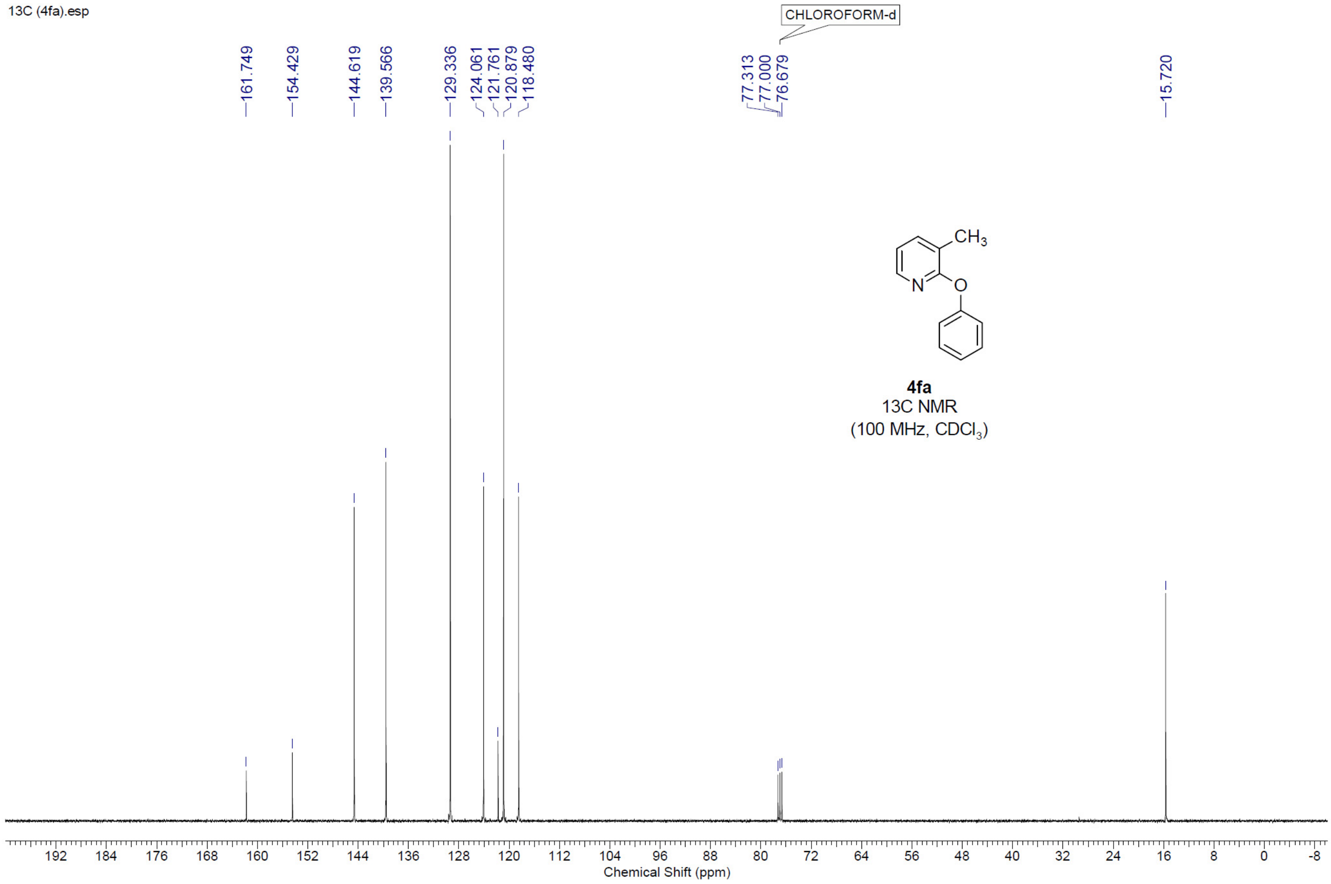
13C (4fa).esp
CHLOROFORM-d
161.749
154.429
144.619
139.566
129.336
124.061
121.761
120.879
118.480
77.313
77.000
76.679
15.720
CH3
N
O
4fa
13C NMR
(100 MHz, CDCl3)
Chemical Shift (ppm)

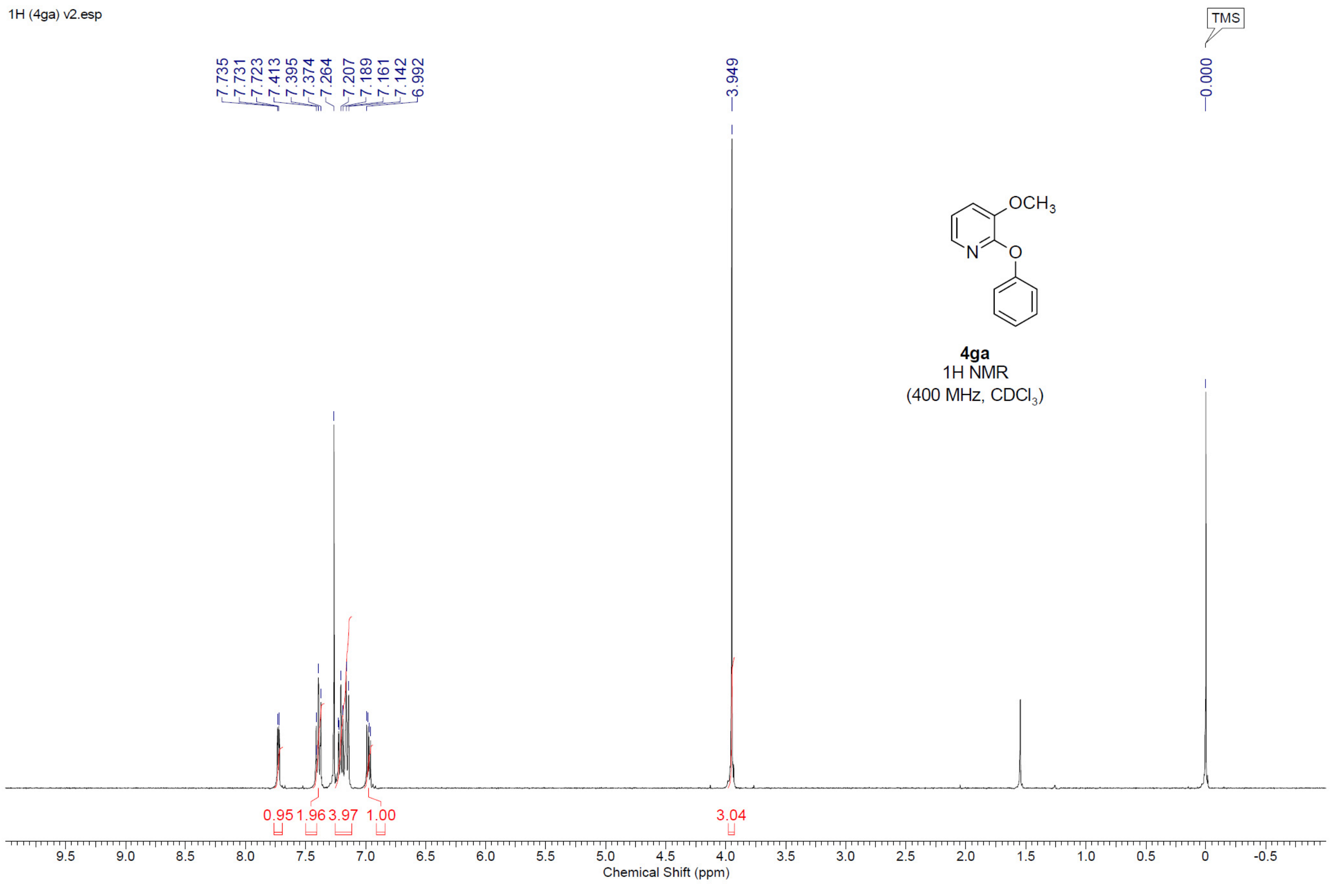
1H (4ga) v2.esp
TMS
7.735
7.731
7.723
7.413
7.395
7.374
7.264
7.207
7.189
7.161
7.142
6.992
3.949
0.000
OCH3
N
O
4ga
1H NMR
(400 MHz, CDCl3)
0.95
1.96
3.97
1.00
3.04
9.5
9.0
8.5
8.0
7.5
7.0
6.5
6.0
5.5
5.0
4.5
4.0
3.5
3.0
2.5
2.0
1.5
1.0
0.5
0
-0.5
Chemical Shift (ppm)

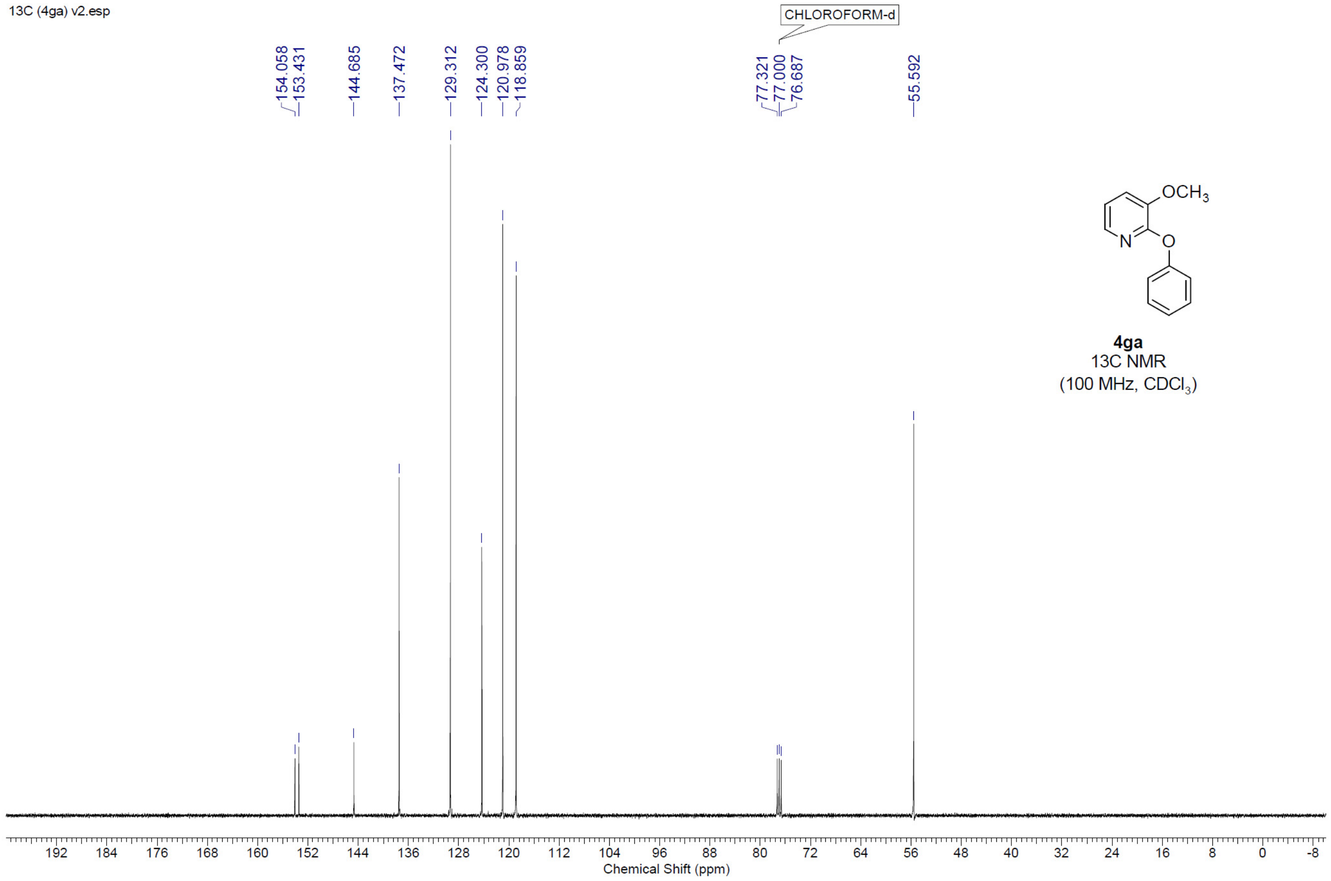
13C (4ga) v2.esp
CHLOROFORM-d
154.058
153.431
144.685
137.472
129.312
124.300
120.978
118.859
77.321
77.000
76.687
55.592
OCH3
N
O
4ga
13C NMR
(100 MHz, CDCl3)
192
184
176
168
160
152
144
136
128
120
112
104
96
88
80
72
64
56
48
40
32
24
16
8
0
-8
Chemical Shift (ppm)

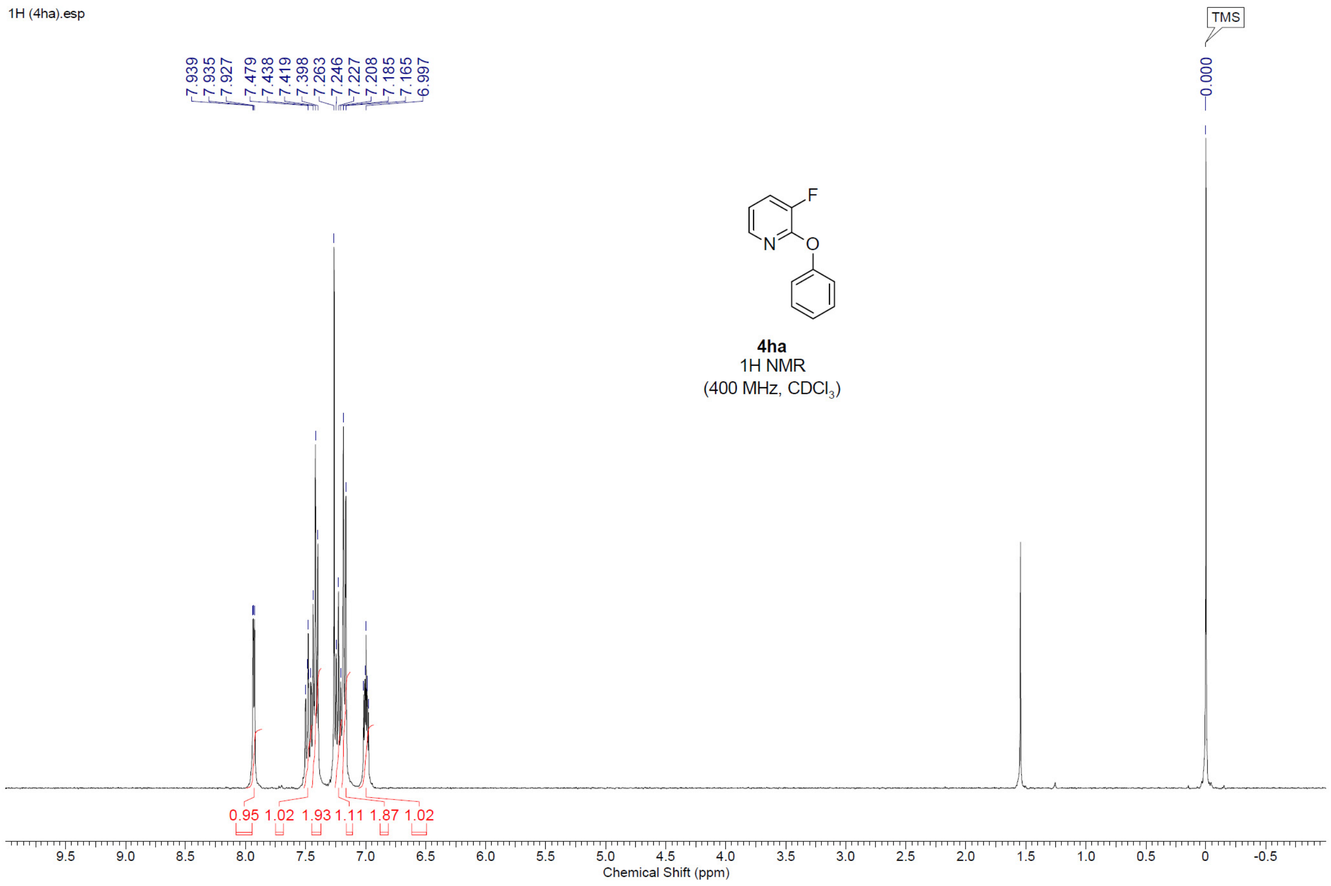
1H (4ha).esp
7.939
7.935
7.927
7.479
7.438
7.419
7.398
7.263
7.246
7.227
7.208
7.185
7.165
6.997
TMS
0.000
F
N
O
4ha
1H NMR
(400 MHz, CDCl3)
0.95 1.02 1.93 1.11 1.87 1.02
Chemical Shift (ppm)

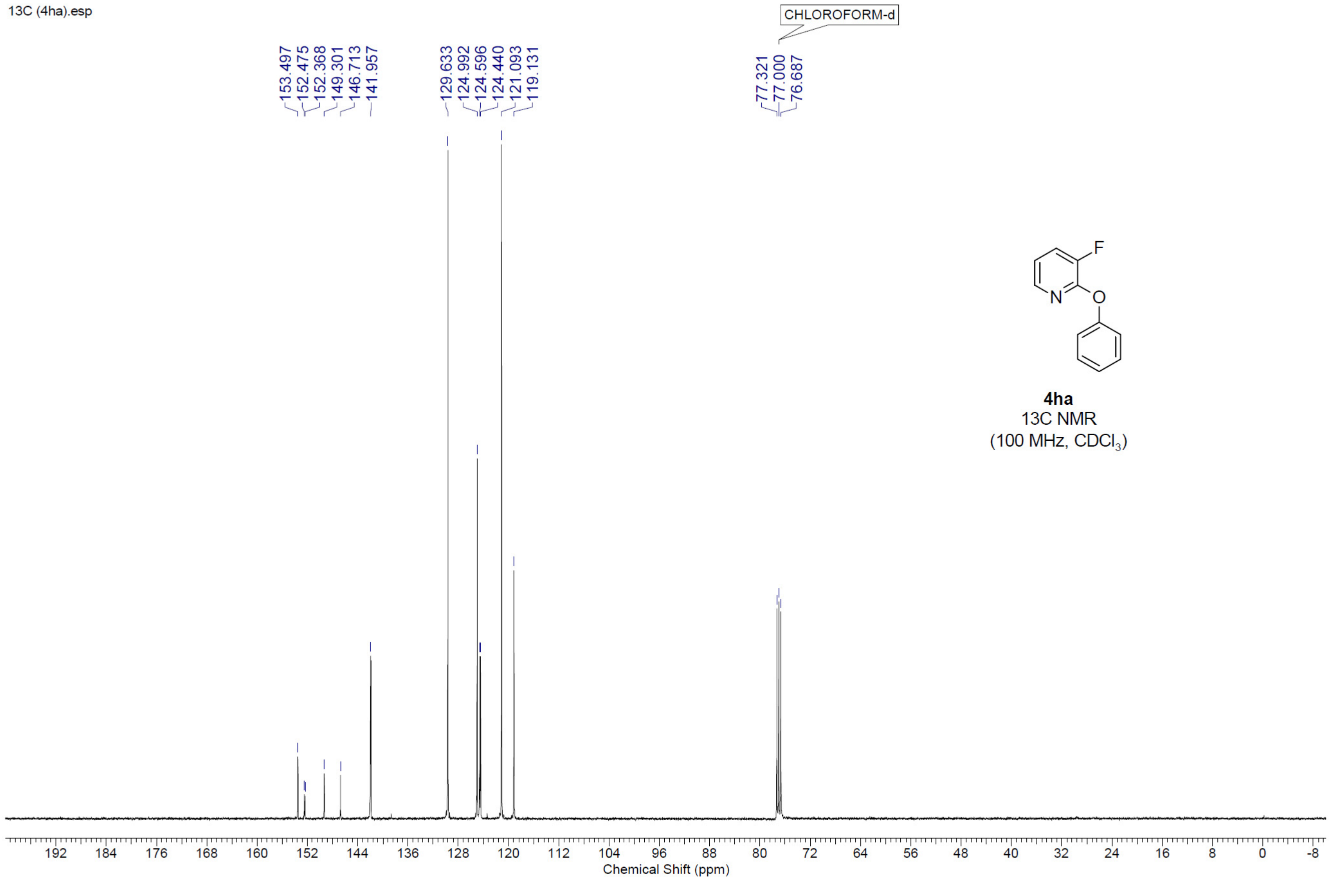
13C (4ha).esp
153.497
152.475
152.368
149.301
146.713
141.957
129.633
124.992
124.596
124.440
121.093
119.131
CHLOROFORM-d
77.321
77.000
76.687
F
N
O
4ha
13C NMR
(100 MHz, CDCl3)
Chemical Shift (ppm)

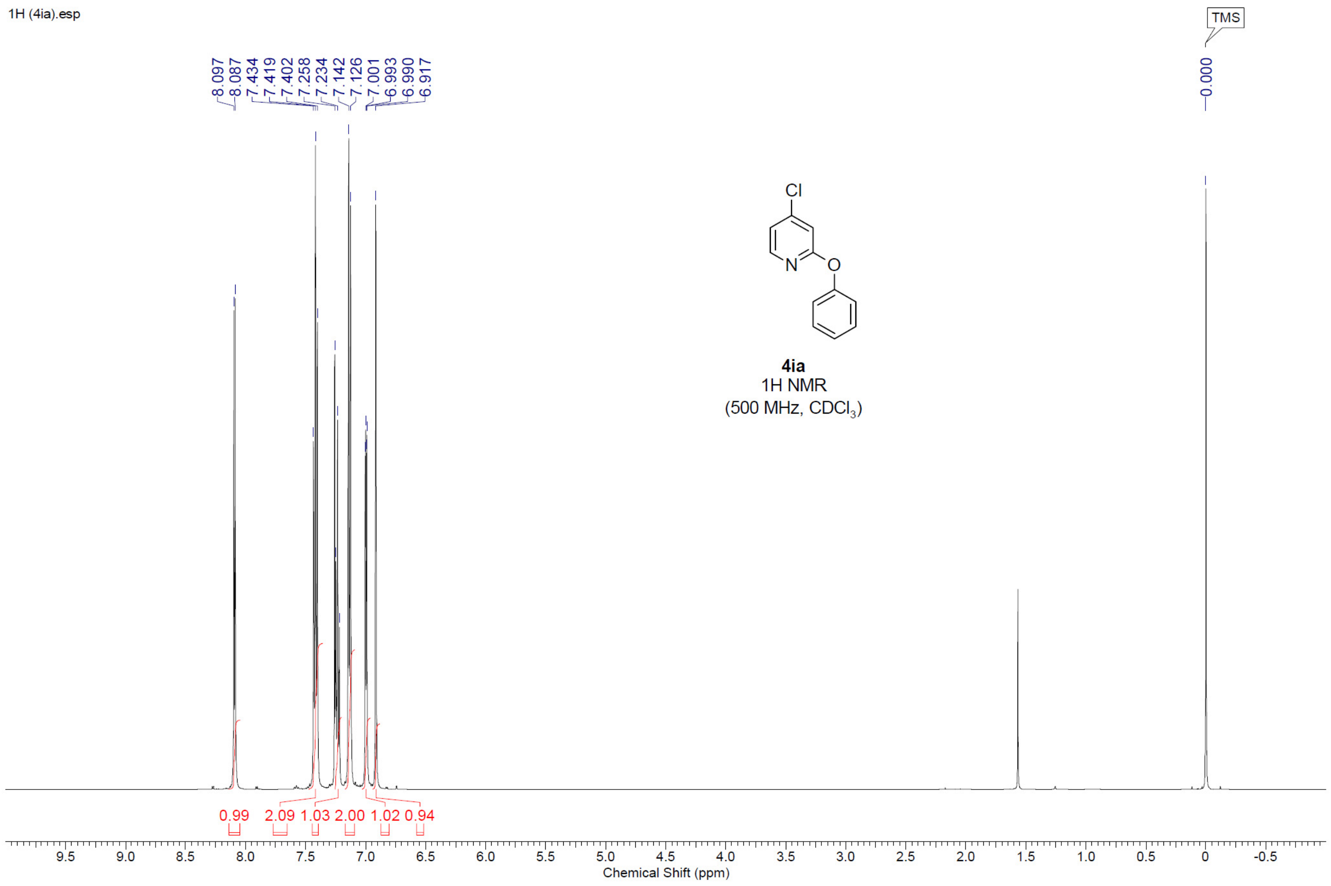
1H (4ia).esp
TMS
8.097
8.087
7.434
7.419
7.402
7.258
7.234
7.142
7.126
7.001
6.993
6.990
6.917
0.000
4ia
1H NMR
(500 MHz, CDCl3)
0.99
2.09
1.03
2.00
1.02
0.94
Chemical Shift (ppm)

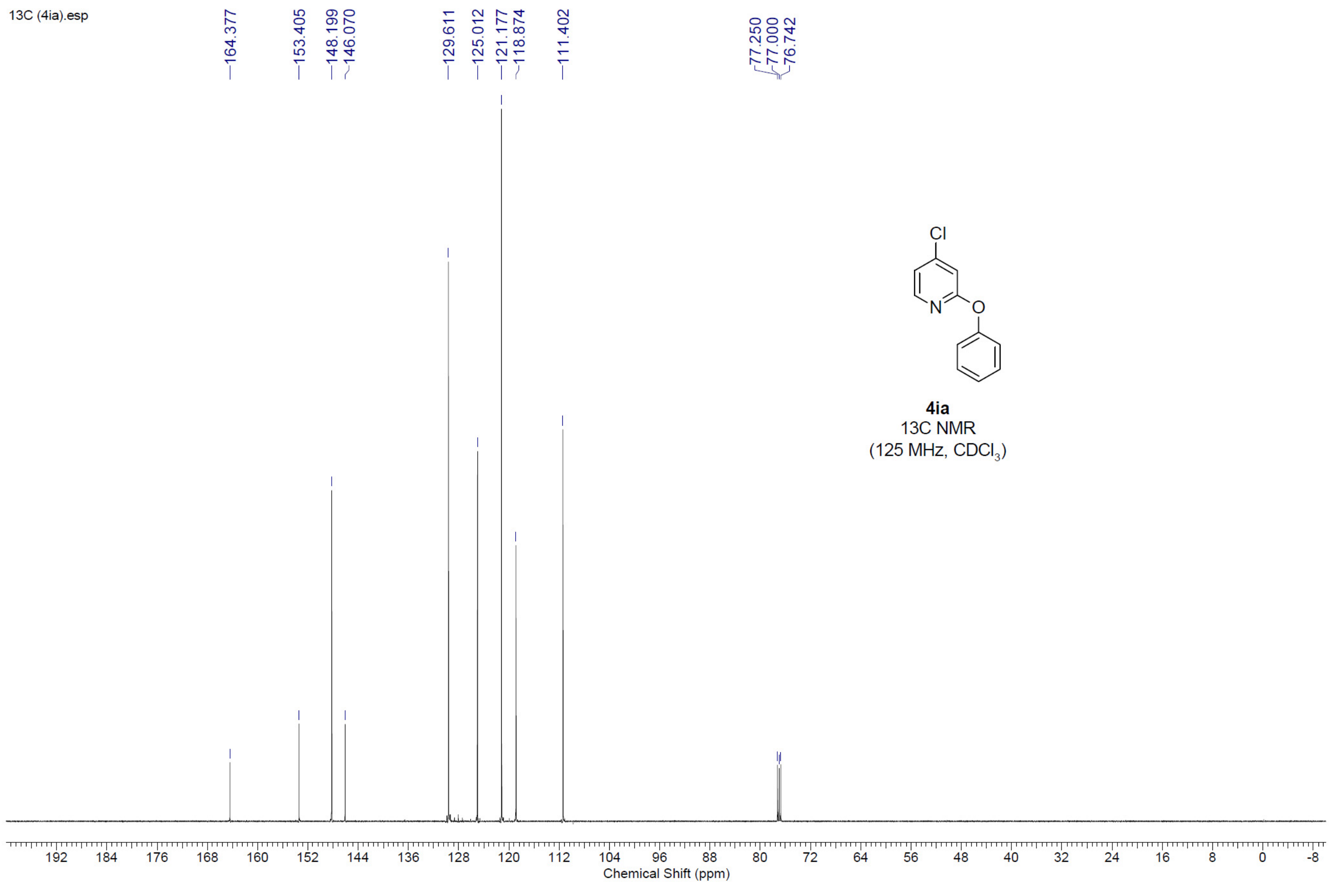
13C (4ia).esp
164.377
153.405
148.199
146.070
129.611
125.012
121.177
118.874
111.402
77.250
77.000
76.742
4ia
13C NMR
(125 MHz, CDCl3)
Chemical Shift (ppm)

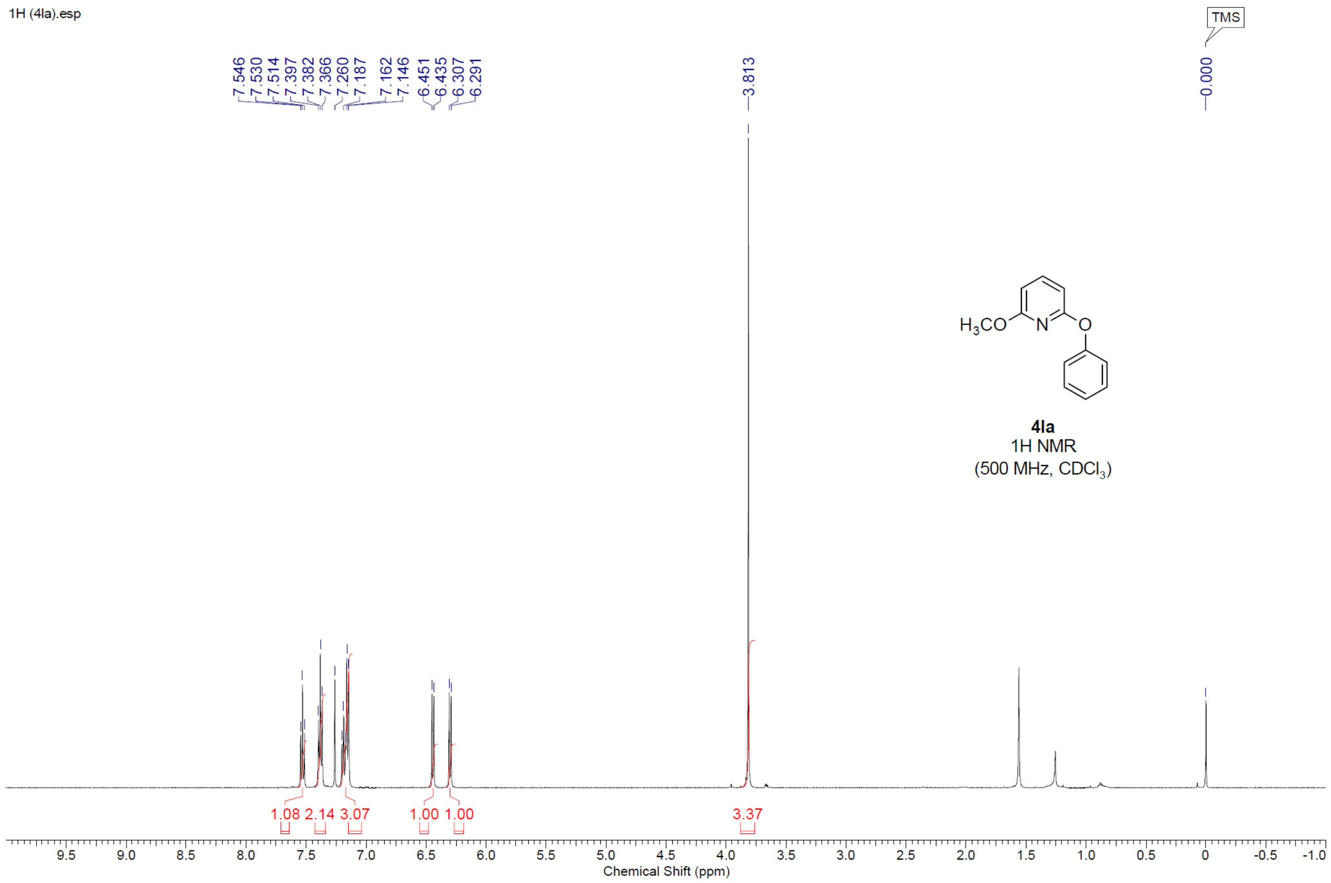
1H (4la).esp
7.546
7.530
7.514
7.397
7.382
7.366
7.260
7.187
7.162
7.146
6.451
6.435
6.307
6.291
3.813
0.000
TMS
H3CO
N
O
4la
1H NMR
(500 MHz, CDCl3)
1.08
2.14
3.07
1.00
1.00
3.37
Chemical Shift (ppm)

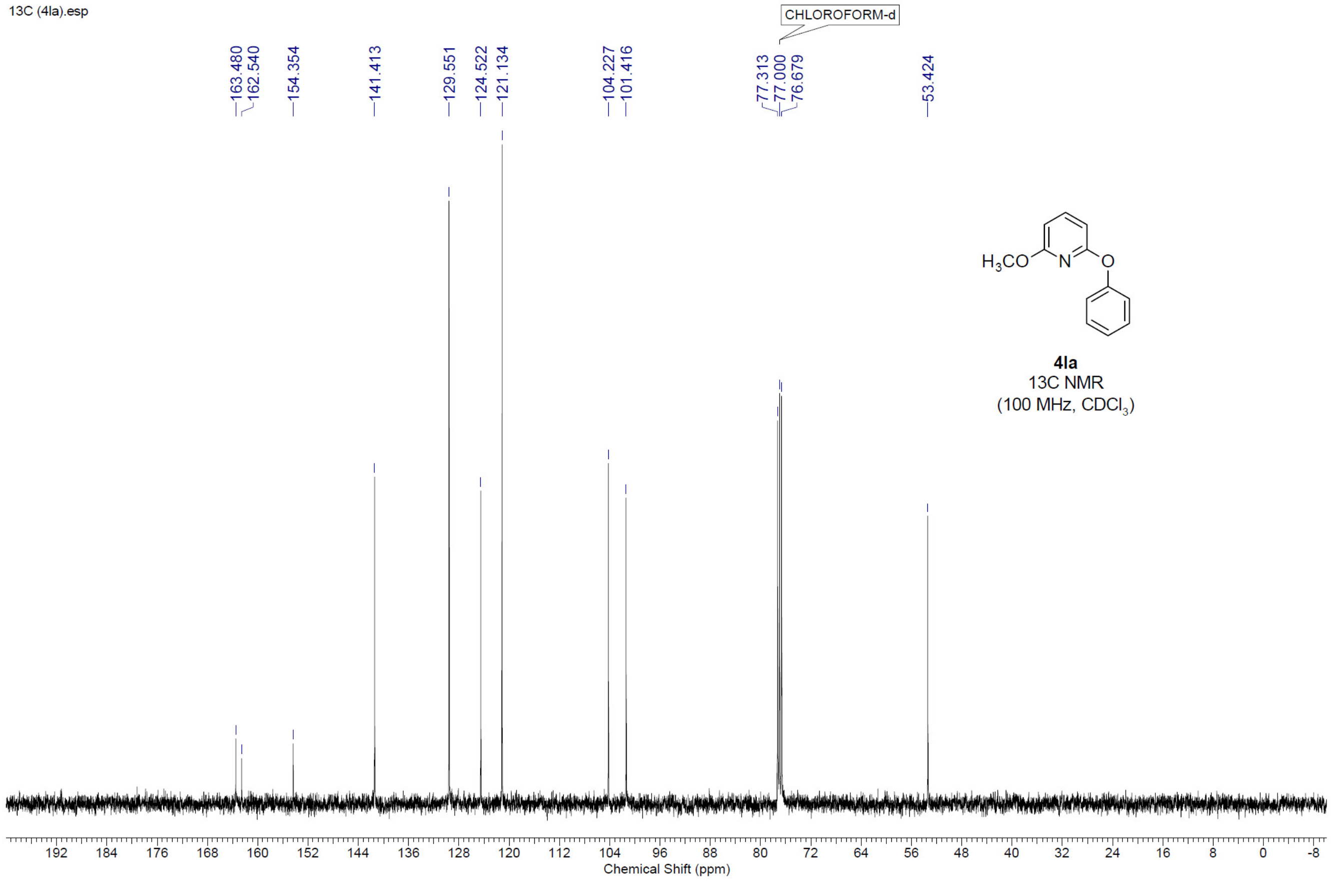
13C (4la).esp
163.480
162.540
154.354
141.413
129.551
124.522
121.134
104.227
101.416
77.313
77.000
76.679
53.424
CHLOROFORM-d
H3CO
N
O
4la
13C NMR
(100 MHz, CDCl3)
Chemical Shift (ppm)

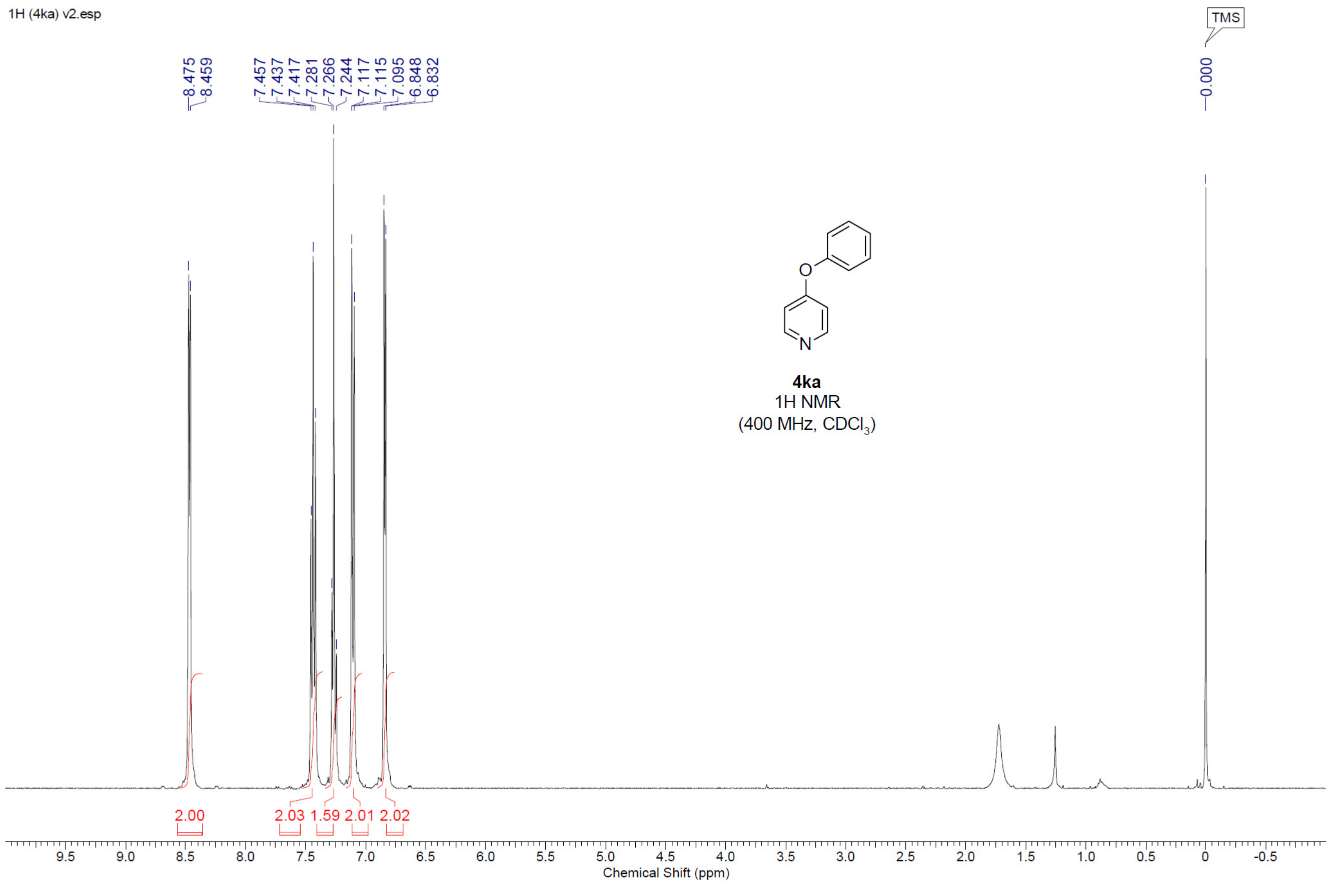
1H (4ka) v2.esp
8.475
8.459
7.457
7.437
7.417
7.281
7.266
7.244
7.117
7.115
7.095
6.848
6.832
TMS
0.000
O
N
4ka
1H NMR
(400 MHz, CDCl3)
2.00
2.03
1.59
2.01
2.02
9.5
9.0
8.5
8.0
7.5
7.0
6.5
6.0
5.5
5.0
4.5
4.0
3.5
3.0
2.5
2.0
1.5
1.0
0.5
0
-0.5
Chemical Shift (ppm)

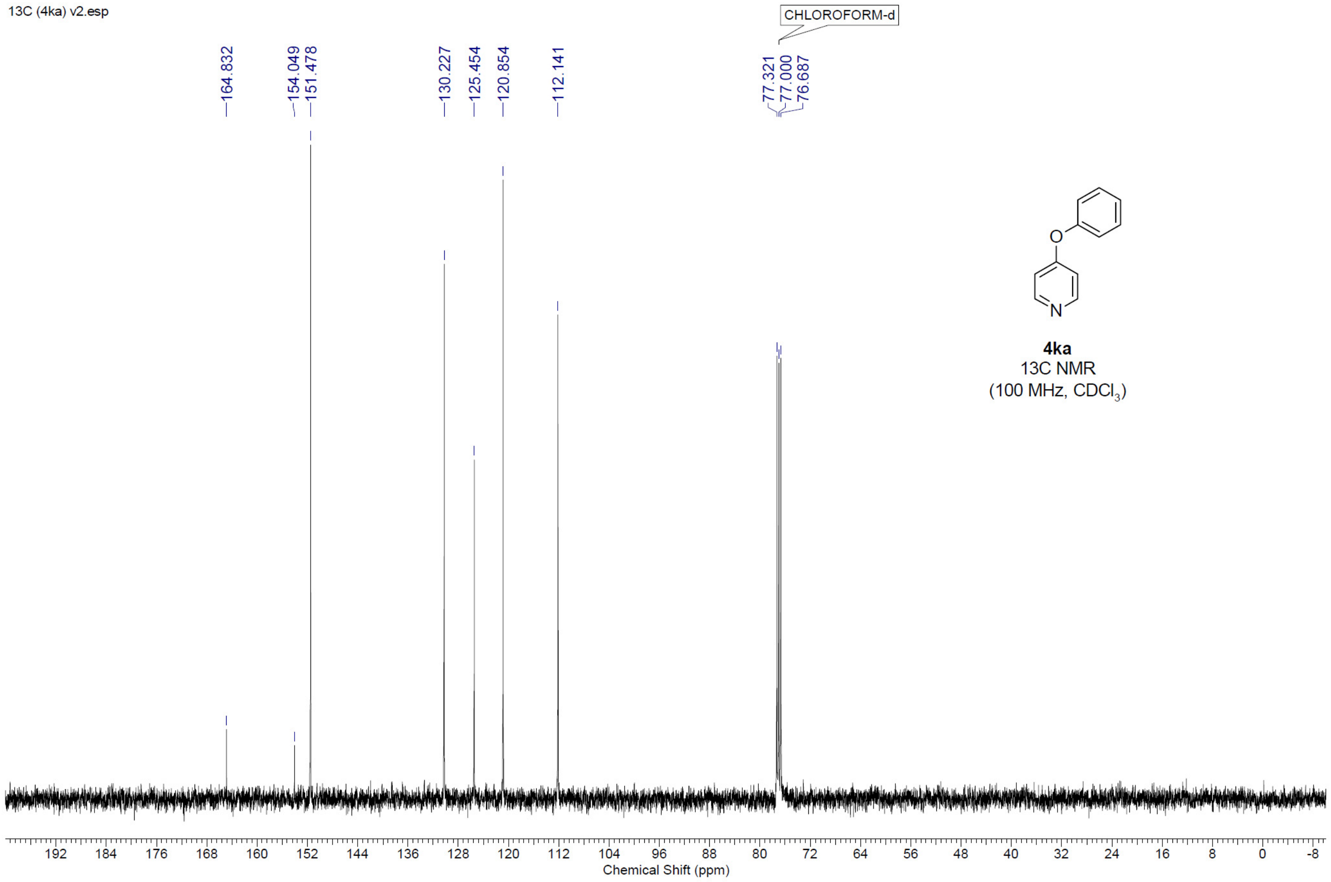
13C (4ka) v2.esp
164.832
154.049
151.478
130.227
125.454
120.854
112.141
CHLOROFORM-d
77.321
77.000
76.687
O
N
4ka
13C NMR
(100 MHz, CDCl3)
192
184
176
168
160
152
144
136
128
120
112
104
96
88
80
72
64
56
48
40
32
24
16
8
0
-8
Chemical Shift (ppm)

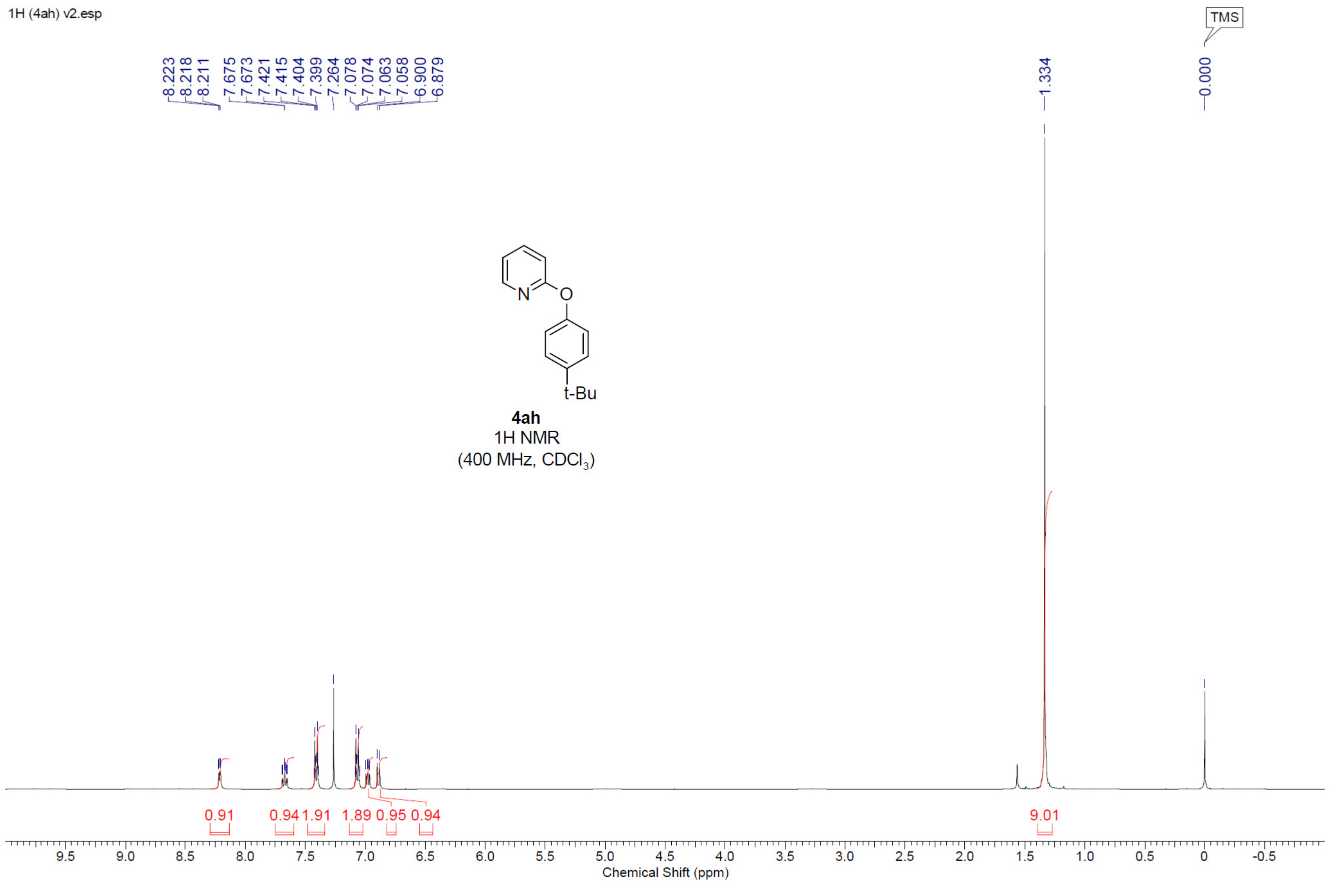
1H (4ah) v2.esp
TMS
8.223
8.218
8.211
7.675
7.673
7.421
7.415
7.404
7.399
7.264
7.078
7.074
7.063
7.058
6.900
6.879
1.334
0.000
N
O
t-Bu
4ah
1H NMR
(400 MHz, CDCl3)
0.91
0.94
1.91
1.89
0.95
0.94
9.01
Chemical Shift (ppm)

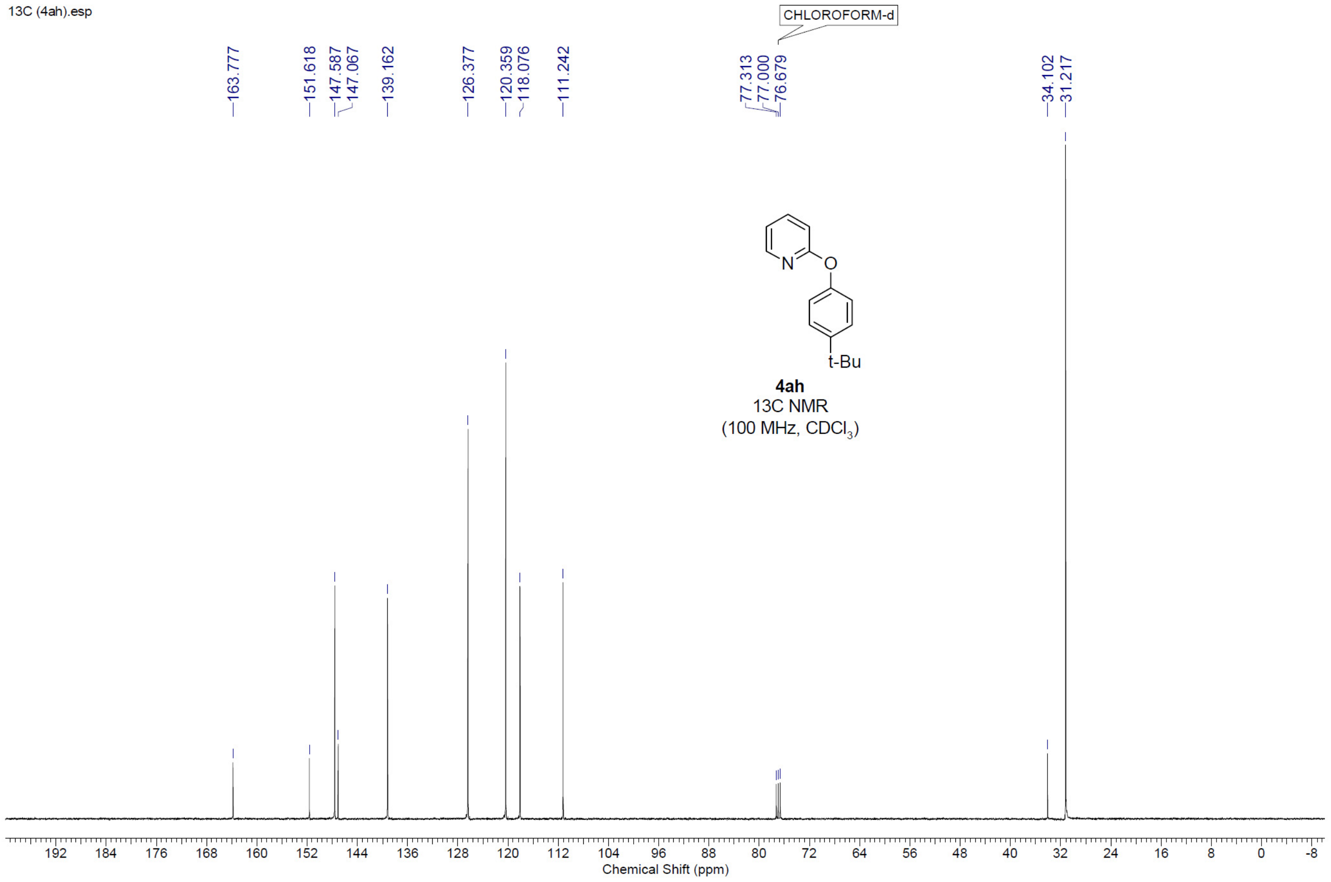
13C (4ah).esp
CHLOROFORM-d
163.777
151.618
147.587
147.067
139.162
126.377
120.359
118.076
111.242
77.313
77.000
76.679
34.102
31.217
N
O
t-Bu
4ah
13C NMR
(100 MHz, CDCl3)
Chemical Shift (ppm)

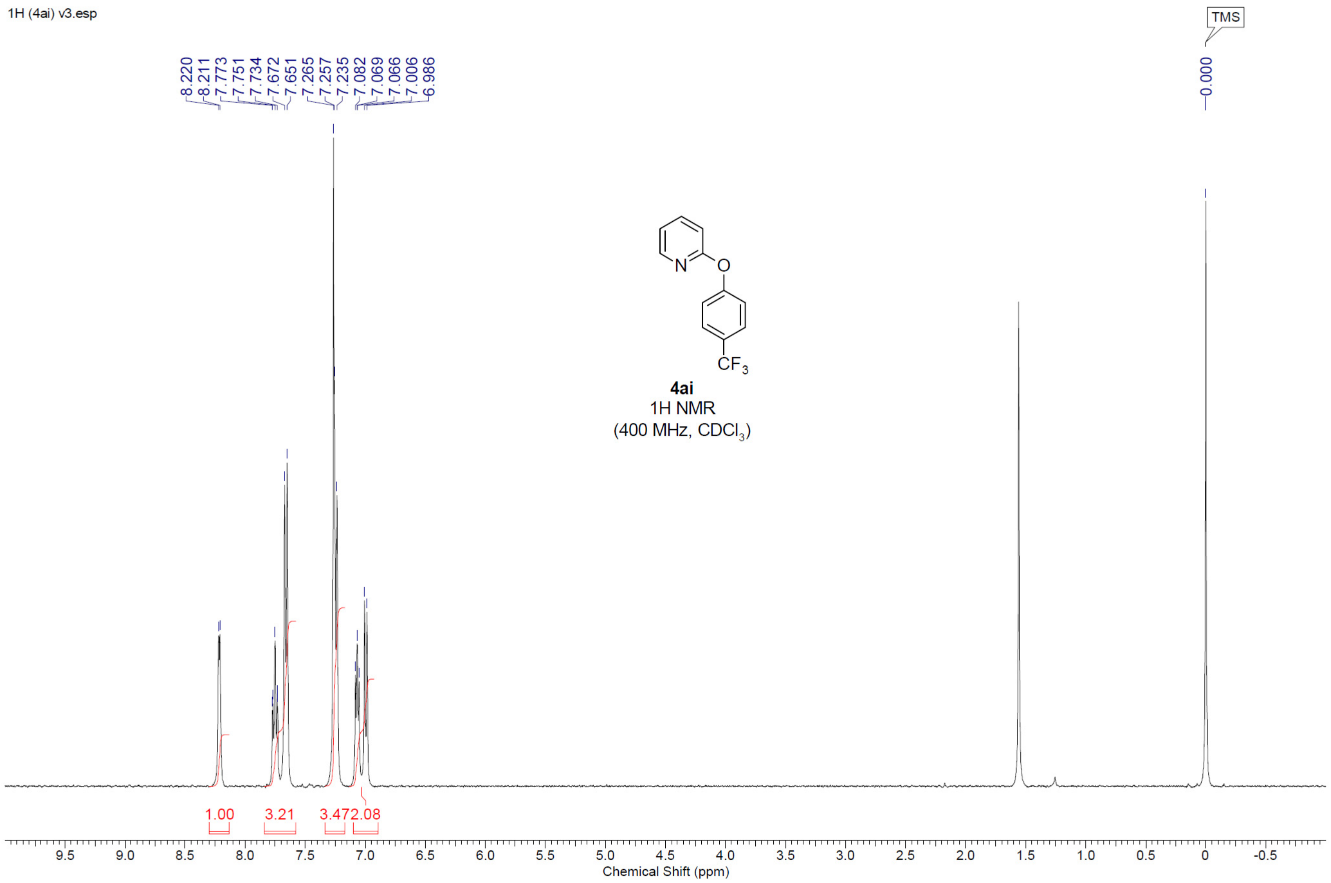
1H (4ai) v3.esp
TMS
8.220
8.211
7.773
7.751
7.734
7.672
7.651
7.265
7.257
7.235
7.082
7.069
7.066
7.006
6.986
0.000
4ai
1H NMR
(400 MHz, CDCl3)
1.00
3.21
3.47
2.08
Chemical Shift (ppm)

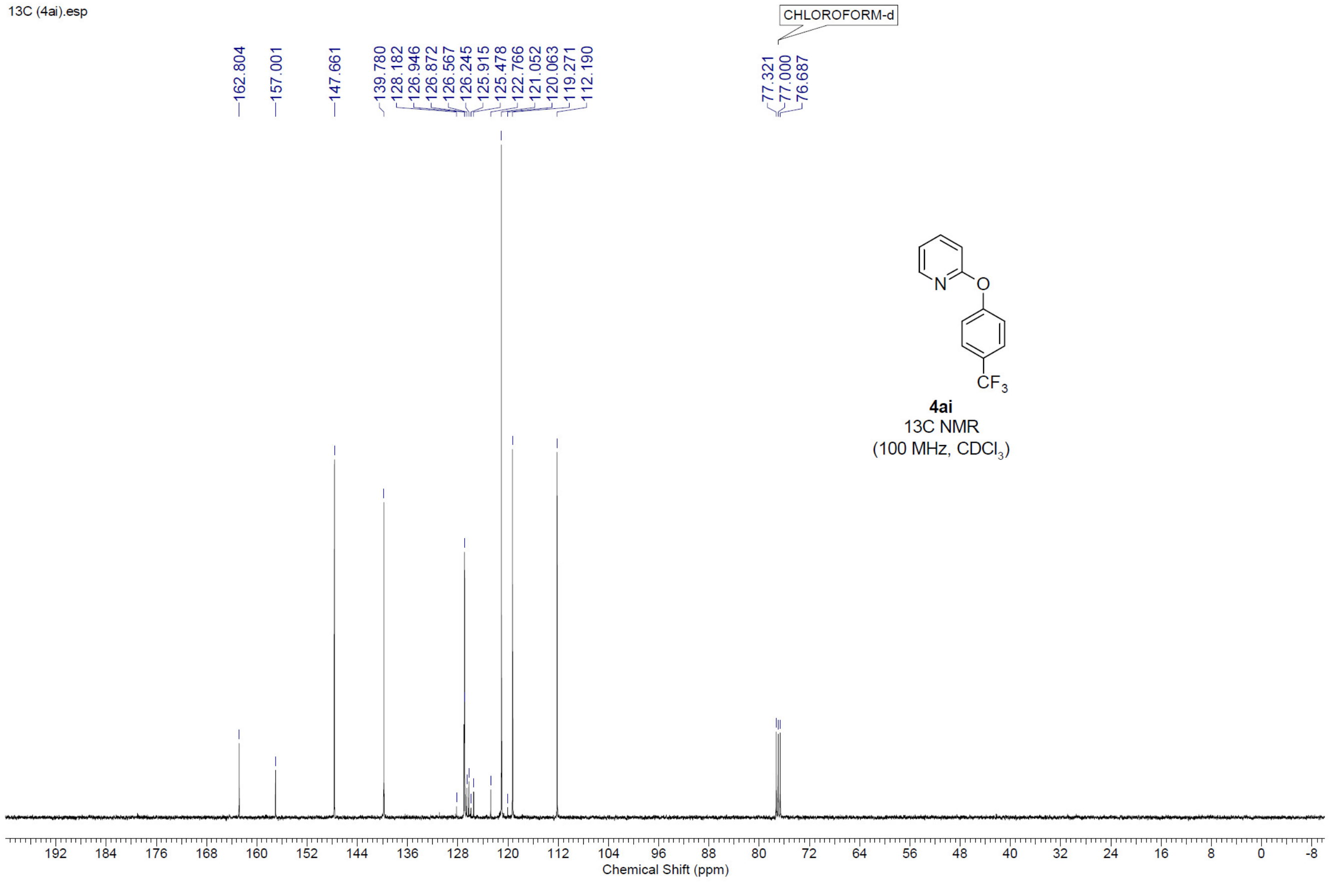
13C (4ai).esp
CHLOROFORM-d
162.804
157.001
147.661
139.780
128.182
126.946
126.872
126.567
126.245
125.915
125.478
122.766
121.052
120.063
119.271
112.190
77.321
77.000
76.687
4ai
13C NMR
(100 MHz, CDCl3)
Chemical Shift (ppm)

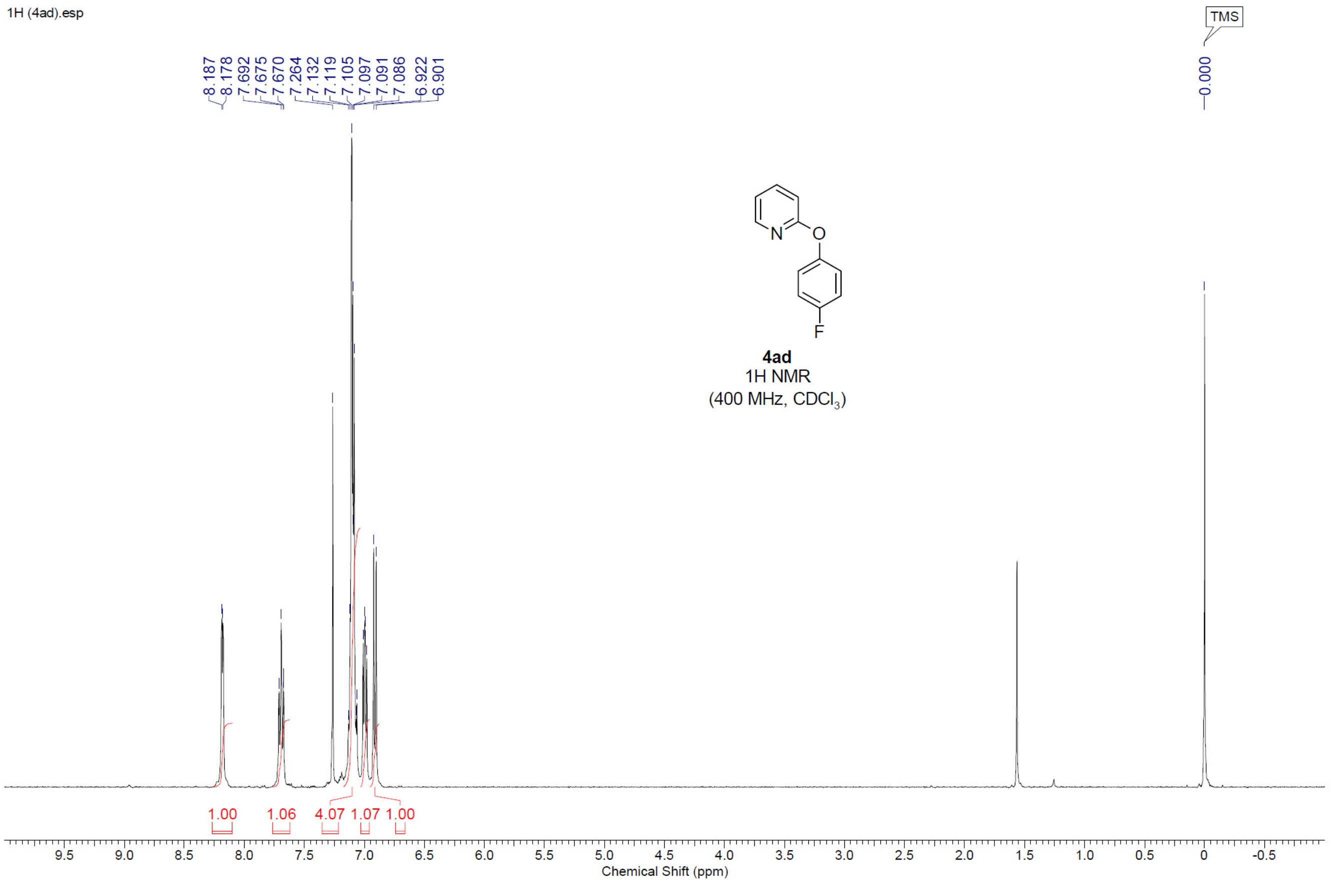
1H (4ad).esp
TMS
8.187
8.178
7.692
7.675
7.670
7.264
7.132
7.119
7.105
7.097
7.091
7.086
6.922
6.901
0.000
N
O
F
4ad
1H NMR
(400 MHz, CDCl3)
1.00
1.06
4.07
1.07
1.00
Chemical Shift (ppm)

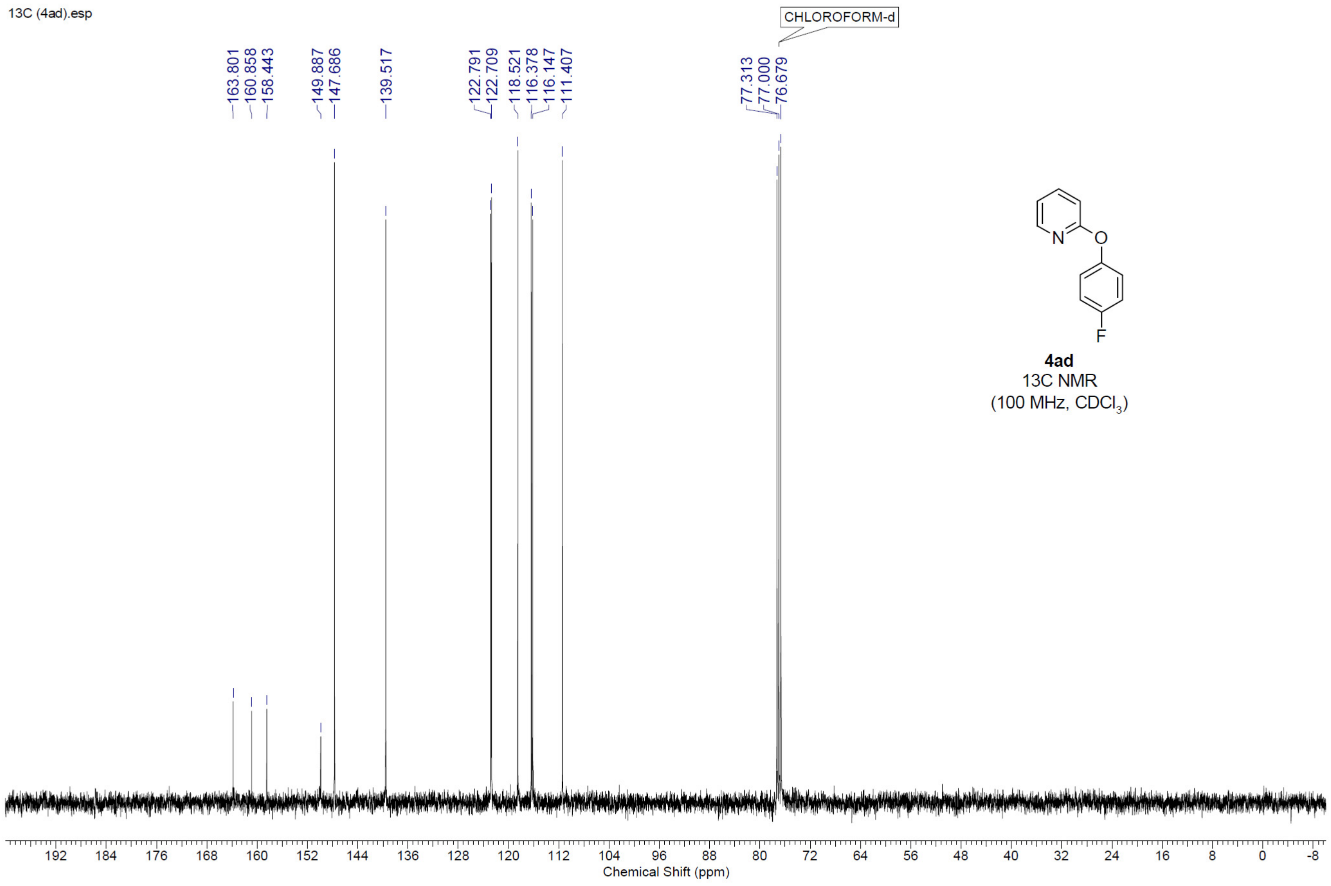
13C (4ad).esp
CHLOROFORM-d
163.801
160.858
158.443
149.887
147.686
139.517
122.791
122.709
118.521
116.378
116.147
111.407
77.313
77.000
76.679
N
O
F
4ad
13C NMR
(100 MHz, CDCl3)
Chemical Shift (ppm)

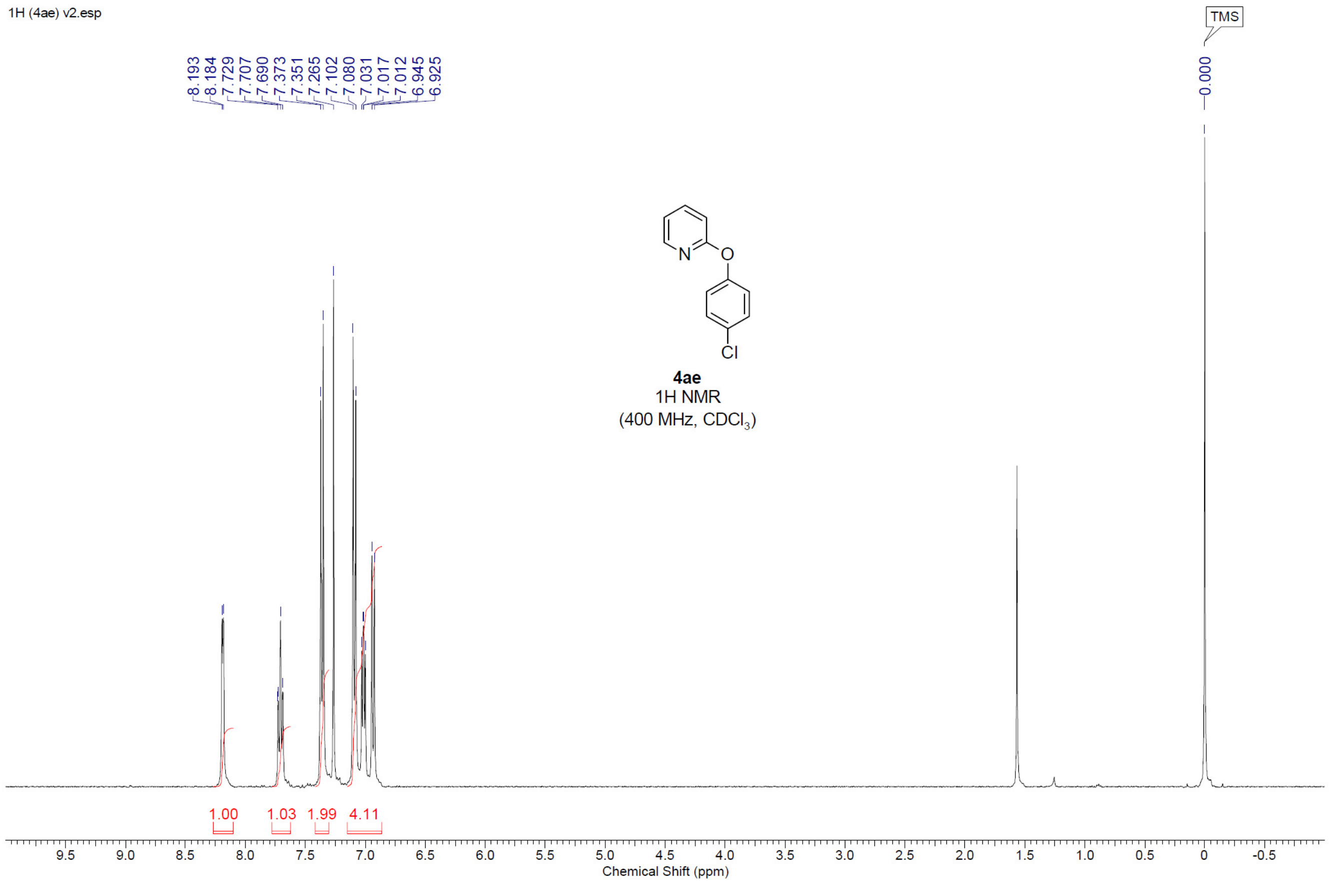
1H (4ae) v2.esp
TMS
8.193
8.184
7.729
7.707
7.690
7.373
7.351
7.265
7.102
7.080
7.031
7.017
7.012
6.945
6.925
0.000
N
O
Cl
4ae
1H NMR
(400 MHz, CDCl3)
1.00
1.03
1.99
4.11
9.5 9.0 8.5 8.0 7.5 7.0 6.5 6.0 5.5 5.0 4.5 4.0 3.5 3.0 2.5 2.0 1.5 1.0 0.5 0 -0.5
Chemical Shift (ppm)

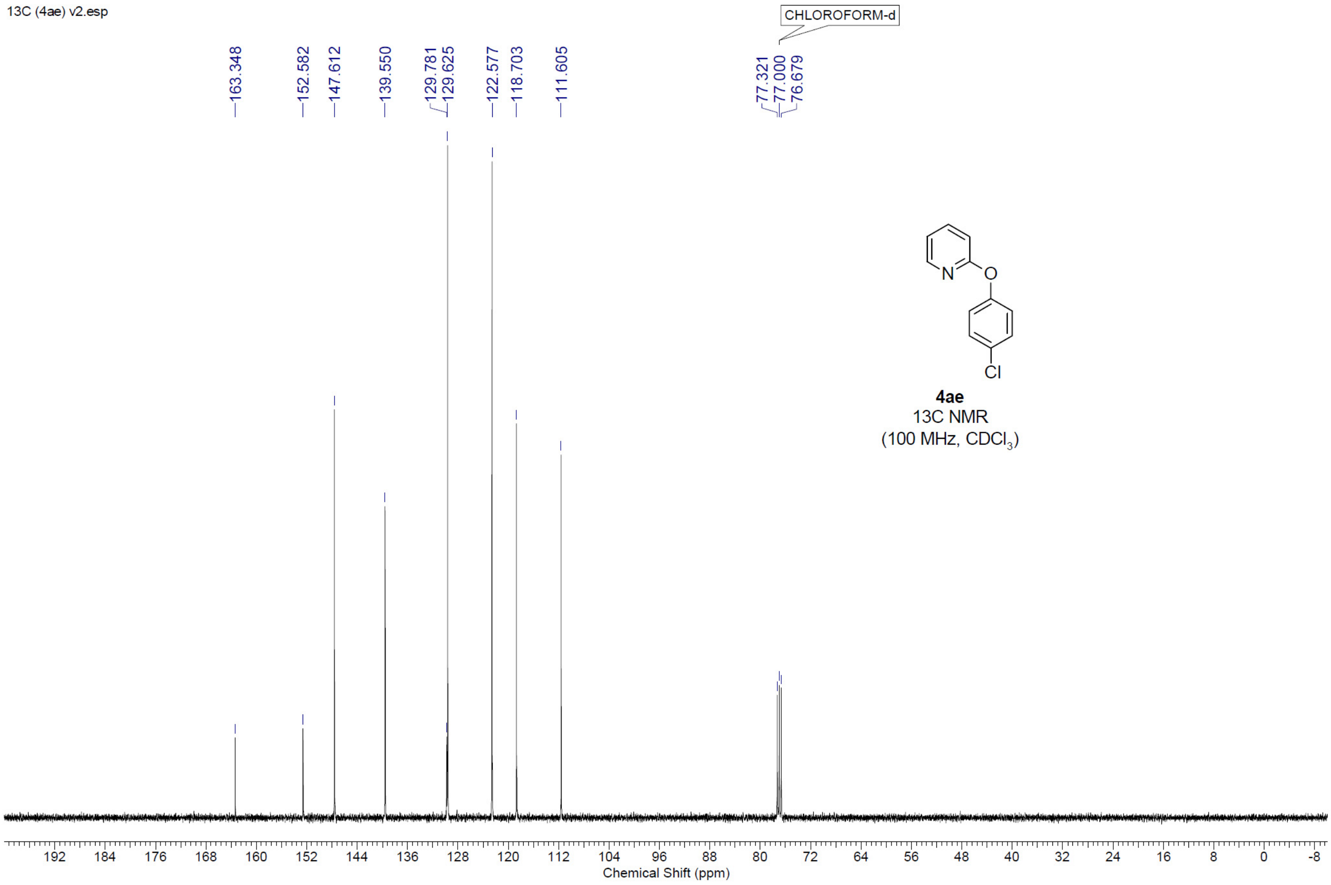
13C (4ae) v2.esp
CHLOROFORM-d
163.348
152.582
147.612
139.550
129.781
129.625
122.577
118.703
111.605
77.321
77.000
76.679
N
O
Cl
4ae
13C NMR
(100 MHz, CDCl3)
192 184 176 168 160 152 144 136 128 120 112 104 96 88 80 72 64 56 48 40 32 24 16 8 0 -8
Chemical Shift (ppm)

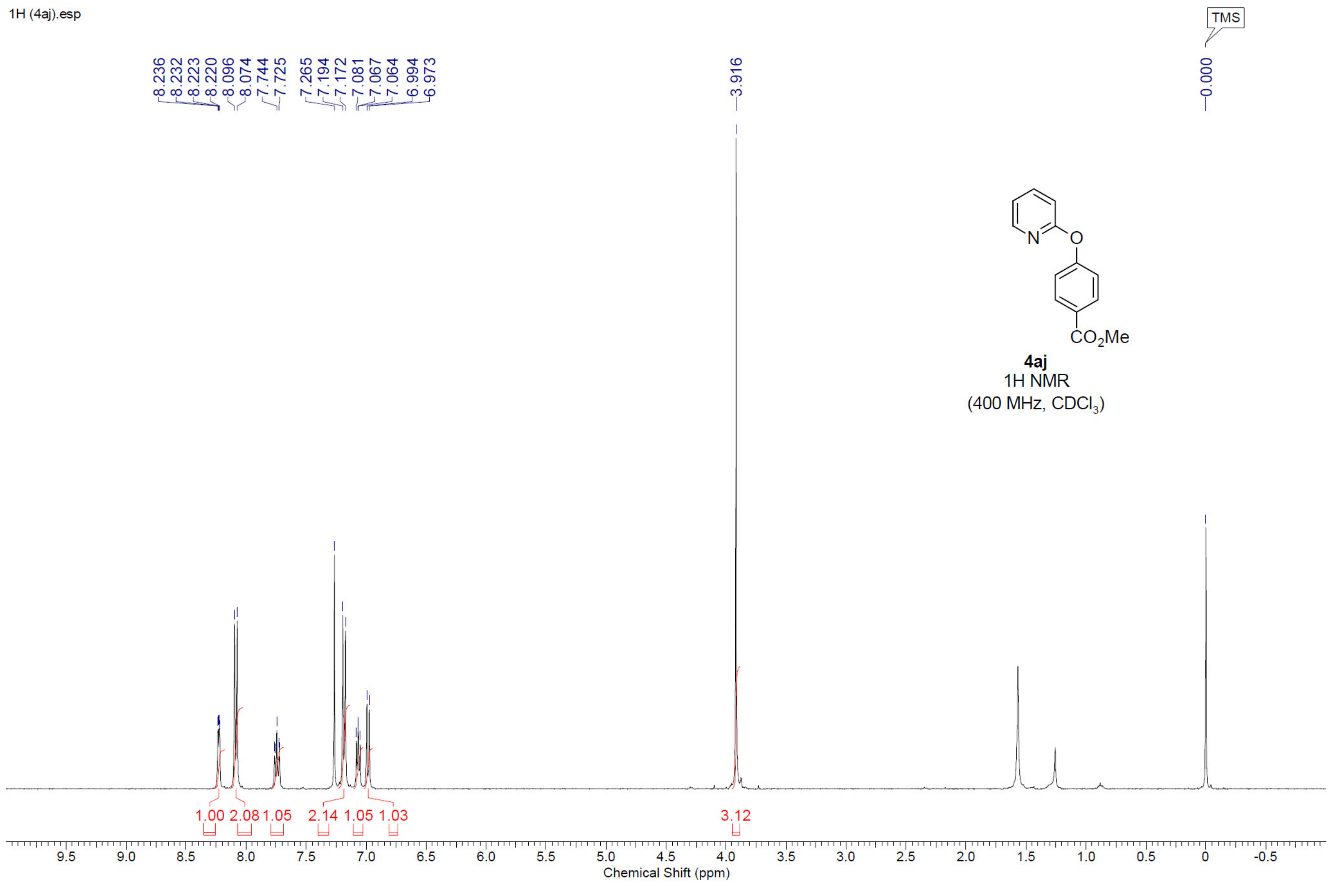
1H (4aj).esp
TMS
8.236
8.232
8.223
8.220
8.096
8.074
7.744
7.725
7.265
7.194
7.172
7.081
7.067
7.064
6.994
6.973
3.916
0.000
N
O
CO_2Me
4aj
1H NMR
(400 MHz, $CDCl_3$)
1.00 2.08 1.05 2.14 1.05 1.03
3.12
Chemical Shift (ppm)

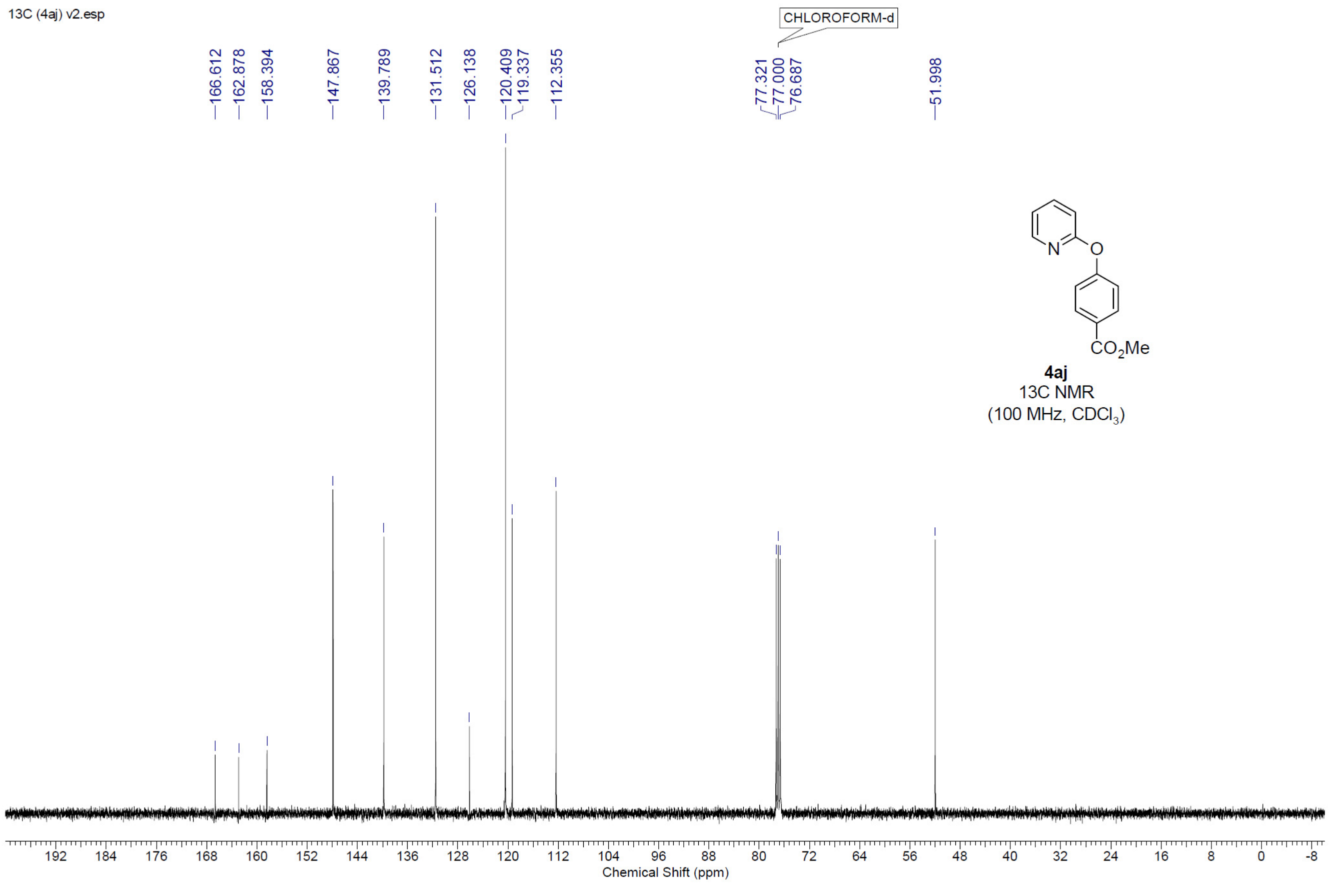
13C (4aj) v2.esp
CHLOROFORM-d
166.612
162.878
158.394
147.867
139.789
131.512
126.138
120.409
119.337
112.355
77.321
77.000
76.687
51.998
N
O
CO_2Me
4aj
13C NMR
(100 MHz, $CDCl_3$)
Chemical Shift (ppm)

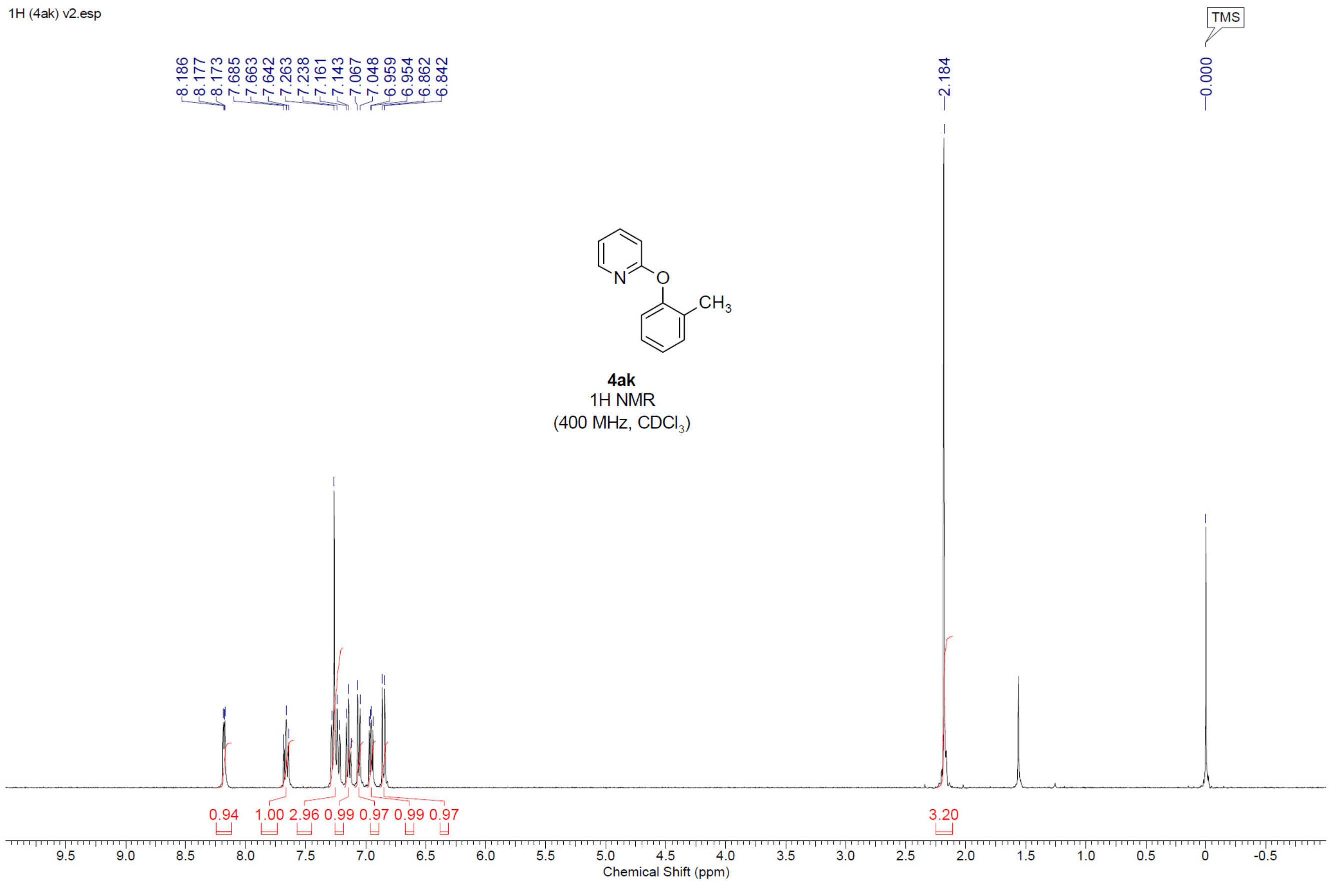
1H (4ak) v2.esp
TMS
8.186
8.177
8.173
7.685
7.663
7.642
7.263
7.238
7.161
7.143
7.067
7.048
6.959
6.954
6.862
6.842
2.184
0.000
4ak
1H NMR
(400 MHz, CDCl3)
0.94
1.00
2.96
0.99
0.97
0.99
0.97
3.20
Chemical Shift (ppm)

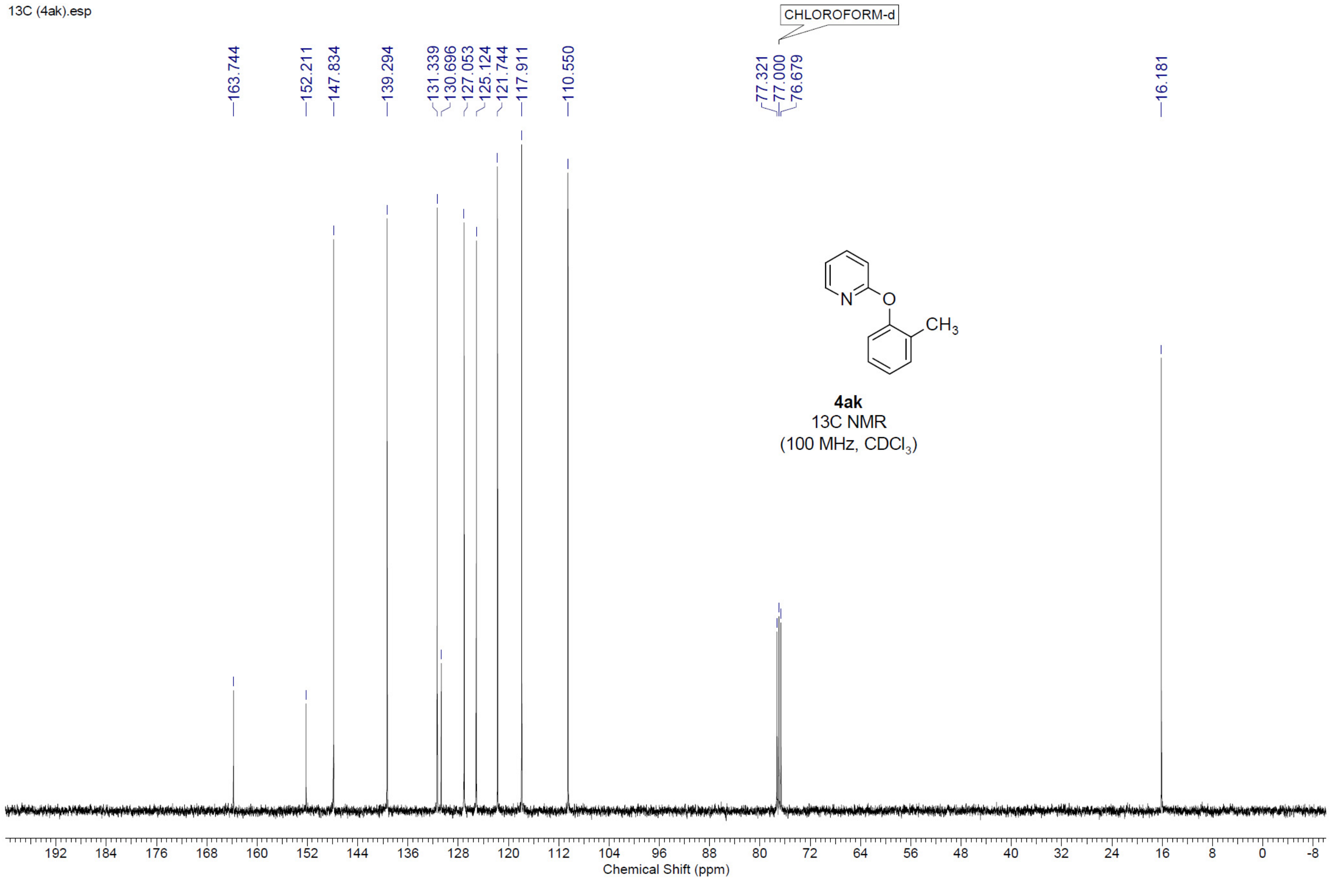
13C (4ak).esp
CHLOROFORM-d
163.744
152.211
147.834
139.294
131.339
130.696
127.053
125.124
121.744
117.911
110.550
77.321
77.000
76.679
16.181
4ak
13C NMR
(100 MHz, CDCl3)
Chemical Shift (ppm)

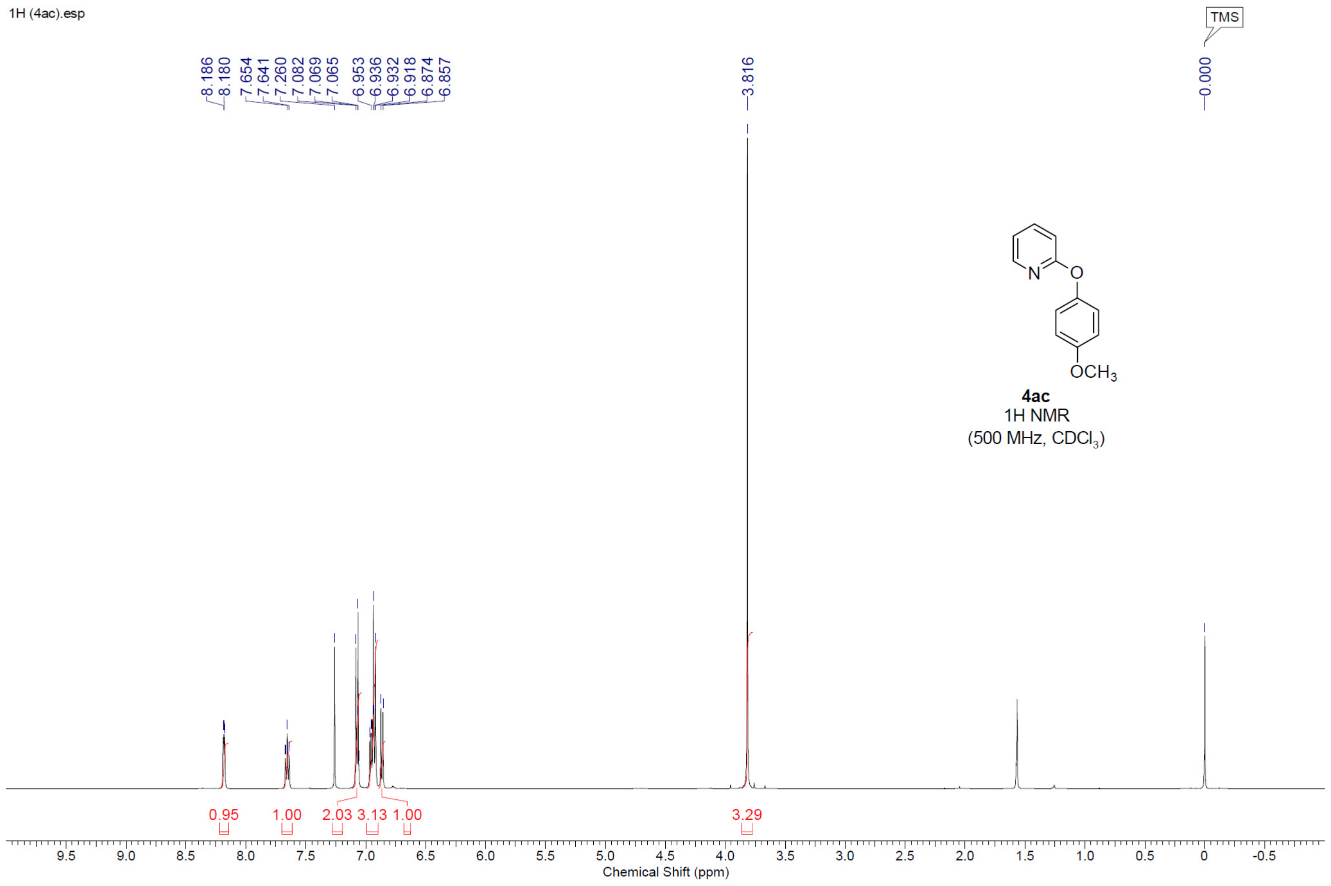
1H (4ac).esp
TMS
8.186
8.180
7.654
7.641
7.260
7.082
7.069
7.065
6.953
6.936
6.932
6.918
6.874
6.857
3.816
0.000
N
O
OCH3
4ac
1H NMR
(500 MHz, CDCl3)
0.95
1.00
2.03
3.13
1.00
3.29
Chemical Shift (ppm)

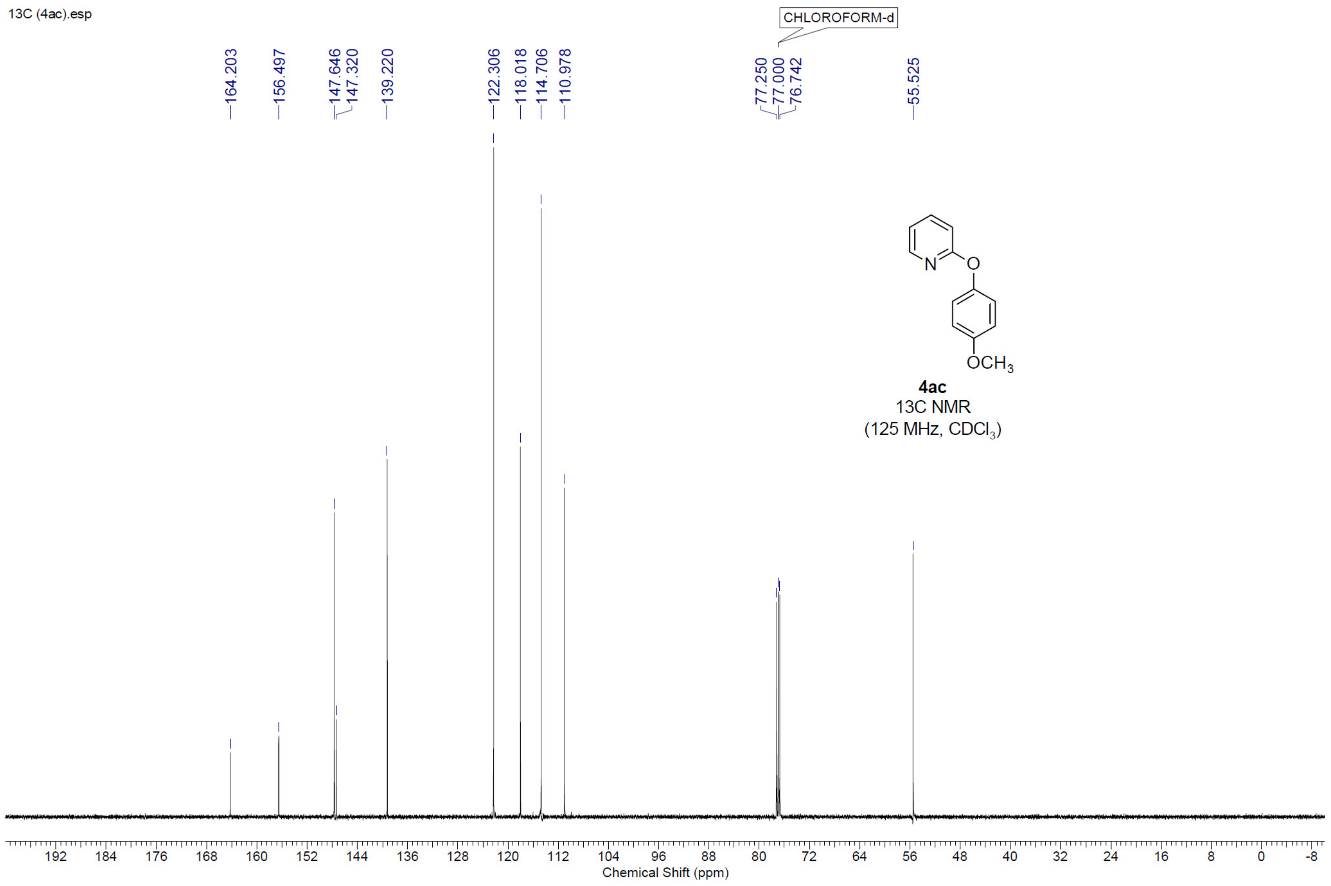
13C (4ac).esp
CHLOROFORM-d
164.203
156.497
147.646
147.320
139.220
122.306
118.018
114.706
110.978
77.250
77.000
76.742
55.525
N
O
OCH3
4ac
13C NMR
(125 MHz, CDCl3)
Chemical Shift (ppm)

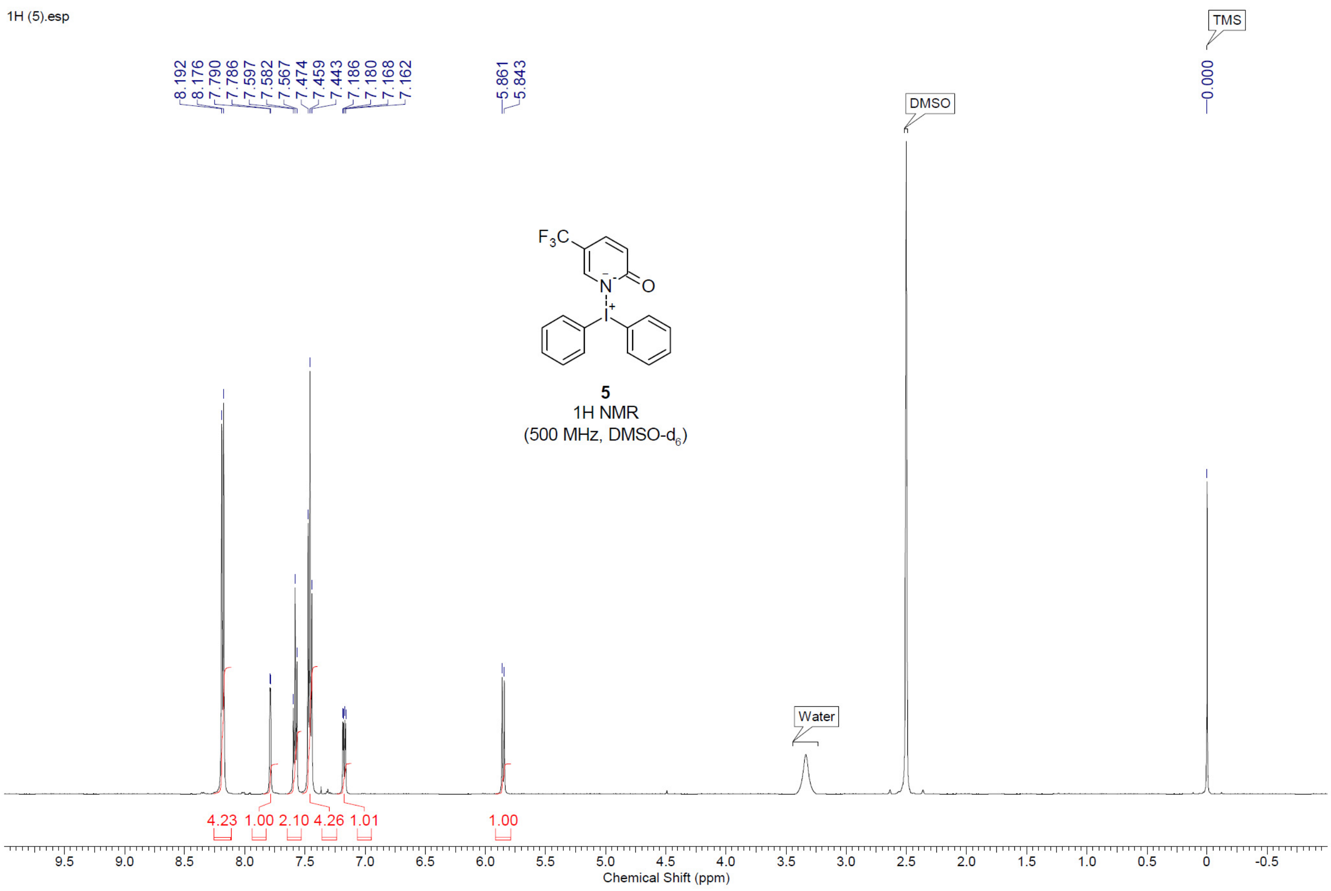
1H (5).esp
8.192
8.176
7.790
7.786
7.597
7.582
7.567
7.474
7.459
7.443
7.186
7.180
7.168
7.162
5.861
5.843
DMSO
TMS
-0.000
F3C
N
O
I
5
1H NMR
(500 MHz, DMSO-d6)
Water
4.23
1.00
2.10
4.26
1.01
1.00
Chemical Shift (ppm)

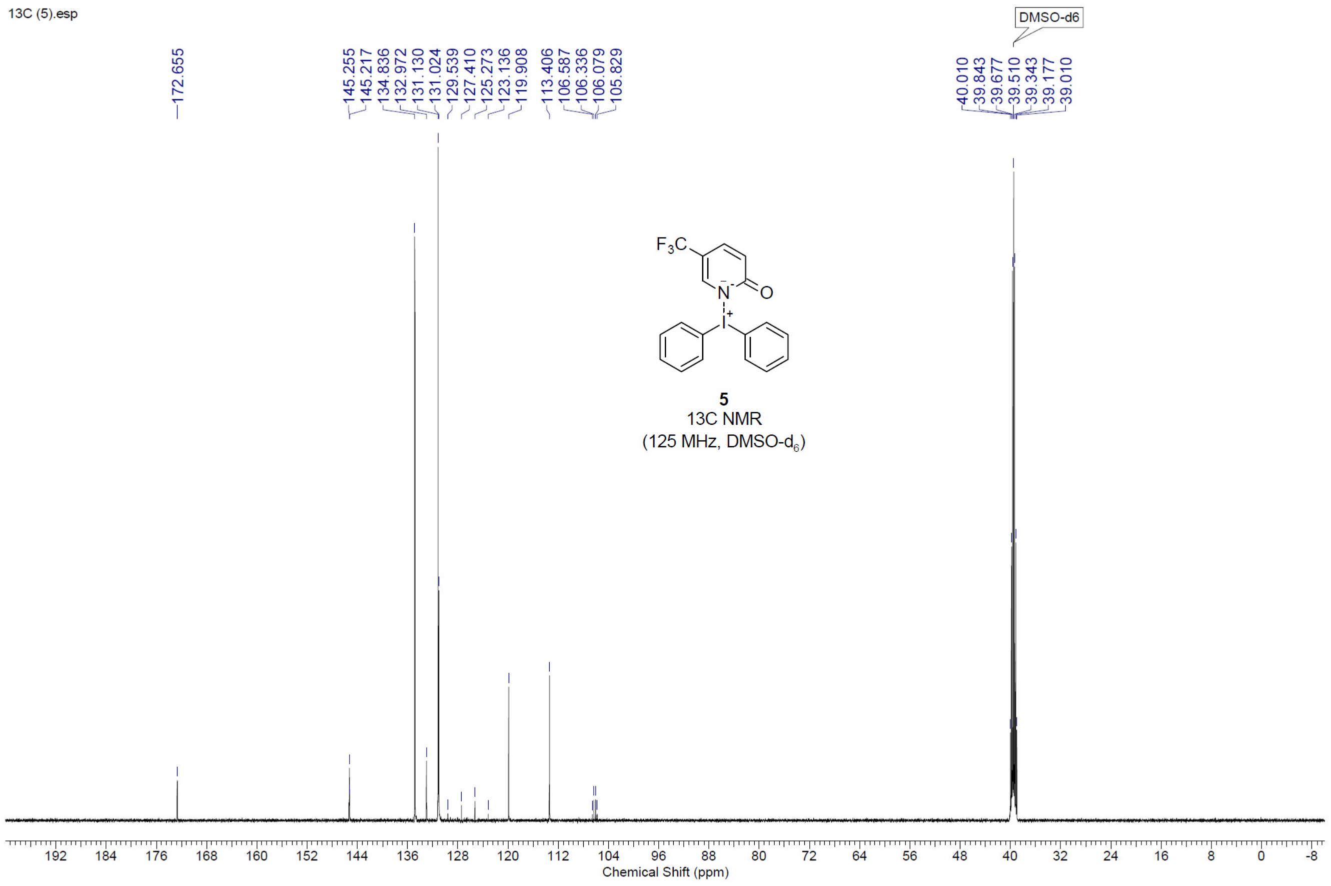
13C (5).esp
172.655
145.255
145.217
134.836
132.972
131.130
131.024
129.539
127.410
125.273
123.136
119.908
113.406
106.587
106.336
106.079
105.829
DMSO-d6
40.010
39.843
39.677
39.510
39.343
39.177
39.010
F3C
N
O
I
5
13C NMR
(125 MHz, DMSO-d6)
Chemical Shift (ppm)
